# Glutamate Acts as a Key Signal Linking Glucose Metabolism to Incretin/cAMP Action to Amplify Insulin Secretion

**DOI:** 10.1016/j.celrep.2014.09.030

**Published:** 2014-10-16

**Authors:** Ghupurjan Gheni, Masahito Ogura, Masahiro Iwasaki, Norihide Yokoi, Kohtaro Minami, Yasumune Nakayama, Kazuo Harada, Benoit Hastoy, Xichen Wu, Harumi Takahashi, Kazushi Kimura, Toshiya Matsubara, Ritsuko Hoshikawa, Naoya Hatano, Kenji Sugawara, Tadao Shibasaki, Nobuya Inagaki, Takeshi Bamba, Akira Mizoguchi, Eiichiro Fukusaki, Patrik Rorsman, Susumu Seino

**Affiliations:** 1 Division of Molecular and Metabolic Medicine, Kobe University Graduate School of Medicine, Chuo-ku, Kobe 650-0017, Japan; 2 Division of Diabetes and Endocrinology, Kobe University Graduate School of Medicine, Chuo-ku, Kobe 650-0017, Japan; 3 Division of Cellular and Molecular Medicine, Kobe University Graduate School of Medicine, Chuo-ku, Kobe 650-0017, Japan; 4 The Integrated Center for Mass Spectrometry, Kobe University Graduate School of Medicine, Chuo-ku, Kobe 650-0017, Japan; 5 Department of Diabetes, Endocrinology and Nutrition, Graduate School of Medicine, Kyoto University, Sakyo-ku, Kyoto 606–8507, Japan; 6 Department of Biotechnology, Graduate School of Engineering, Osaka University, Yamadaoka, Suita 565-0871, Japan; 7 Applied Environmental Biology, Graduate School of Pharmaceutical Sciences, Osaka University, Yamadaoka, Suita 565-0871, Japan; 8 Oxford Centre for Diabetes, Endocrinology and Metabolism, University of Oxford, Churchill Hospital, Oxford OX3 7LJ, UK; 9 Alberta Diabetes Institute, University of Alberta, Faculty of Medicine & Dentistry, Edmonton, AB T6G 2E1, Canada; 10 Department of Neural Regeneration and Cell Communication, Mie University Graduate School of Medicine, Edobashi, Tsu 514-8507, Japan; 11 Life Science Research Center, Technology Research Laboratory, Shimadzu Corporation, Soraku-gun, Kyoto 619-0237, Japan

## Abstract

Incretins, hormones released by the gut after meal ingestion, are essential for maintaining systemic glucose homeostasis by stimulating insulin secretion. The effect of incretins on insulin secretion occurs only at elevated glucose concentrations and is mediated by cAMP signaling, but the mechanism linking glucose metabolism and cAMP action in insulin secretion is unknown. We show here, using a metabolomics-based approach, that cytosolic glutamate derived from the malate-aspartate shuttle upon glucose stimulation underlies the stimulatory effect of incretins and that glutamate uptake into insulin granules mediated by cAMP/PKA signaling amplifies insulin release. Glutamate production is diminished in an incretin-unresponsive, insulin-secreting β cell line and pancreatic islets of animal models of human diabetes and obesity. Conversely, a membrane-permeable glutamate precursor restores amplification of insulin secretion in these models. Thus, cytosolic glutamate represents the elusive link between glucose metabolism and cAMP action in incretin-induced insulin secretion.

## Highlights

• Glutamate is derived from the malate-aspartate shuttle upon glucose stimulation
• Shuttle-derived glutamate is crucial for incretin-induced insulin secretion
• Cytosolic glutamate is transported into insulin granules via cAMP/PKA signaling
• Glutamate production by glucose is defective in incretin-unresponsive β cells

## Graphical Abstract

**Figure:**
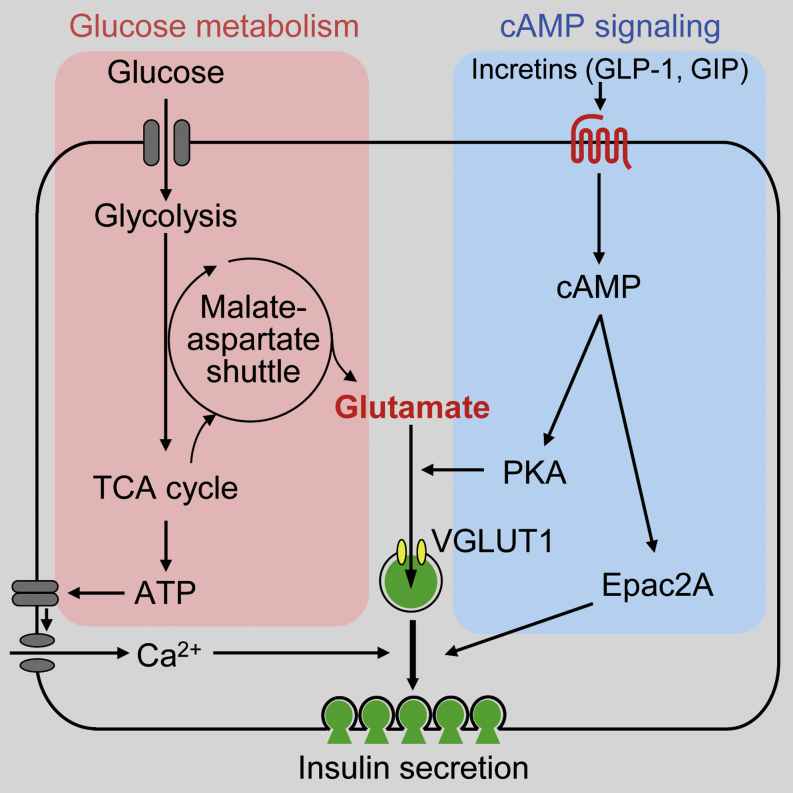

## Teaser

Gheni et al. find that cytosolic glutamate derived from glucose through the malate-aspartate shuttle is the signal underlying incretin-induced insulin secretion. Glutamate uptake into insulin granules mediated by cAMP/PKA signaling amplifies insulin release. Thus, cytosolic glutamate acts as a signal linking glucose metabolism to incretin/cAMP action to amplify insulin secretion.

## Introduction

Insulin secretion from pancreatic β cells is precisely regulated by various intracellular signals to maintain blood glucose levels within a normal range. Impaired insulin secretion contributes to the pathogenesis and pathophysiology of diabetes (Polonsky et al., 1988; Porte, 1991) and is a target for its treatment. According to the consensus model of glucose-induced insulin secretion (GIIS), GIIS depends on a series of carefully orchestrated β cell responses: mitochondrially generated ATP results in closure of ATP-sensitive K^+^ (K_ATP_) channels, which in turn triggers membrane depolarization, electrical activity, and opening of voltage-dependent Ca^2+^ channels (VDCCs), with the resultant elevation of [Ca^2+^]_i_ initiating Ca^2+^-induced insulin granule exocytosis (Henquin, 2000). Thus, ATP produced by glucose metabolism is a critical signal in GIIS.

Pancreatic β cells are equipped with two highly active NADH shuttles linked to glycolysis: the malate-aspartate shuttle and the glycerol phosphate shuttle, both of which contribute to ATP production. Whereas inhibition of either one of the NADH shuttles does not affect GIIS, inhibition of both shuttles abolishes GIIS (Eto et al., 1999). In addition, other intracellular signals in pancreatic β cells, including cAMP and phospholipid-derived molecules such as inositol 1,4,5-triphosphate (IP3) and diacylglycerol (DAG), which are evoked by various nutrients and hormonal and neuronal inputs, exert important modulatory functions of insulin secretion in the maintenance of systemic glucose homeostasis.

Incretins such as glucagon-like peptide 1 (GLP-1) and glucose-dependent insulinotropic polypeptide (GIP) are secreted by the enteroendocrine L cells and K cells, respectively, in response to meal ingestion (Cataland et al., 1974; Kreymann et al., 1987) and are critical for preventing postprandial hyperglycemia by amplifying insulin secretion through cAMP signaling (Drucker, 2006; Holst, 2007). It is well known that incretin/cAMP signaling stimulates insulin secretion in a glucose-dependent manner (Siegel and Creutzfeldt, 1985; Prentki and Matschinsky, 1987; Weir et al., 1989). Importantly, type 2 diabetes is associated with impaired incretin-induced insulin secretion (Nauck et al., 1993; Seino et al., 2010). The identification of the amplifying effect of incretins in insulin secretion has paved the way for recently developed incretin-based diabetes therapies that carry less risk for hypoglycemia (Ahrén, 2009; Drucker and Nauck, 2006).

Recent studies have shown that incretin/cAMP signaling in insulin secretion involves both protein kinase A (PKA)- and Epac2A-dependent pathways (Seino and Shibasaki, 2005). PKA phosphorylates various proteins associated with the insulin secretory process, such as Snapin (Song et al., 2011), MyRIP, Rabphilin (Brozzi et al., 2012), and Rip11 (Sugawara et al., 2009). On the other hand, Epac2A, which contains a guanine nucleotide exchange factor domain, activates the small G-proteins Rap1 and Rap2 upon cAMP binding (Bos, 2006). Epac2A/Rap1 signaling plays a key role in incretin-induced insulin secretion, likely by promoting recruitment of insulin granules and/or fusion events of the granules to the plasma membrane (Shibasaki et al., 2007; Seino et al., 2011) or granule fusion itself (Eliasson et al., 2003).

Glucose metabolism in pancreatic β cells is essential for both triggering insulin secretion by glucose and amplifying insulin secretion by incretin/cAMP signaling, but the mechanism of the link between glucose metabolism and incretin/cAMP action in insulin secretion has not been elucidated. Here, we employed a differential metabolomics-based approach to address this issue using incretin-responsive and -unresponsive β cell lines. We find that cytosolic glutamate derived from the malate-aspartate shuttle upon glucose stimulation is transported into insulin granules by cAMP/PKA signaling, which leads to amplification of insulin granule exocytosis. Our data highlight the role of cytosolic glutamate as a key signal linking glucose metabolism to incretin/cAMP action to amplify insulin secretion.

## Results

### Profiles of Glucose Metabolism Differ between Incretin-Responsive and -Unresponsive Mouse Pancreatic β Cell Lines

**Figure 1:**
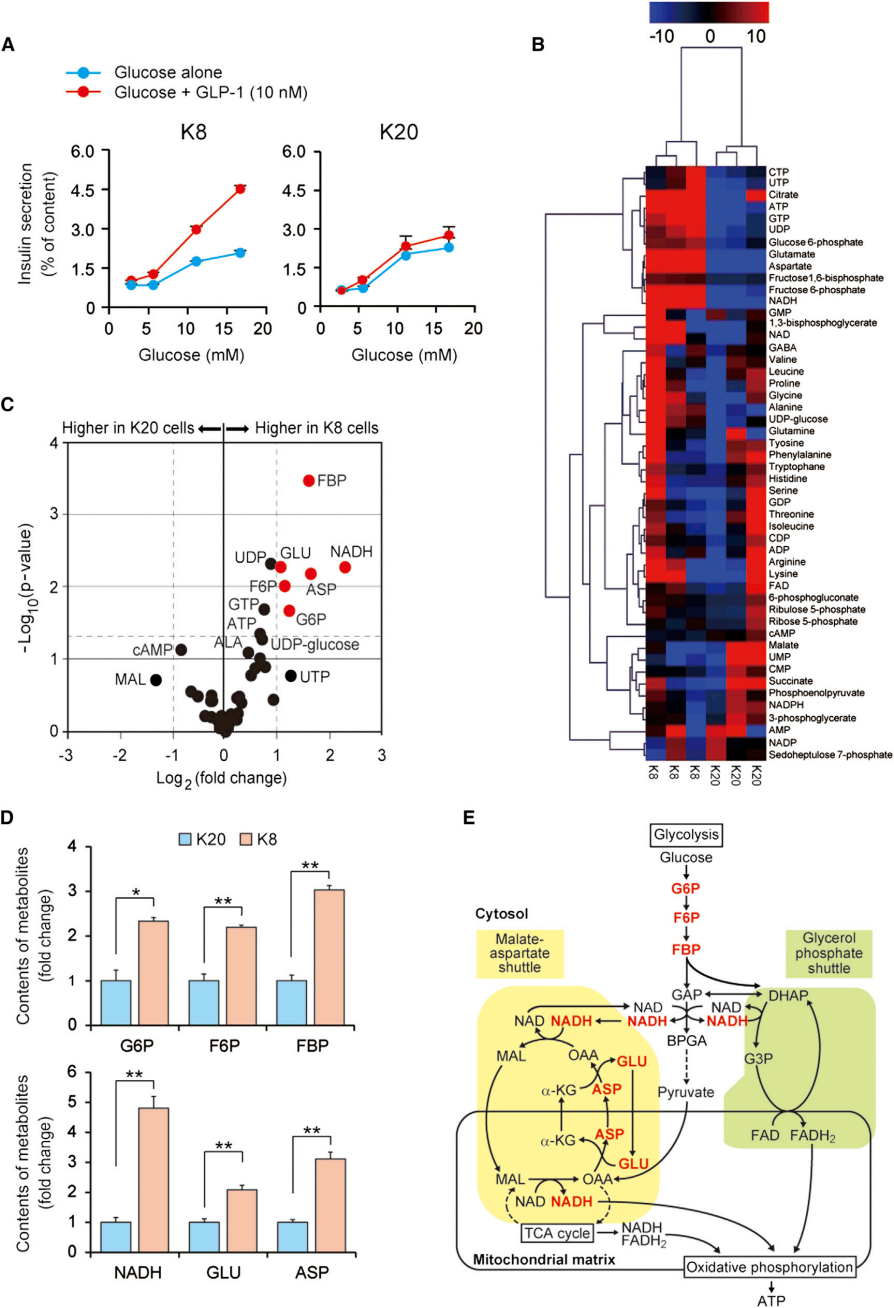
Distinct Profiles of Glucose Metabolism in Incretin-Responsive (MIN6-K8) and -Unresponsive (MIN6-K20) Cells (A) Insulin secretory responses to glucose alone and glucose plus GLP-1 in MIN6-K8 (left) and MIN6-K20 (right) cells (n = 5–8 for each point). (B) Metabolomic profiles expressed as a heatmap in MIN6-K8 and MIN6-K20 cells under the glucose (16.7 mM)-stimulated condition (n = 3 for each). (C) Univariate analysis of metabolome data on MIN6-K8 and MIN6-K20 cells under the glucose (16.7 mM)-stimulated condition (n = 3 for each). Welch’s t test p values and fold changes are shown as a volcano plot. Each dot indicates a metabolite. Metabolites showing a >2-fold [∣Log_2_ (fold change)∣ > 1)] and statistically significant [p < 0.05: -Log_10_ (p value) > 1.3] difference between the two cell lines are indicated in red. ALA, alanine; ASP, aspartate; F6P, fructose 6-phophate; FBP, fructose 1,6-bisphosphate; G6P, glucose 6-phosphate; GLU, glutamate; MAL, malate. (D) Contents of metabolites showing the difference between MIN6-K8 and MIN6-K20 cells under the glucose (16.7 mM)-stimulated condition (n = 3 for each). See also the legend to (C). (E) Schematic view of how the two NADH shuttles (malate-aspartate shuttle and glycerol phosphate shuttle) are linked to glycolysis. Metabolites showing a difference between MIN6-K8 and MIN6-K20 cells are indicated in red. α-KG, α-ketoglutarate; ASP, aspartate; BPGA, 1,3-bisphosphoglycerate; DHAP, dihydroxyacetone phosphate; F6P, fructose 6-phophate; FBP, fructose 1,6-bisphosphate; G3P, glycerol 3-phosphate; G6P, glucose 6-phosphate; GAP, glyceraldehyde 3-phosphate; GLU, glutamate; MAL, malate; OAA, oxaloacetate. See also the legend to (C). The data are expressed as means ± SEM. Results are representative of three independent experiments. Welch’s t test was used for evaluation of statistical significance (C and D). ^∗^p < 0.05; ^∗∗^p < 0.01. See also Table S1 and Figure S1.

We utilized two recently established β cell lines, designated MIN6-K8 and MIN6-K20 cells (Iwasaki et al., 2010), as incretin-responsive and -unresponsive β cell models, respectively, to investigate the mechanism of incretin-induced insulin secretion. Like primary pancreatic β cells, MIN6-K8 cells secrete insulin in response to both glucose and the incretins GLP-1 and GIP, whereas MIN6-K20 cells respond to glucose, but not to the incretins (Figures 1A, S1A, and S1B). We ascertained the integrity of downstream cAMP signaling targets of cAMP (PKA and Epac2A, as assessed by phosphorylation of cAMP response element-binding protein [CREB] or Rap1 activity, respectively) in both MIN6-K8 and MIN6-K20 cells (Figures S1C and S1D). Likewise, no differences in the capacity for cAMP production in response to GLP-1 or GIP in these cells were detected (Iwasaki et al., 2010). These findings indicate that the difference in incretin responsiveness between MIN6-K8 and MIN6-K20 cells is not due to disruption of the incretin/cAMP signaling pathways. Since incretin-induced insulin secretion is glucose dependent, we considered the possibility that the impaired incretin responsiveness of MIN6-K20 cells results from compromised “metabolism-cAMP coupling.” We addressed this possibility by conducting a metabolome analysis of MIN6-K8 and MIN6-K20 cells stimulated by glucose (16.7 mM) (Table S1). Hierarchical cluster multivariate analysis revealed that MIN6-K8 and MIN6-K20 cells were separated into two distinct clusters, indicating differences in the metabolic response to glucose stimulation (Figure 1B). Univariate analysis (fold change and t test) showed that the contents of glucose 6-phosphate (G6P), fructose 6-phosphate (F6P), fructose 1,6-bisphosphate (FBP), NADH, glutamate (GLU), and aspartate (ASP) were significantly higher in MIN6-K8 cells than in MIN6-K20 cells (Figures 1C and 1D; Table S1). These results suggest higher activity of the malate-aspartate shuttle in incretin-responsive MIN6-K8 cells than in incretin-unresponsive MIN6-K20 cells (Figure 1E).

### Essential Role of the Malate-Aspartate Shuttle in Incretin-Induced Insulin Secretion

**Figure 2:**
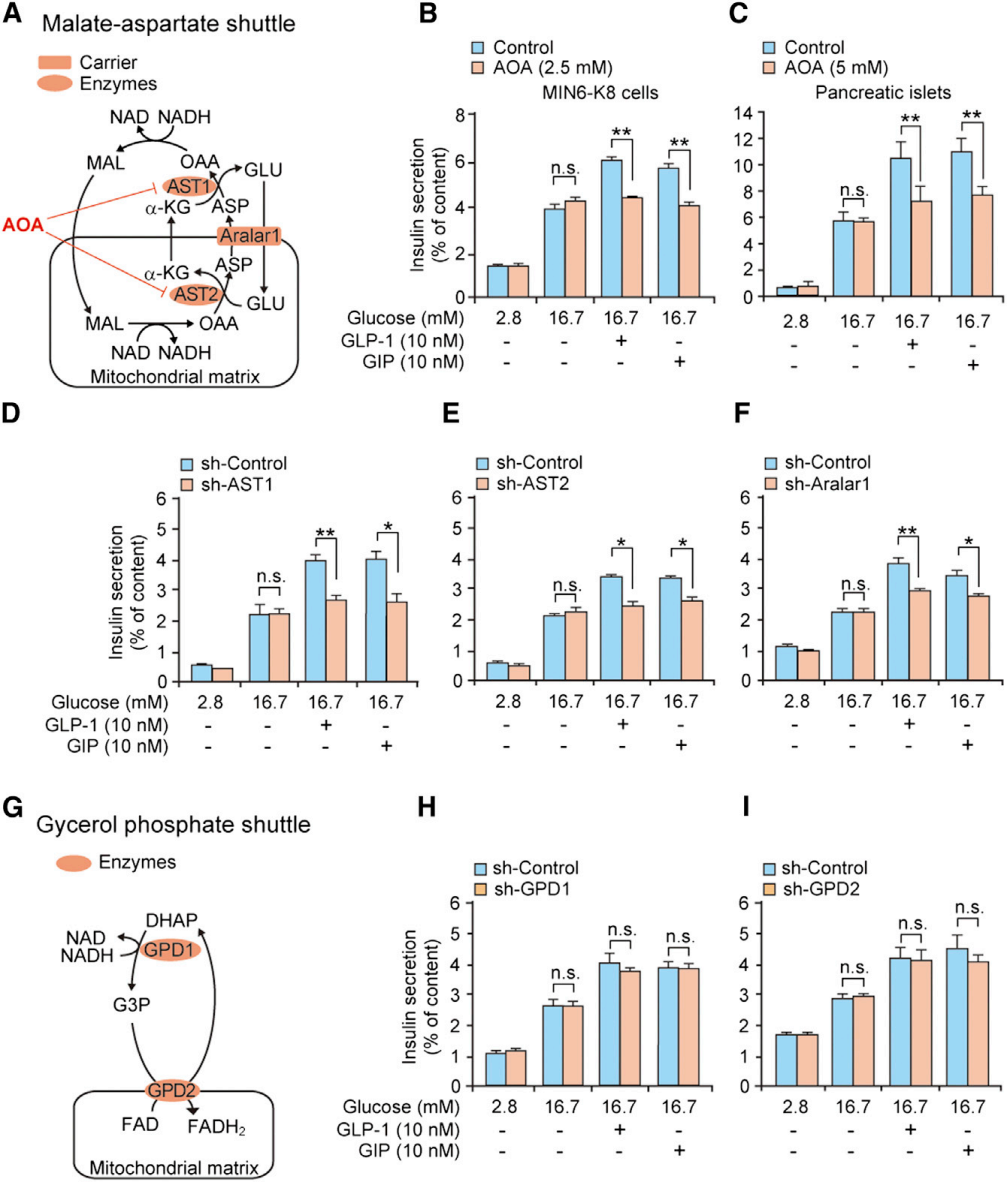
Essential Role of the Malate-Aspartate Shuttle in Incretin-Induced Insulin Secretion (A) Malate-aspartate shuttle. Aralar1, aspartate/glutamate carrier; AST1 and AST2, aspartate aminotransferase 1 and 2, respectively. See also the legend to Figure 1E. (B and C) Effect of AOA, an inhibitor of the malate-aspartate shuttle, on insulin secretion from MIN6-K8 cells (B) and mouse pancreatic islets (C). The concentrations of AOA used were 2.5 mM in MIN6-K8 cells and 5 mM in mouse pancreatic islets. (D–F) Effects of KD of AST1 (D), AST2 (E), and Aralar1 (F) on insulin secretion from MIN6-K8 cells. (G) Glycerol phosphate shuttle. GPD1 and GPD2, glycerol 3-phosphate dehydrogenase 1 and 2, respectively. See also the legend to Figure 1E. (H and I) Effects of KD of GPD1 (H) and GPD2 (I) on insulin secretion from MIN6-K8 cells. The data are expressed as means ± SEM (n = 4–8). Results are representative of three independent experiments. Welch’s t test was used for evaluation of statistical significance. ^∗^p < 0.05; ^∗∗^p < 0.01; n.s., not significant. See also Figure S2.

We then examined the role of the malate-aspartate shuttle in GIIS and incretin-induced insulin secretion (as assessed by amplification of insulin secretion by GLP-1 and GIP) by using aminooxyacetate (AOA), an inhibitor of the shuttle (Eto et al., 1999; MacDonald, 1982; Figure 2A). We found that AOA did not affect GIIS but virtually abolished the response to GLP-1 or GIP in MIN6-K8 cells (Figure 2B). Very similar observations were made in primary mouse pancreatic islets treated with AOA (Figure 2C). These results suggest that whereas activity of the malate-aspartate shuttle is essential for incretin-induced insulin secretion, this is not the case for GIIS. To further confirm the role of the malate-aspartate shuttle, we next examined the effects of knockdown (KD) of the aspartate aminotransferases AST1 and AST2 or the aspartate/glutamate carrier Aralar1 on insulin secretion in MIN6-K8 cells. As illustrated schematically in Figure 2A, these enzymes are all required for malate-aspartate shuttle activity. Reduced expression of AST1 (−68%; Figure S2A), AST2 (−83%; Figure S2B), or Aralar1 (−93%; Figure S2C) did not affect GIIS, but decreased incretin-induced insulin secretion (Figures 2D–2F). By contrast, KD of glycerol 3-phosphate dehydrogenases GPD1 (−52%; Figure S2E) or GPD2 (−80%; Figure S2F), both of which are required for activity of the glycerol phosphate shuttle (another NADH shuttle that is linked to glycolysis; Figure 2G), affected neither GIIS nor incretin-induced secretion (Figures 2H and 2I). The possibility that AOA has effects on other transaminases was ruled out by KD experiments with branched-chain aminotransferase 2 (BCAT2) and alanine aminotransferase 2 (ALT2) (Figures S2H–S2K). We ascertained that KD of these enzymes/carrier did not affect cellular insulin content (Figures S2D, S2G, and S2L). Thus, while GIIS is maintained following abolition of either the malate-aspartate or glycerol phosphate shuttle, incretin-induced insulin secretion depends exclusively on the malate-aspartate shuttle.

### Glucose-Dependent Production of Cytosolic Glutamate and cAMP/PKA-Dependent Glutamate Transport into Insulin Granules

**Figure 3:**
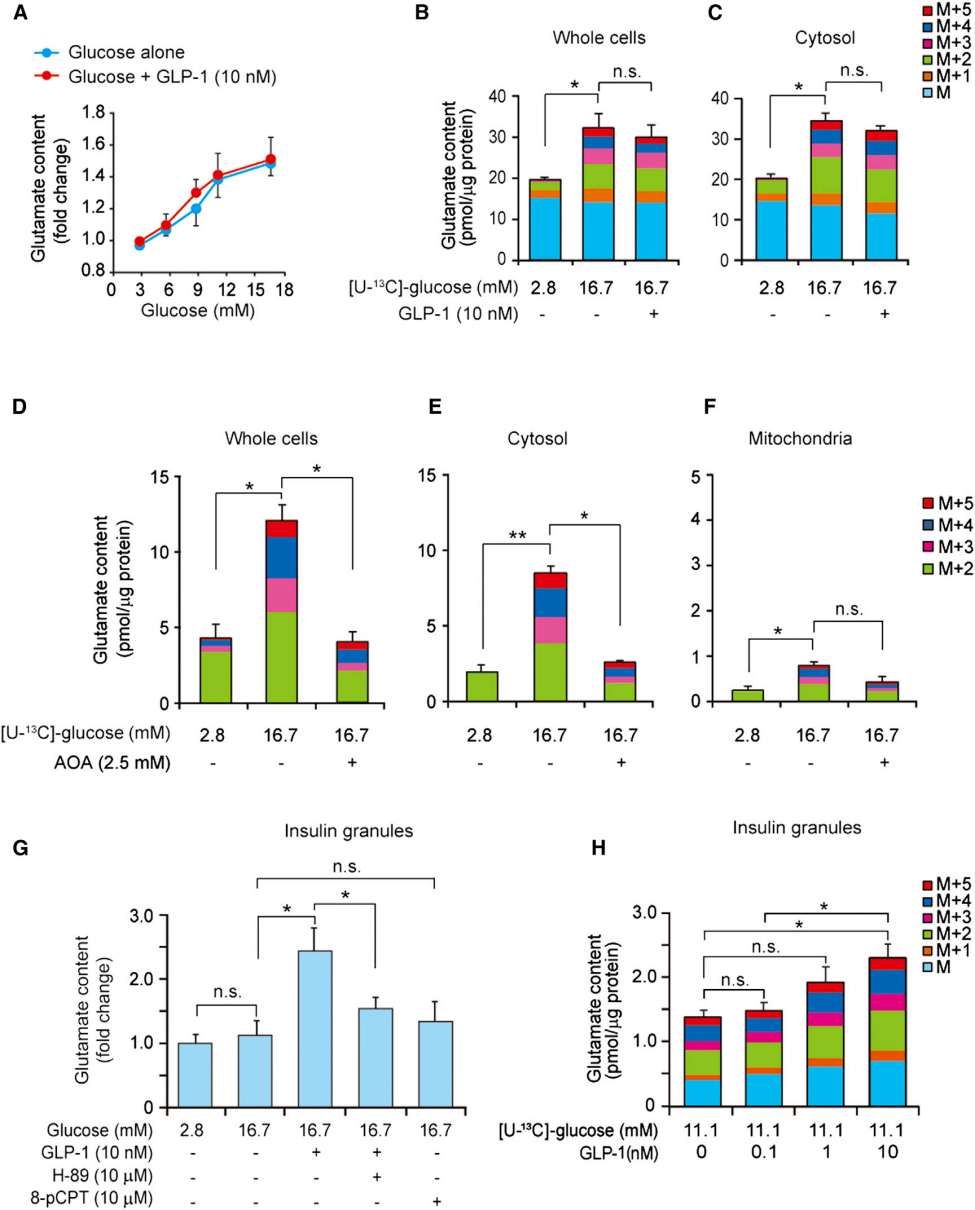
Glucose-Dependent Production of Cytosolic Glutamate and Increased Glutamate Contents in Insulin Granules by cAMP/PKA Signaling (A) Effect of glucose on total cellular glutamate contents in the absence or presence of GLP-1 (10 nM) in MIN6-K8 cells (n = 3 for each point). (B and C) Changes in contents of glutamate isotopomers (M to M+5) by glucose stimulation in the absence or presence of GLP-1 (10 nM) in whole cells (B) and cytosol (C) in MIN6-K8 cells (n = 4–5 for each). (D–F) Effects of AOA on contents of glutamate isotopomers (M+2 to M+5) in whole cells (D), cytosol (E), and mitochondria (F) in MIN6-K8 cells (n = 3 for each). (G) Effects of glucose, GLP-1 (10 nM), H-89 (10 μM, a PKA inhibitor), and 8-pCPT (10 μM, 8-pCPT-2′-*O*-Me-cAMP-AM, an Epac-selective cAMP analog) on glutamate contents in insulin granules in MIN6-K8 cells (n = 4–12). (H) Dose-dependent effects of GLP-1 on glutamate contents in insulin granules in MIN6-K8 cells under the glucose (11.1 mM)-stimulated condition (n = 4 for each). The data are expressed as means ± SEM. Results are representative of three independent experiments. The Tukey-Kramer method was used for evaluation of statistical significance. ^∗^p < 0.05; ^∗∗^p < 0.01; n.s., not significant. See also Table S2 and Figure S3.

We next attempted to clarify the relationship between the malate-aspartate shuttle and incretin stimulation. We first considered direct incretin-induced activation of the shuttle. However, metabolome analysis revealed (with the exception of cAMP; Table S2) no differences in the contents of the metabolites associated with glycolysis and the malate-aspartate shuttle in MIN6-K8 cells stimulated by glucose (16.7 mM) alone or glucose plus GLP-1 (10 nM or 100 nM). In addition, the activities of AST1 and malate dehydrogenase MDH1, both of which are required for malate-aspartate shuttle activity (Figure S3A), were not increased by GLP-1 (Figures S3B and S3C). These results indicate that incretin-induced insulin secretion is not caused by direct activation of the malate-aspartate shuttle. We therefore explored the alternative possibility that a metabolite associated with the malate-aspartate shuttle mediates the effect on insulin secretion. We focused on glutamate since it was increased in response to glucose in incretin-responsive MIN6-K8 cells and was previously proposed to be a signal in insulin secretion (Maechler and Wollheim, 1999). Cytosolic glutamate is converted from α-ketoglutarate through the malate-aspartate shuttle upon glucose stimulation (Figure 2A). We therefore hypothesized that cytosolic glutamate might mediate incretin-induced insulin secretion. Total cellular glutamate content was increased in a glucose-concentration-dependent manner, but was not affected by GLP-1 (Figure 3A). α-Ketoglutarate content also was increased by glucose stimulation (Figure S3D). We next used mass spectrometry to determine ^13^C enrichment of glutamate in MIN6-K8 cells exposed to [U-^13^C]-glucose. The cytosolic contents of M and M+1 glutamate isotopomers (no substitution with ^13^C derived from [U-^13^C]-glucose), both of which are naturally existing in cells, were unchanged by glucose stimulation, whereas M+2, M+3, M+4, and M+5 glutamate isotopomers (two to five ^13^C substitutions for ^12^C) were increased significantly (Figures 3B and 3C). GLP-1 did not alter the distribution of glutamate isotopomers produced by glucose.

We then investigated the involvement of the malate-aspartate shuttle in glutamate production using preparations of whole cells and cytosolic and mitochondrial fractions of MIN6-K8 cells with or without AOA treatment. We found that cytosolic glutamate contributed to the majority of cellular glutamate under glucose stimulation and that treatment with AOA markedly suppressed the production of both cellular and cytosolic glutamate (Figures 3D–3F and S3E). In addition, neither GIIS nor incretin-induced insulin secretion was affected by KD of glutamate dehydrogenase 1 (GDH1; −72%; Figures S3F–S3H), the enzyme that catalyzes the production of mitochondrial glutamate from α-ketoglutarate. Together, these results suggest that glucose increases cytosolic glutamate through the malate-aspartate shuttle.

Importantly, glutamate content in insulin granules was not increased by glucose (16.7 mM) alone, but was increased significantly by the addition of GLP-1 (Figures 3G and S3I). The increase in granular glutamate content by GLP-1 was blocked by H-89, a PKA inhibitor, whereas the glutamate content was not increased by 8-pCPT-2′-*O*-Me-cAMP-AM, an Epac-selective cAMP analog (Figure 3G), indicating that glutamate content in insulin granules is increased by a cAMP/PKA-dependent mechanism. By analyzing ^13^C-enriched glutamate using [U-^13^C]-glucose as a substrate, we confirmed that GLP-1 increased the amounts of M+2 to M+5 glutamate isotopomers (two to five ^13^C substitutions for ^12^C) in insulin granules in a concentration-dependent manner (Figure 3H), a finding that is consistent with the effect of GLP-1 on insulin secretion (Figure S3J). These results suggest that cytosolic glutamate derived from α-ketoglutarate through the malate-aspartate shuttle represents a signal that mediates incretin-induced insulin secretion.

### Glutamate as a Signal in Incretin-Induced Insulin Secretion

**Figure 4:**
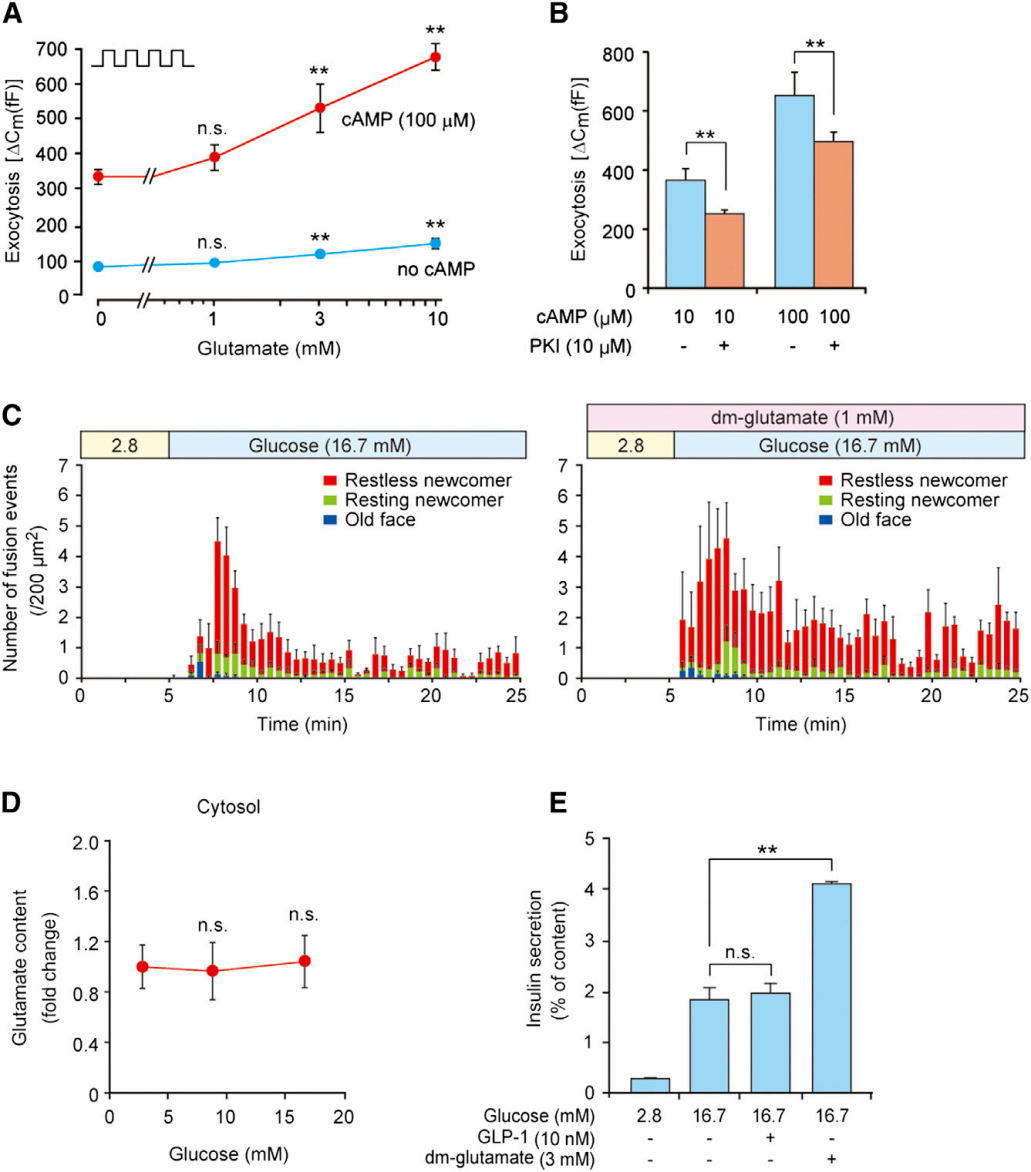
Glutamate as a Signal in Incretin-Induced Insulin Granule Exocytosis (A) Effect of intracellular glutamate on exocytosis. Exocytosis (ΔC_m_) was measured in a single mouse β cell at concentrations of 0, 1, 3, and 10 mM intracellular glutamate (added via the recording electrode) in the presence or absence of 100 μM cAMP (n = 19–31 for each point). Exocytosis was elicited by trains of four 500 ms depolarizations from −70 mV to zero mV applied at 1 Hz (indicated schematically in the upper-left corner). All data points in the presence of cAMP are significantly different from corresponding values in the absence of cAMP (p < 0.05 or better). Glutamate (3 and 10 mM) stimulates exocytosis in both the absence and presence of cAMP as compared with the relative control (no glutamate), but responses in the absence of cAMP are dwarfed compared with the much larger effects in the presence of cAMP. ^∗∗^p < 0.01 versus no glutamate in the absence or presence of cAMP. (B) Effect of PKI (10 μM), a PKA-inhibitory peptide, on exocytosis. Exocytosis (ΔC_m_) was measured in a single mouse β cell in the presence of 3 mM glutamate in cells exposed to 10 and 100 μM cAMP as indicated (n = 20–27). (C) Effect of dimethyl-glutamate (dm-glutamate), a membrane-permeable glutamate precursor, on insulin granule exocytosis. The exocytosis was measured as fusion events by TIRFM. Histograms show the number of fusion events caused by glucose alone (left) and glucose plus dimethyl-glutamate (right) in primary cultured mouse pancreatic β cells (n = 4 for each); “2.8” indicates 2.8 mM glucose. (D) Effect of glucose on cytosolic glutamate production in incretin-unresponsive MIN6-K20 cells (n = 7–8 for each point). (E) Effect of dimethyl-glutamate (dm-glutamate) on insulin secretion from incretin-unresponsive MIN6-K20 cells (n = 4–8 for each). The data are expressed as means ± SEM. Results are representative of three independent experiments. Student’s t test (A and B) and Dunnett’s method (D and E) were used for evaluation of statistical significance. ^∗∗^p < 0.01; n.s., not significant. See also Figure S4.

To clarify whether glutamate acts as a signal in incretin-induced insulin secretion, we next investigated the role of glutamate in cAMP-induced insulin granule exocytosis. We examined the effect of increasing concentrations of cytosolic glutamate on depolarization-evoked exocytosis in pancreatic β cells using the standard whole-cell technique in conjunction with measurements of membrane capacitance (ΔC_m_) (Rorsman and Renström, 2003) in the absence and presence of cAMP. Whereas glutamate (3 and 10 mM) stimulated exocytosis in the absence of cAMP, consistent with previous reports (Høy et al., 2002), the effect was minute compared with the much stronger amplification seen in the presence of cAMP (Figure 4A). The effect of glutamate on exocytosis in the presence of 100 μM cAMP was not mimicked by malate (10 mM; data not shown). Exocytosis evoked by glutamate in the presence of cAMP was inhibited by application of PKI (10 μM), a PKA-inhibitory peptide (Figure 4B). Together with the finding that the increase in glutamate content in insulin granules by GLP-1 was blocked by H-89 (Figure 3G), these results indicate that glutamate acts as a signal in cAMP-induced exocytosis in a PKA-dependent manner.

To further confirm that glutamate acts as an amplifying signal in insulin secretion, we examined the effect of dimethyl-glutamate, a membrane-permeable glutamate precursor (Maechler and Wollheim, 1999). We found that dimethyl-glutamate is converted to glutamate in insulin granules as well as in the cytosol (Figures S4A and S4B), as assessed by analysis of ^13^C-enriched glutamate in MIN6-K8 cells. Dimethyl-glutamate amplified both the first and second phases of glucose-induced insulin granule exocytosis (analyzed by total internal reflection fluorescence microscopy [TIRFM]) (Shibasaki et al., 2007; Figures 4C and S4C). In incretin-unresponsive MIN6-K20 cells, cytosolic glutamate content was not increased at all by glucose (Figure 4D), but dimethyl-glutamate markedly amplified insulin secretion (Figure 4E). As dimethyl-malate and dimethyl-succinate have no effects on insulin secretion (Figures S4D and S4E), it is unlikely that dimethyl-glutamate is used as a fuel to stimulate insulin secretion. These results indicate that dimethyl-glutamate mimics the effect of incretin/cAMP on insulin secretion. Collectively, these findings corroborate the view that glutamate acts as an amplifying signal in incretin-induced insulin secretion.

### Requirement of Glutamate Transport into Insulin Granules for Amplification of Insulin Secretion by Incretin/cAMP Signaling

**Figure 5:**
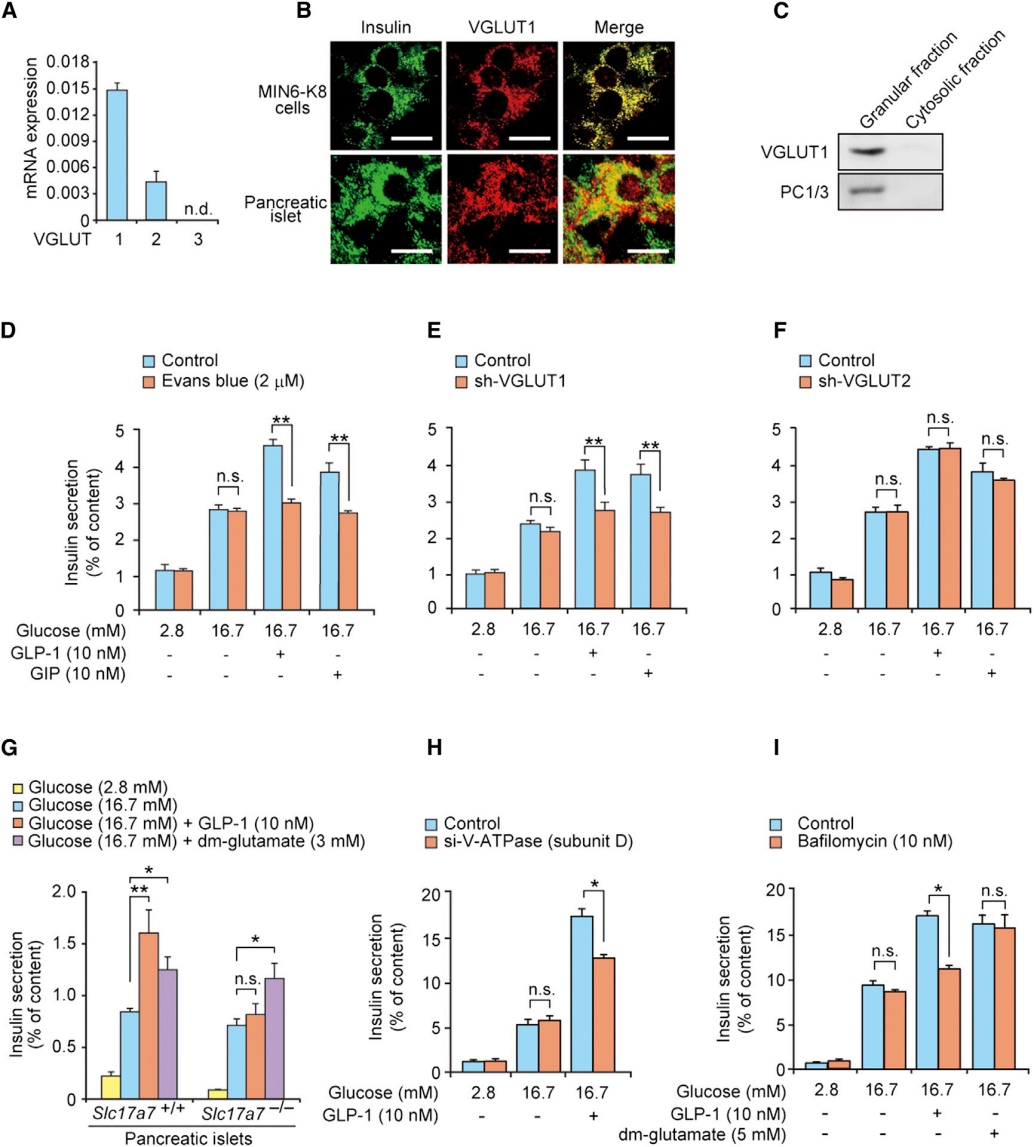
Requirement of Glutamate Transport into Insulin Granules for Amplification of Insulin Secretion by Incretin/cAMP Signaling (A) mRNA expression levels of VGLUTs in MIN6-K8 cells (n = 3–4 for each). n.d., not detected. (B) Immunocytochemical analysis of VGLUT1 in MIN6-K8 cells and pancreatic islets. Scale bars, 10 μm. (C) Immunoblot analysis of VGLUT1 in insulin granules in MIN6-K8 cells. The insulin granule fraction was confirmed by immunoblot analysis using anti-PC1/3 antibody. (D) Effect of Evans blue, an inhibitor of vesicular glutamate transport, on insulin secretion from MIN6-K8 cells (n = 5–8 for each). (E and F) Effects of KD of VGLUT1 (E) and VGLUT2 (F) on insulin secretion from MIN6-K8 cells (n = 4–8 for each). (G) Insulin secretion from pancreatic islets of wild-type (*Slc17a7*^+/+^) and VGLUT1 knockout (*Slc17a7*^−/−^) mice (n = 4–8 for each). dm-glutamate, dimethyl-glutamate. (H) Effect of KD of V-ATPase subunit D on insulin secretion from MIN6-K8 cells (n = 4 for each). (I) Effect of bafilomycin, an inhibitor of V-ATPase, on insulin secretion from MIN6-K8 cells (n = 4 for each). The data are expressed as means ± SEM. Results are representative of three independent experiments. Welch’s t test (D–F, H, and I) and Dunnett’s method (G) were used for evaluation of statistical significance. ^∗^p < 0.05; ^∗∗^p < 0.01; n.s., not significant. See also Figure S5.

Glutamate is transported into secretory vesicles in neurons (Naito and Ueda, 1983) and enteroendocrine cells (Uehara et al., 2006) through vesicular glutamate transporters (VGLUTs) (Bellocchio et al., 2000; Gras et al., 2002; Omote et al., 2011; Takamori et al., 2001). VGLUT1, VGLUT2, and VGLUT3 are expressed in the pancreatic β cell lines βTC6, RINm5F, and INS-1E, and their insulin granules have the capacity to accumulate glutamate (Bai et al., 2003; Gammelsaeter et al., 2011). Analysis by quantitative real-time RT-PCR showed that VGLUT1 is the predominant VGLUT in MIN6-K8 cells (Figure 5A). Immunocytochemical and immunoblot analyses revealed that VGLUT1 colocalizes with insulin granules (Figures 5B and 5C). We then examined the role of glutamate transport into insulin granules in insulin secretion using Evans blue, an inhibitor of glutamate transport into secretory vesicles (Maechler and Wollheim, 1999; Roseth et al., 1995), and KD of VGLUT1 (−94%; Figure S5A). The two procedures yielded identical responses: GIIS was not affected, but the incretin-induced stimulation was abolished (Figures 5D and 5E). By contrast, KD of VGLUT2 (−71%; Figure S5B) affected neither GIIS nor incretin-induced insulin secretion (Figure 5F), and KD of either VGLUT did not affect cellular insulin content (Figures S5C and S5D). Dynamic measurements of insulin secretion demonstrated that the amplification by GLP-1 of both the first and second phases of insulin secretion in MIN6-K8 cells was strongly inhibited by KD of VGLUT1 (Figure S5E). We also examined insulin secretion from the pancreatic islets of VGLUT1 knockout (*Slc17a7*^−/−^) mice. GIIS did not differ between wild-type (*Slc17a7*^+/+^) and *Slc17a7*^−/−^ islets, but the stimulatory effect of GLP-1 was not seen in *Slc17a7*^−/−^ islets (Figure 5G). Importantly, dimethyl-glutamate restored amplification of insulin secretion in *Slc17a7*^−/−^ mice (Figure 5G).

We next examined the potential role of V-ATPase, which has been shown to participate in vesicular glutamate transport (Omote et al., 2011), in insulin secretion in MIN6-K8 cells. Neither KD of V-ATPase subunit D (−69%; Figure S5F) nor bafilomycin, an inhibitor of V-ATPase, affected GIIS, but both reduced the amplification evoked by GLP-1 (Figures 5H and 5I) without affecting cellular insulin content (Figure S5G). Intriguingly, dimethyl-glutamate restored insulin secretion in the bafilomycin-treated MIN6-K8 cells (Figure 5I). These results indicate that glutamate transport into insulin granules through VGLUT1 is required for incretin-induced insulin secretion.

### Pathophysiological Role of Glutamate in Insulin Secretion

**Figure 6:**
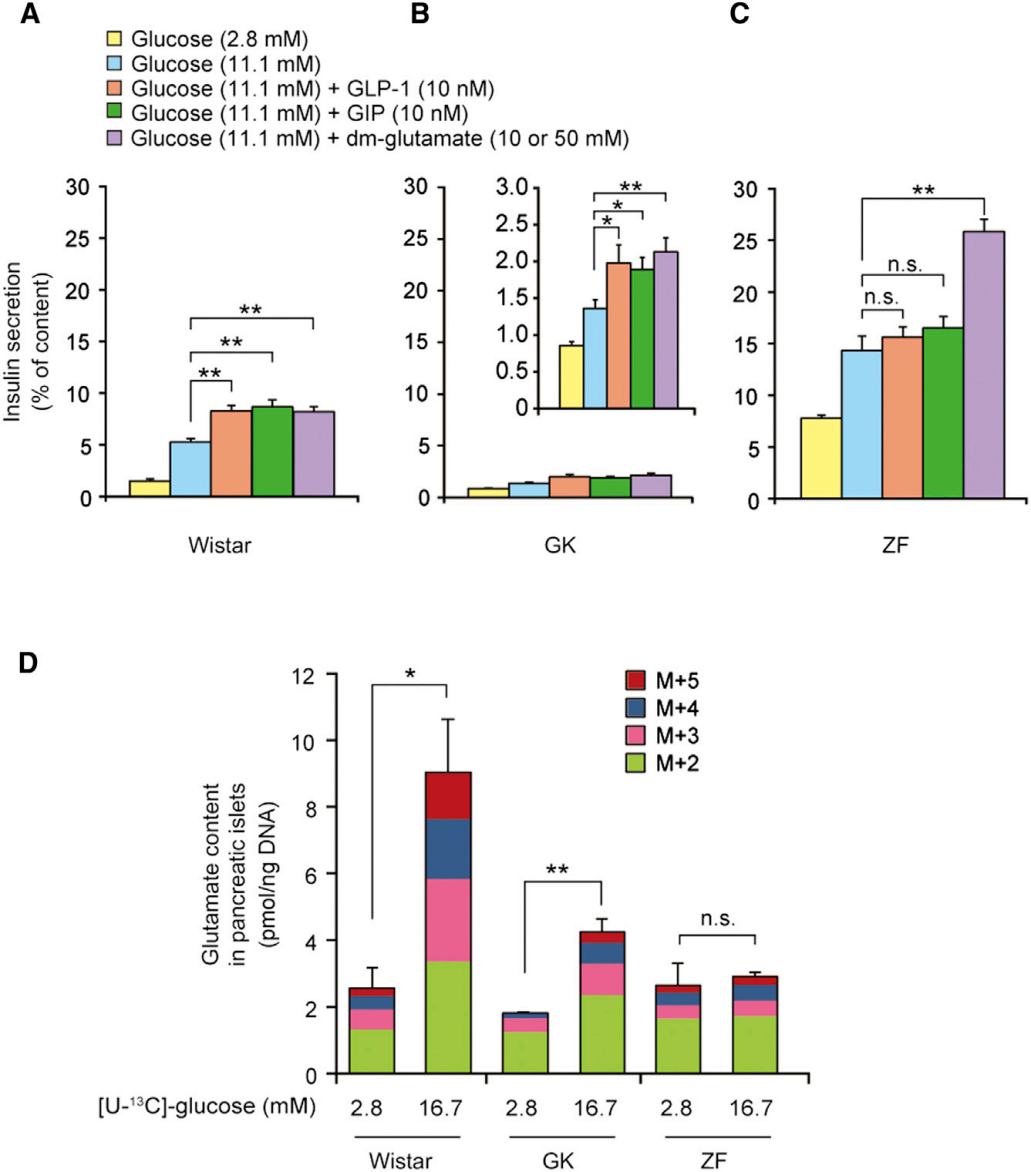
Pathophysiological Role of Glutamate in Insulin Secretion (A–C) Insulin secretion from pancreatic islets of Wistar (A, n = 8 for each), GK (B, n = 6 for each), and ZF (C, n = 8 for each) rats. The inset in the middle panel (B) is included to magnify the scale of the *y* axis in GK rats. The concentrations of dm-glutamate used were 10 mM, 10 mM, and 50 mM for Wistar, GK, and ZF rats, respectively. (D) Production of glutamate isotopomers (M+2 to M+5) by glucose stimulation in pancreatic islets of Wistar, GK, and ZF rats (n = 3 for each). The data are expressed as means ± SEM. Results are representative of three independent experiments. Dunnett’s method (A–C) and Welch’s t test (D) were used for evaluation of statistical significance. ^∗^p < 0.05; ^∗∗^p < 0.01; n.s., not significant. See also Figure S6.

To clarify the relationship between glutamate production and insulin secretion in disease states, we compared GIIS and incretin-induced insulin secretion in nondiabetic Wistar, diabetic Goto-Kakizaki (GK) (Goto et al., 1975), and obese Zucker fatty (ZF) rats (Zucker and Zucker, 1961). The GK rat is a model of nonobese type 2 diabetes with defective insulin secretion associated with impaired glucose metabolism in pancreatic β cells (Ostenson et al., 1993). Although GIIS from the pancreatic islets of GK rats was markedly decreased compared with that of Wistar rats (Figures 6A, 6B, and S6A), the incretins retained their amplifying capacity. Dimethyl-glutamate also amplified insulin secretion in GK rats. In contrast, in ZF rats, a model of obesity with a mutation in the leptin receptor gene, basal insulin secretion was already high compared with the control, but significant GIIS occurred (Figures 6C and S6B). Unlike Wistar and GK rats, adult ZF rats (≥12 weeks of age) exhibited no amplification of insulin secretion in response to incretin stimulation, but dimethyl-glutamate remained effective. We then examined whether glucose-induced islet glutamate production is affected in these rat models, as assessed by analysis of ^13^C-enriched glutamate. Glucose stimulated glutamate production in GK islets, but the levels were lower than those in Wistar islets (Figure 6D). By contrast, no glucose-stimulated glutamate production was seen in ZF islets.

## Discussion

**Figure 7:**
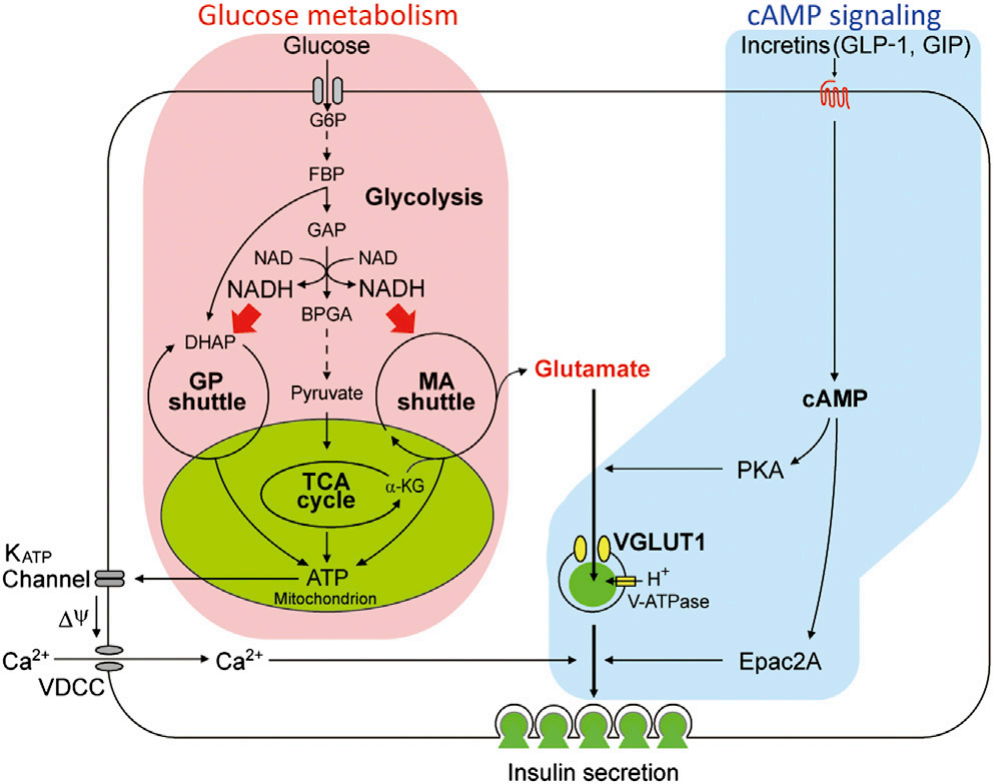
Glutamate Acts as a Key Signal in Amplification of Insulin Secretion by Incretin/cAMP Signaling Glutamate links glucose metabolism and incretin/cAMP signaling to amplify insulin secretion. See text for the details. GP shuttle, glycerol phosphate shuttle; MA shuttle, malate-aspartate shuttle; K_ATP_ channel, ATP-sensitive K^+^ channel; VDCC, voltage-dependent Ca^2+^ channel.

Using metabolome analysis by mass spectrometry, we found that glucose increased cytosolic glutamate through the malate-aspartate shuttle and that GLP-1 promoted glutamate transport into insulin granules via cAMP/PKA signaling. We also found that glutamate in insulin granules stimulated insulin secretion, as assessed by capacitance measurements, TIRFM analysis, and VGLUT1 knockout and KD experiments. Glutamate thus acts as a key cell signal linking glucose metabolism to incretin/cAMP action to amplify insulin secretion. Figure 7 summarizes these findings schematically.

It is well established that incretins and agents that elevate intracellular cAMP amplify insulin secretion but are unable to initiate insulin secretion on their own. The amplifying effect is not mediated by enhanced glucose metabolism, i.e., increased ATP production (Brisson et al., 1972; Peyot et al., 2009), and the nature of the molecular link between glucose metabolism and incretin/cAMP signaling in amplification of insulin secretion remains elusive. To clarify this, we performed a metabolome analysis using capillary electrophoresis-mass spectrometry (CE-MS), which can detect and quantify metabolites, including the intermediate metabolites in glycolysis and the TCA cycle, amino acids, and nucleic acids. Based on our findings that GLP-1 has little or no effect on the amounts of metabolites in glucose metabolism (glycolysis, TCA cycle, pentose phosphate pathway, glycogenesis, and NADH shuttles) or the activities of enzymes in the malate-aspartate shuttle, we can discard the possibility that incretin/cAMP signaling exerts a direct effect on glucose metabolism.

Using differential metabolomics, we compared metabolites associated with glucose metabolism between incretin-responsive and -unresponsive β cell lines. Based on the finding that inhibition of the malate-aspartate shuttle blocked incretin-induced insulin secretion, we focused on the metabolites derived from the shuttle. Among them, glutamate attracted our attention. Mitochondrial glutamate (produced by GDH) was previously proposed to act as a signal in GIIS (Maechler and Wollheim, 1999; Høy et al., 2002; Casimir et al., 2009), although this has been controversial and some observations seem inconsistent with the notion (Bertrand et al., 2002; MacDonald and Fahien, 2000). In the present study, we found that 72% KD of GDH1 (at the mRNA level) did not affect GIIS in MIN6-K8 cells. However, we cannot rule out the possibility that mitochondrial glutamate is involved in GIIS, since knockout of GDH1 in mice decreased GIIS by ∼50% compared with control (Carobbio et al., 2009; Vetterli et al., 2012). On the other hand, the role of cellular glutamate in amplification of insulin secretion by incretin/cAMP signaling has not been investigated. We demonstrate here that cytosolic glutamate derived from the malate-aspartate shuttle, which represents a major fraction of cellular glutamate produced by glucose, is crucial for incretin-induced insulin secretion. Mitochondrial glutamate may also contribute to a fraction of cytosolic glutamate, as it is continuously exported to cytosol through GC1 (Casimir et al., 2009).

Our findings indicate that glutamate transport into the insulin granules occurs only in cells exposed to the combination of glucose and GLP-1, and not in cells exposed to glucose alone. Indeed, inhibition of glutamate transport into the granules diminished incretin-induced insulin secretion, but did not affect GIIS. Our data demonstrate that glutamate transport into insulin granules by cAMP signaling plays a decisive role in incretin-induced insulin secretion. Although glucose induces cAMP production (Charles et al., 1973; Grill and Cerasi, 1973; Dyachok et al., 2008), it produces a much smaller amount of cAMP than does GLP-1 (Delmeire et al., 2003; Iwasaki et al., 2010; Susini et al., 1998). Thus, glucose-induced cAMP production is insufficient (at least under the experimental conditions used in the present study) to sustain sufficient glutamate transport into the insulin granules, but under other experimental paradigms, this could occur.

In neurons, glutamate transport into the synaptic vesicles through VGLUTs is regulated by the electrical potential (Δφ) and pH gradient (ΔpΗ) across the vesicular membrane (Omote et al., 2011). A proton pump V-ATPase on insulin granules contributes to generation of Δφ and ΔpΗ across the insulin granule membrane (Aspinwall et al., 1997), in which Cl^−^ flux is needed (Xie et al., 1983; Xie et al., 1989). The Cl^−^ flux through the chloride transport protein ClC-3 in the insulin granule membrane has been shown to be required for GIIS (Li et al., 2009). In addition, it was reported that glutamate flux in the insulin granules was involved in the generation of Δφ and ΔpΗ, and that perturbing the flux decreased insulin granule exocytosis (Gammelsaeter et al., 2011). We found that inhibition of either VGLUT1 or V-ATPase did not affect GIIS, but reduced amplification of insulin secretion by incretins. Furthermore, dimethyl-glutamate was able to mimic the effects of incretins under the condition of VGLUT1 deficiency or V-ATPase inhibition. These results indicate that glutamate transport into insulin granules, which is mediated by activation of cAMP/PKA signaling, is required for amplification of insulin granule exocytosis. Although the precise mechanism by which glutamate in insulin granules stimulates insulin granule exocytosis remains to be elucidated, the finding by TIRFM analysis that most of the insulin granule exocytosis induced by dimethyl-glutamate is caused by granules that are newly recruited and immediately fused to the plasma membrane (Shibasaki et al., 2007) suggests that glutamate in insulin granules facilitates recruitment toward and/or fusion of the insulin granules with the plasma membrane.

We also provide data implicating defective glutamate signaling in the pathophysiology of insulin secretion in the GK and ZF rat models of human diabetes and obesity, respectively. In GK rats, amplification of insulin secretion by incretins was much reduced, with a marked suppression of glutamate production by glucose. In ZF rats, there was no amplification at all by incretins, with no glutamate production. Importantly, dimethyl-glutamate was able to amplify insulin secretion, mimicking the effect of incretins, in all of these models. Thus, impaired production of glutamate in pancreatic β cells could lead to a defect in incretin-induced insulin secretion. Indeed, some reports have suggested that incretin-based therapies have limited insulinotropic effects in clinical settings (Holst et al., 2011; Kubota et al., 2012).

In summary, our data demonstrate that glutamate acts as a key signal linking glucose metabolism and incretin/cAMP action to amplify insulin secretion. Therefore, elucidating glutamate signaling may not only clarify the pathophysiology of diabetes mellitus but may also pave the way for novel therapeutic strategies.

## Experimental Procedures

### Animal Experiments

Male Wistar (Slc:Wistar), GK (GK/Slc), and ZF (Slc:Zucker, *fa*/*fa*) rats (10 weeks old) were purchased from Japan SLC. VGLUT1 knockout (*Slc17a7*^−/−^) mice were provided by R.H. Edwards. Animal experiments were approved by the Committee on Animal Experimentation of Kobe University and carried out in accordance with the Guidelines for Animal Experimentation at Kobe University. Electrophysiological experiments were performed on β cells from NMRI mice and the experiments were conducted in accordance with the Animals (Scientific Procedures) Act 1986 and ethical guidelines of the University of Alberta and University of Oxford.

### Metabolome Analysis

Hydrophilic metabolites were extracted from MIN6-K cells and then subjected to CE-MS. See Supplemental Experimental Procedures for details.

### Insulin Secretion

Insulin-secretion experiments on MIN6-K cells and pancreatic islets were performed as previously described (Yasuda et al., 2010). See Supplemental Experimental Procedures for details.

### KD Experiments

MIN6-K8 cells were infected with small interfering RNA (siRNA) or adenovirus carrying short hairpin RNA (shRNA). See Supplemental Experimental Procedures for details.

### mRNA Expression

Isolation of total RNA and quantitative real-time RT-PCR were performed as previously described (Matsubara et al., 2012), with 18S ribosomal RNA used as an internal control.

### Glutamate Contents

Glutamate contents in lysed cells were determined with the use of the L-Glutamate Assay Kit II (Yamasa). The contents of glutamate isotopomers were also measured by ^13^C-enrichment analysis, with uniformly labeled [U-^13^C]-glucose as a substrate, using CE-MS. See Supplemental Experimental Procedures for details.

### Electrophysiology

Capacitance recordings of single β cell exocytosis were obtained essentially as described previously (Kanno et al., 2004). See Supplemental Experimental Procedures for details.

### Immunofluorescence Staining

Immunofluorescence staining was performed as previously described (Yasuda et al., 2010). See Supplemental Experimental Procedures for details.

### TIRFM Analysis

TIRFM analysis was performed as previously described (Shibasaki et al., 2007).

### Statistical Analysis

The data are expressed as means ± SEM. Statistical comparisons were made using Welch’s t test, Student’s t test, Dunnett’s method, and the Tukey-Kramer method as indicated in the figure legends. Differences for which the p value was <0.05 were regarded as statistically significant. Hierarchical cluster analysis (average linkage) was performed on Pareto-scaled metabolomics data (distance measures based on the Pearson correlation) using Multiple Experiment Viewer (MeV) (Saeed et al., 2003).

## Author Contributions

S.S. conceived the project. G.G., M.O., M.I., N.Y., K.M., T.S., N.I., T.B., A.M., E.F., P.R., and S.S. contributed to the study design and data analyses. G.G., M.O., M.I., N.Y., K.M., Y.N., K.H., B.H., X.W., H.T., K.K., T.M., R.H., N.H., K.S., and A.M. performed the experiments and collected the data. N.Y., K.M., T.S., E.F., P.R., and S.S. supervised the work. All authors participated in discussion of the results. G.G., M.O., M.I., N.Y., and S.S. wrote the manuscript with feedback from all other authors, including significant contributions from K.M., T.S., H.T., and P.R.

## Supplemental Information

Document S1. Supplemental Experimental Procedures, Figures S1–S6, and Tables S1 and S2

Document S2. Article plus Supplemental Information
