# Supplementary material for: Glutamate Acts as a Key Signal Linking Glucose Metabolism to Incretin/cAMP Action to Amplify Insulin Secretion
Source: Cell Rep. 2014 Oct 16;9(2):661–73. doi: 10.1016/j.celrep.2014.09.030 (PMC4536302; doi:10.1016/j.celrep.2014.09.030)
Supplement: Document S1. Supplemental Experimental Procedures, Figures S1–S6, and Tables S1 and S2 [file mmc1.pdf]

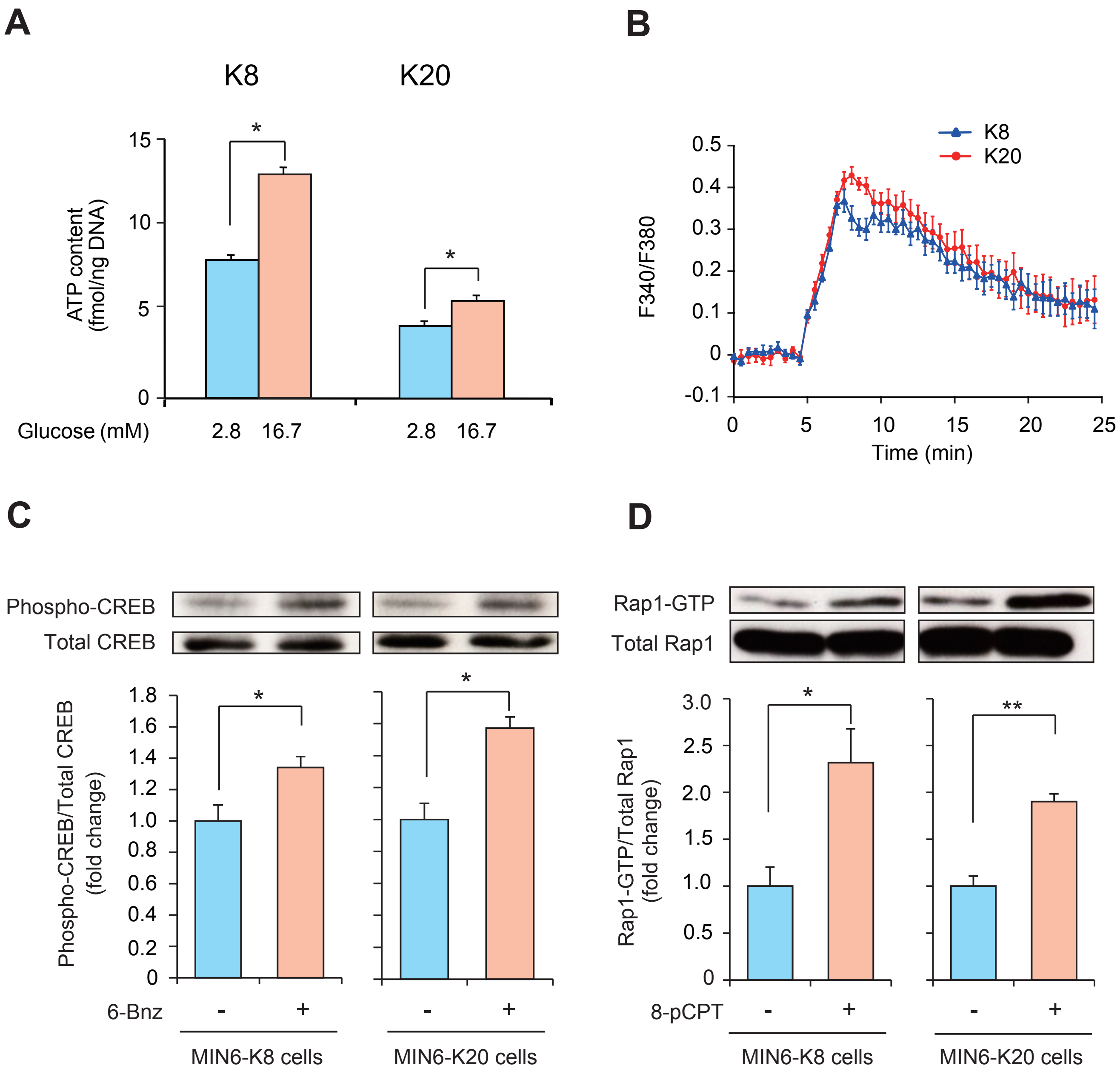

Figure S1. Comparison of ATP Content, Intracellular Ca^{2+} Concentration, and Activities of Downstream Targets of cAMP between MIN6-K8 and-K20 Cells, related to Figure 1

(A) ATP contents in MIN6-cells treated with glucose at low (2.8 mM) or high (16.7 mM) concentrations (n = 8 for each).

(B) Changes in intracellular Ca^{2+} concentration in MIN6-K cells treated with glucose at high (16.7 mM) concentration (n = 6 for each).

(C) Phosphorylation of CREB in MIN6-K cells treated with or without 30 µM 6-Bnz-cAMP-AM (6-Bnz), a PKA-selective cAMP analog, under the basal condition (2.8 mM glucose) (n = 10 for each).

(D) Activation of Rap1 in MIN6-K cells treated with or without 5 µM 8-pCPT-2'-*O*-Me-cAMP-AM (8-pCPT), an Epac-selective cAMP analog, under the basal condition (2.8 mM glucose) (n = 6 for each).

The data are expressed as means ± SEM. Results are representative of 3 independent experiments. Welch's t test was used for evaluation of statistical significance. $*p < 0.05$, $**p < 0.01$. A representative blot for each experiment is shown (C and D). Quantification of autoradiography is shown with corresponding bars positioned under the bands. The intensity of the phosphorylated-CREB and the Rap1-GTP signal was normalized by that of total CREB and Rap1, respectively. Phosphorylated-CREB and Rap1-GTP in the cells treated with analogs are presented as relative to those without analogs, which are considered as 1.

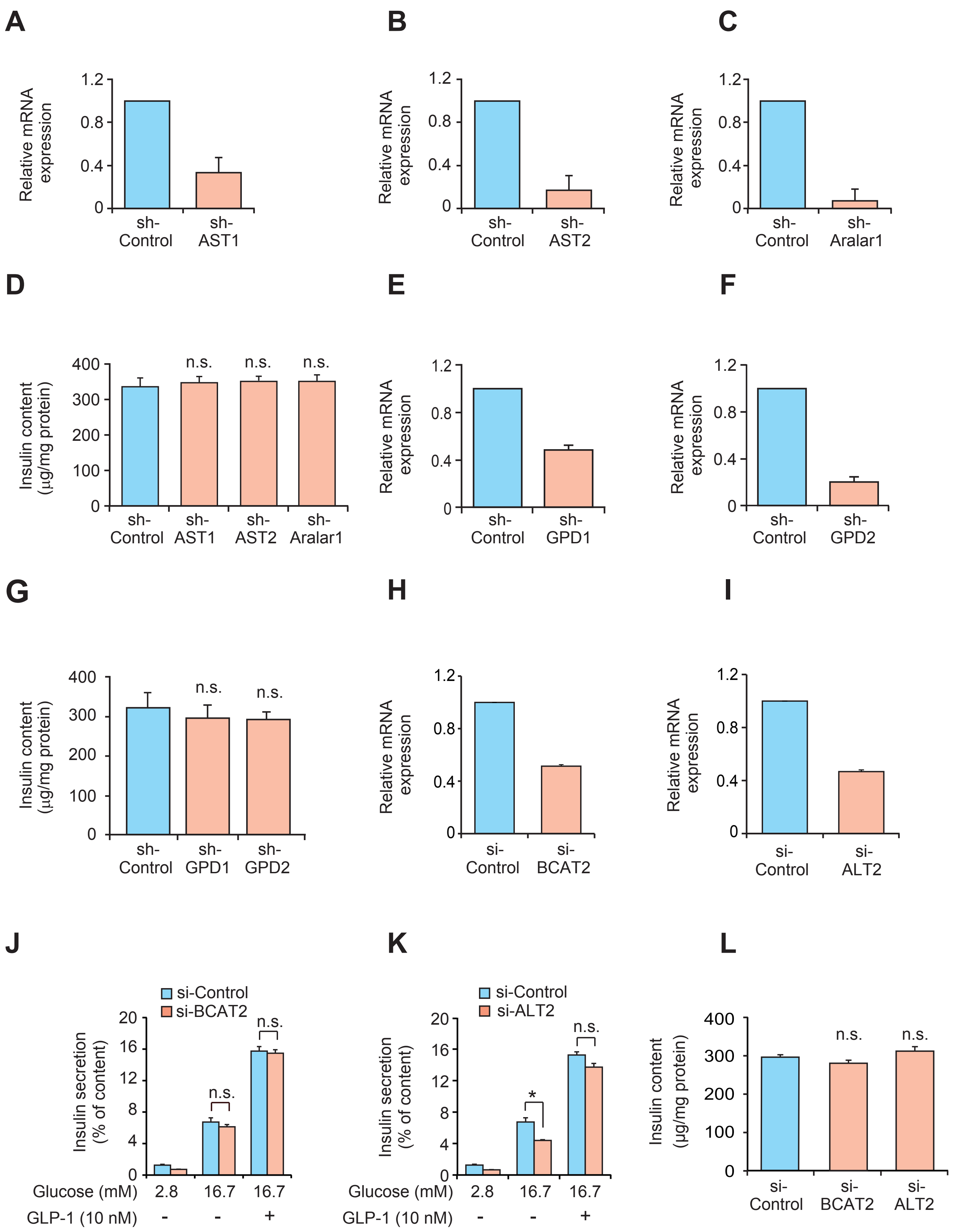

A
Relative mRNA expression
1.2
0.8
0.4
0
sh-Control
sh-AST1
B
Relative mRNA expression
sh-Control
sh-AST2
C
Relative mRNA expression
sh-Control
sh-Aralar1
D
Insulin content (µg/mg protein)
400
300
200
100
0
n.s.
sh-Control
sh-AST1
sh-AST2
sh-Aralar1
E
Relative mRNA expression
sh-Control
sh-GPD1
F
Relative mRNA expression
sh-Control
sh-GPD2
G
Insulin content (µg/mg protein)
n.s.
sh-Control
sh-GPD1
sh-GPD2
H
Relative mRNA expression
si-Control
si-BCAT2
I
Relative mRNA expression
si-Control
si-ALT2
J
si-Control
si-BCAT2
Insulin secretion (% of content)
20
16
12
8
4
0
n.s.
Glucose (mM) 2.8 16.7 16.7
GLP-1 (10 nM) - - +
K
si-Control
si-ALT2
Insulin secretion (% of content)
*
n.s.
Glucose (mM) 2.8 16.7 16.7
GLP-1 (10 nM) - - +
L
Insulin content (µg/mg protein)
n.s.
si-Control
si-BCAT2
si-ALT2

Figure S2. Effect of Knockdown (KD) of AST1, AST2, Aralar1, GPD1, GPD2, BCAT2, or ALT2 on mRNA Expression, Insulin Secretion, and Insulin Content in MIN6-K8 Cells, related to Figure 2

(A-C) Relative mRNA expression levels of AST1 (A), AST2 (B), and Aralar1 (C) (n = 3 for each).

(D) Effect of KD of AST1, AST2, or Aralar1 on insulin content (n = 4-8 for each).

(E and F) Relative mRNA expression levels of GPD1 (E) and GPD2 (F) (n = 3 for each).

(G) Effect of KD of GPD1 or GPD2 on insulin content (n = 4-8 for each).

(H and I) Relative mRNA expression levels of BCAT2 (H) and ALT2 (I) (n = 3 for each).

(J and K) Effect of KD of BCAT2 (J) or ALT2 (K) on insulin secretion (n = 4-8 for each).

(L) Effect of KD of BCAT2 or ALT2 on insulin content (n = 4-8 for each).

The data are expressed as means ± SEM. Results are representative of 3 independent experiments. mRNA expression levels in the respective KD cells are presented as relative to those in sh- or si-Control cells, which are considered as 1. Dunnett's method was used for evaluation of statistical significance versus sh- or si-Control (D, G, and L). Welch's t test was used for evaluation of statistical significance (J and K). $*p < 0.05$; n.s., not significant.

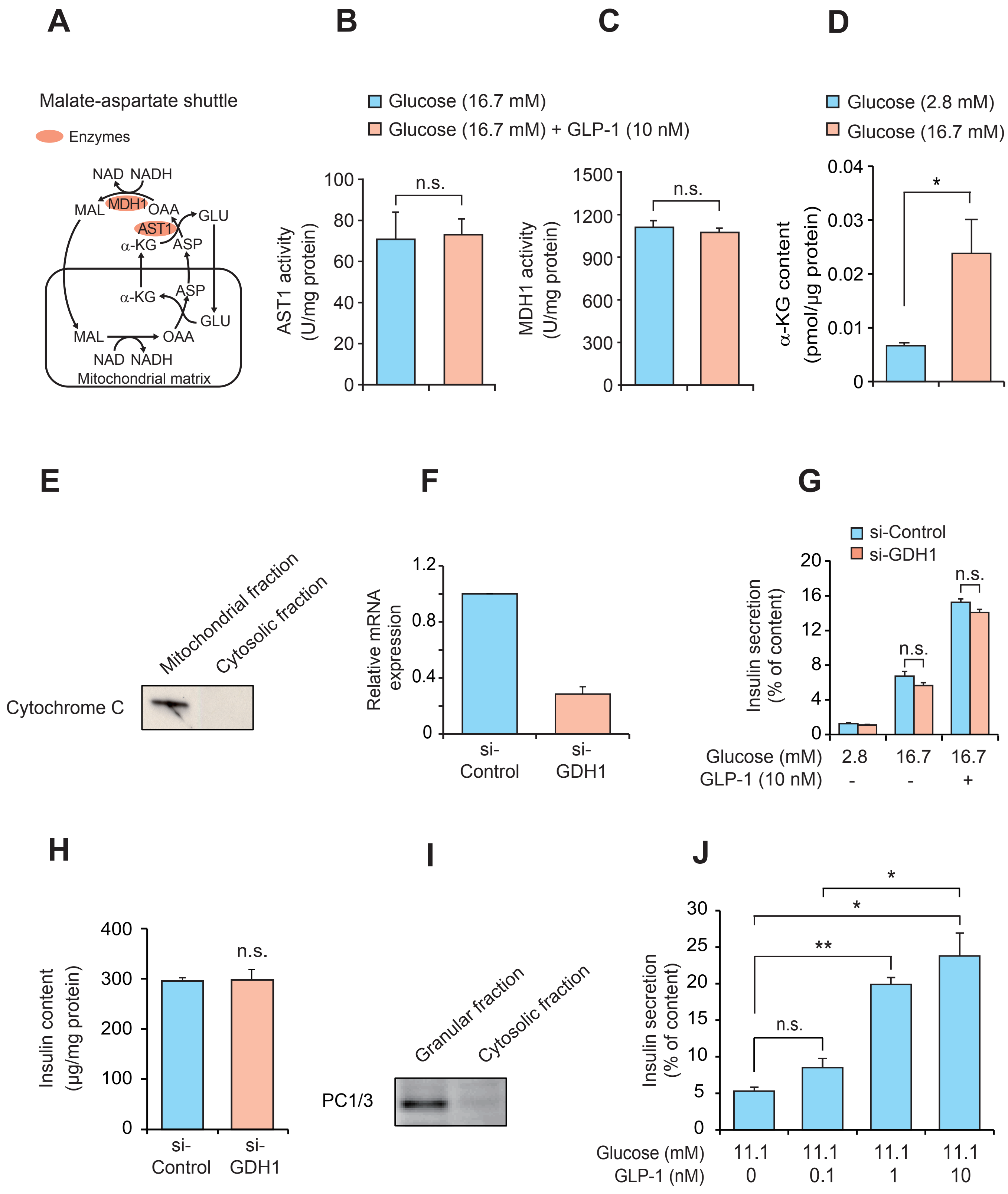

A
Malate-aspartate shuttle
Enzymes
NAD NADH
MAL MDH1 OAA
AST1
GLU
α-KG ASP
α-KG ASP
GLU
MAL OAA
NAD NADH
Mitochondrial matrix
B
Glucose (16.7 mM)
Glucose (16.7 mM) + GLP-1 (10 nM)
AST1 activity (U/mg protein)
n.s.
C
MDH1 activity (U/mg protein)
n.s.
D
Glucose (2.8 mM)
Glucose (16.7 mM)
α-KG content (pmol/μg protein)
*
E
Mitochondrial fraction
Cytosolic fraction
Cytochrome C
F
Relative mRNA expression
si-Control
si-GDH1
G
si-Control
si-GDH1
Insulin secretion (% of content)
n.s.
n.s.
Glucose (mM) 2.8 16.7 16.7
GLP-1 (10 nM) - - +
H
Insulin content (μg/mg protein)
n.s.
si-Control
si-GDH1
I
Granular fraction
Cytosolic fraction
PC1/3
J
Insulin secretion (% of content)
n.s.
**
*
*
Glucose (mM) 11.1 11.1 11.1 11.1
GLP-1 (nM) 0 0.1 1 10

Figure S3. Effect of GLP-1 on Activity of AST1 or MDH1, Effect of glucose on α-ketoglutarate content, Effect of GDH1 KD on Insulin Secretion, and Dose-dependent Effect of GLP-1 on Insulin Secretion in MIN6-K8 Cells, related to Figure 3

(A) Malate-aspartate shuttle. AST1, aspartate aminotransferase 1; MDH1, malate dehydrogenase 1. See also the legend to Figure 1E.

(B and C) Effects of GLP-1 on activities of AST1 (B) and MDH1 (C) ($n = 4$ for each).

(D) Effect of glucose on total cellular α-ketoglutarate contents in MIN6-K8 cells ($n = 3$ for each).

(E) Mitochondrial fraction was confirmed by immunoblot analysis using the antibody against cytochrome C, a marker for mitochondria.

(F) Relative mRNA expression levels of GDH1 ($n = 3$ for each).

(G) Effect of KD of GDH1 on insulin secretion (n = 4-8 for each).

(H) Effect of KD of GDH1 on insulin content (n = 4-8 for each).

(I) Granular fraction was confirmed by immunoblot analysis using the antibody against PC1/3, a marker of insulin granules.

(J) Dose-dependent effects of GLP-1 on insulin secretion under the glucose (11.1 mM)-stimulated condition (n = 4-8 for each).

The data are expressed as means ± SEM. Results are representative of 3 independent experiments. mRNA expression levels in the respective KD cells are presented as relative to those in si-Control cells, which are considered as 1. Welch's t test (B-D, G, and H) or Tukey-Kramer method (J) were used for evaluation of statistical significance. *$p < 0.05$; **$p < 0.01$; n.s., not significant.

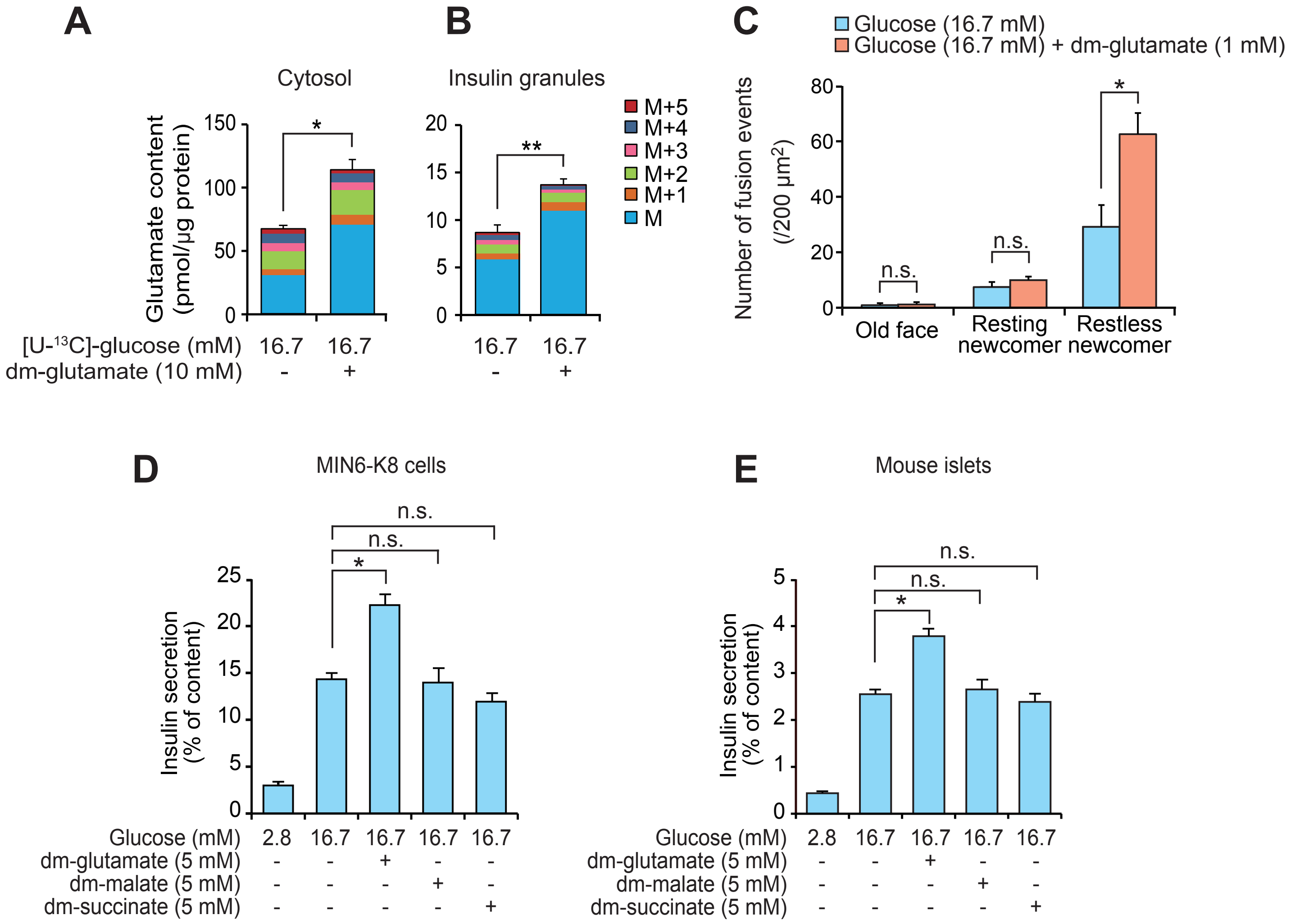

Figure S4. Effects of Dimethyl-Glutamate on Glutamate Contents in the Cytosol and Insulin Granules and on Dynamics of Insulin Granule Exocytosis, and Effect of Dimethyl-Glutamate, -Malate, or -Succinate on Insulin Secretion, related to Figure 4

(A and B) Effect of dimethyl-glutamate on glutamate contents in the cytosol (A) and insulin granules (B) in MIN6-K8 cells (n = 3-6 for each). Changes in contents of glutamate isotopomers (M to M+5) in the cytosol (A) and insulin granules (B) under the glucose-stimulated condition in the absence or presence of dimethyl-glutamate (dm-glutamate) were assessed by analysis of ^{13}C-enriched glutamate (n = 3-6 for each). Treatment of MIN6-K8 cells with dimethyl-glutamate increased the contents of M glutamate (no substitution with ^{13}C) but not M+2, M+3, M+4, or M+5 glutamate isotopomers (derived from [U-^{13}C]-glucose) in insulin granules as well as the cytosol, showing that dimethyl-glutamate is converted to glutamate within the granules as well as the cytosol.

(C) Effect of dimethyl-glutamate on dynamics of insulin granule exocytosis in primary cultured mouse β-cells. Distribution of fusion events of insulin granules (n = 4 for each). Insulin granule exocytosis occurs in three different modes, based on the dynamics of the insulin granules: Old face, granules that are predocked to the plasma membrane and fused to the membrane by stimulation; Resting newcomer, granules that are newly recruited, docked and fused to the plasma membrane by stimulation; and Restless newcomer, granules that are newly recruited and immediately fused to the plasma membrane by stimulation (Shibasaki et al., 2007).

(D and E) Effect of dimethyl-glutamate (5 mM), -malate (5 mM), or -succinate (5 mM) on insulin secretion under the glucose (16.7 mM)-stimulated condition in MIN6-K8 cells (D) and mouse pancreatic islets (E) (n = 4-8 for each).

The data are expressed as means ± SEM. Results are representative of 3 independent experiments. Welch's t test (A-C) or Dunnett's method (D and E) were used for evaluation of statistical significance. *$p < 0.05$; **$p < 0.01$; n.s., not significant.

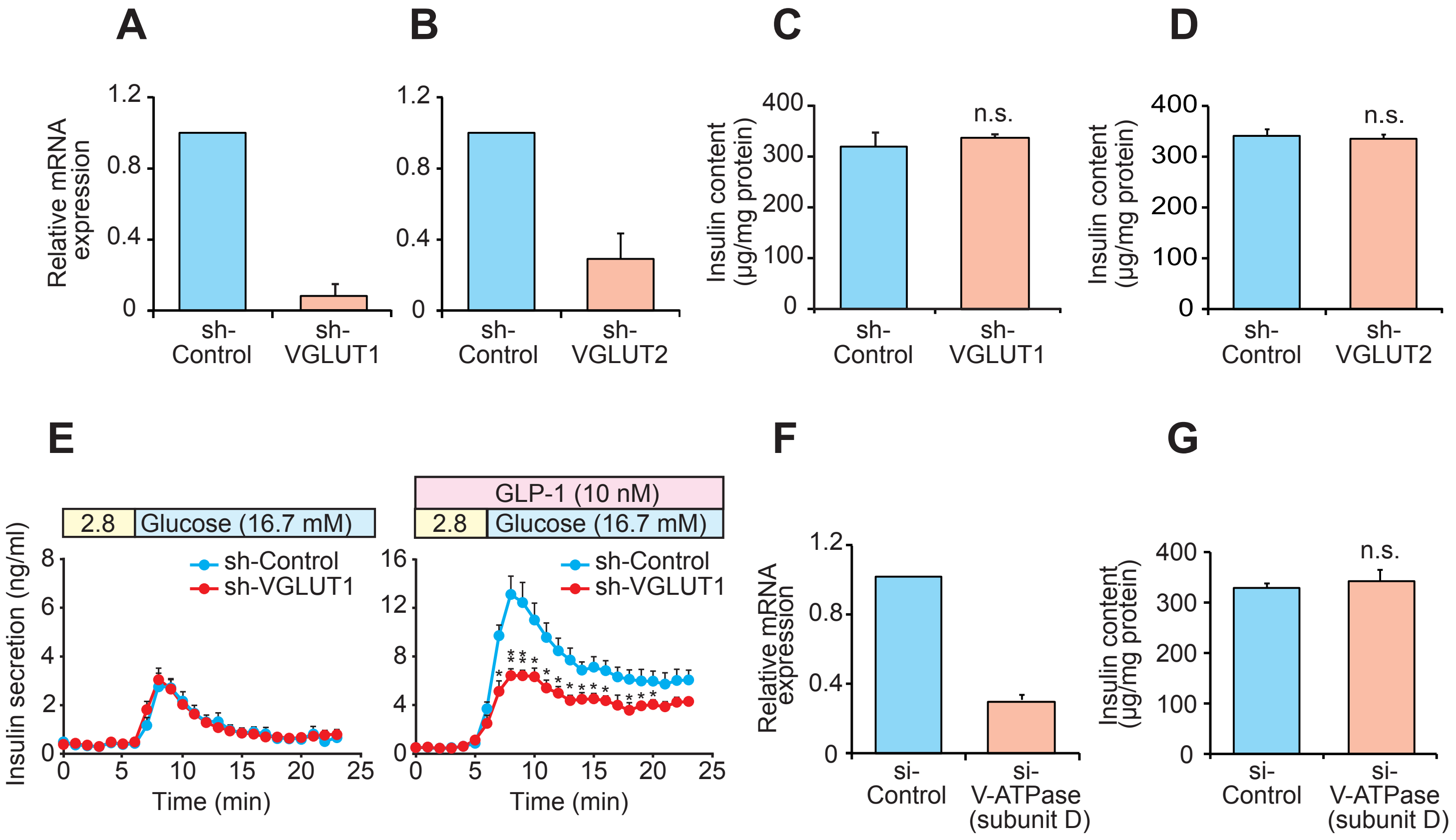

Figure S5. Effect of KD of VGLUT1, VGLUT2, or V-ATPase subunit D on mRNA Expression and Insulin Content and the Effect of KD of VGLUT1 on Dynamics of Insulin Secretion in MIN6-K8 Cells, related to Figure 5

(A and B) Relative mRNA expression levels of VGLUT1 (A) and VGLUT 2 (B) (n = 3 for each). mRNA expression levels in the respective KD cells are presented as relative to those in sh-Control cells, which are considered as 1.

(C and D) Effect of KD of VGLUT1 (C) or VGLUT 2 (D) on insulin contents (n = 3 for each).

(E) Effect of KD of VGLUT1 on dynamics of insulin secretion from perifused MIN6-K8 cells (n = 3-5 for each point). Glucose concentration was switched from 2.8 mM to 16.7 mM at 5 min in the absence (left) or presence (right) of GLP-1. “2.8” indicates 2.8 mM glucose.

(F) Relative mRNA expression level of V-ATPase subunit D (n = 3 for each). mRNA expression level in the KD cells is presented as relative to those in si-Control cells, which are considered as 1.

(G) Effects of KD of V-ATPase subunit D on insulin content (n = 3 for each).

The data are expressed as means ± SEM. Results are representative of 3 independent experiments. Welch’s t test was used for evaluation of statistical significance (C-E and G). *p < 0.05; **p < 0.01; n.s., not significant.

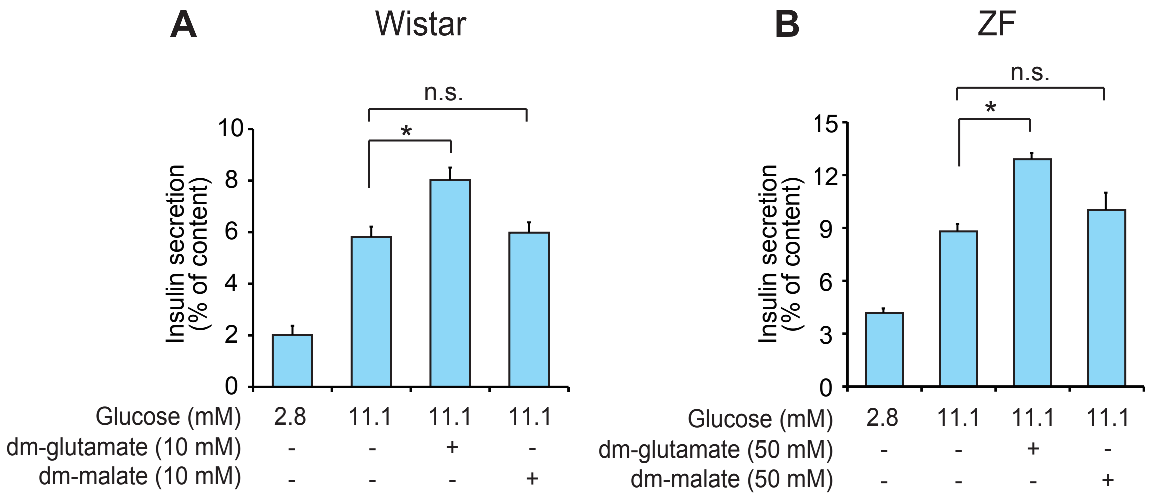

Figure S6. Effect of Dimethyl-Glutamate (10 or 50 mM) or -Malate (10 or 50 mM) on Insulin Secretion from Pancreatic Islets of Wistar (A) and ZF (Zucker Fatty) (B) Rats (n = 4-8 for each), related to Figure 6

The data are expressed as means ± SEM. Results are representative of 3 independent experiments. Dunnett's method was used for evaluation of statistical significance. *$p < 0.05$; n.s., not significant.

Table S1. Comparative Metabolome Analysis of MIN6-K8 and -K20 β-Cell Lines, related to Figure 1

		Glucose (2.8 mM)		Glucose (16.7 mM)			
		Fold change	p-value	Fold change	p-value	C.V. (%)	I.S.
	Glucose 6-phosphate	1.04	0.819	2.34	*0.021	10.1	PIPES
	Fructose 6-phosphate	2.28	0.228	2.20	**0.010	5.4	PIPES
	Fructose 1,6-bisphosphate	0.73	0.180	3.03	**0.0003	15	PIPES
Glycolysis	Dihydroxyacetone phosphate	n.d.		n.d.		41.7	PIPES
	Glyceraldehyde 3-phosphate	n.d.		n.d.		9.5	PIPES
	1,3-bisphosphoglycerate	0.49	0.422	1.90	0.367	8.8	PIPES
	2- and 3-phosphoglycerate	0.83	0.730	0.63	0.282	9.3	PIPES
	Phosphoenolpyruvate	0.69	0.313	0.82	0.616	12.8	PIPES
	Acetyl CoA	n.d.		n.d.		7.3	PIPES
	Citrate	1.04	0.826	1.49	0.135	12.9	PIPES
	Isocitrate	n.d.		n.d.		12.6	PIPES
TCA cycle	α-ketoglutarate	n.d.		n.d.		10.6	PIPES
	Succinate	1.11	0.829	0.76	0.606	13.3	PIPES
	Fumarate	n.d.		n.d.		30.5	PIPES
	Malate	0.83	0.526	0.46	0.175	11.9	PIPES
	6-phosphogluconate	1.00	0.996	1.10	0.696	14.1	PIPES
Pentose	Ribulose 5-phosphate	1.35	0.668	1.04	0.882	27.1	PIPES
phosphate	Ribose 5-phosphate	0.58	0.089	0.92	0.879	11.3	PIPES
pathway	Sedoheptulose 7-phosphate	0.80	0.208	0.70	0.328	11.1	PIPES
	Erythrose 4-phosphate	n.d.		n.d.		11.5	PIPES
Glycogenesis	Glucose 1-phosphate	1.23	0.740	0.92	0.815	7.2	PIPES
	UDP-glucose	2.95	0.258	1.67	0.053	4.4	PIPES
	AMP	0.85	0.130	0.95	0.608	9.1	PIPES
	ADP	0.81	0.335	1.05	0.728	5.4	PIPES
	ATP	1.10	0.684	1.62	*0.045	7.9	PIPES
	cAMP	0.61	0.053	0.56	0.074	11	PIPES
	GMP	1.15	0.498	0.97	0.856	8.5	PIPES
	GDP	0.65	0.199	0.95	0.753	6.5	PIPES
	GTP	0.95	0.887	1.68	*0.020	10.6	PIPES
	NAD	1.09	0.624	1.36	0.082	14.1	PIPES
	NADH	3.54	0.051	4.81	**0.005	14.7	PIPES
	FAD	0.71	0.062	1.02	0.900	15.3	PIPES
	NADP	1.23	0.485	0.83	0.375	21.7	PIPES
Nucleotides	NADPH	0.61	0.060	0.83	0.318	8.9	PIPES
	ADP-ribose	n.d.		n.d.		12.2	PIPES
	cADP-ribose	n.d.		n.d.		11.5	PIPES
	UMP	1.01	0.934	0.85	0.381	7.8	PIPES
	UDP	0.89	0.655	1.83	**0.005	7.2	PIPES
	UTP	0.87	0.675	2.39	0.168	16.3	PIPES
	CMP	0.76	0.112	0.94	0.663	8.7	PIPES
	CDP	0.80	0.105	1.05	0.752	3.6	PIPES
	CTP	0.79	0.270	1.70	0.129	16.8	PIPES
	TMP	n.d.		n.d.		17.7	PIPES
	TDP	n.d.		n.d.		6.5	PIPES
	TTP	n.d.		n.d.		6.2	PIPES
	Glutamate	1.84	*0.027	2.09	**0.005	11.1	$MetSO_2$
	Glutamine	0.93	0.712	1.22	0.377	10.9	$MetSO_2$
	Serine	0.78	0.444	0.97	0.921	21.3	$MetSO_2$
	Glycine	1.11	0.607	1.41	0.167	18.9	$MetSO_2$
	Arginine	1.71	0.130	1.19	0.557	15.5	$MetSO_2$
	Proline	0.84	0.439	1.17	0.347	15.3	$MetSO_2$
	Aspartate	1.68	0.174	3.11	**0.007	16.5	$MetSO_2$
	Asparagine	n.d.		n.d.		20.5	$MetSO_2$
	Alanine	1.24	0.516	1.58	0.098	21.9	$MetSO_2$
Amino acids	Cysteine	n.d.		n.d.		n.d	
	Leucine	1.27	0.734	1.24	0.404	14.3	$MetSO_2$
	Isoleucine	0.72	0.394	1.03	0.895	20.1	$MetSO_2$
	Phenylalanine	1.06	0.904	1.00	0.990	11.5	$MetSO_2$
	Lysine	0.87	0.375	1.17	0.675	12.9	$MetSO_2$
	GABA	0.70	0.214	1.08	0.569	21.2	$MetSO_2$
	Threonine	0.94	0.793	0.89	0.673	23.4	$MetSO_2$
	Tyrosine	0.77	0.437	1.05	0.792	15.4	$MetSO_2$
	Tryptophane	0.89	0.794	1.06	0.773	11.7	$MetSO_2$
	Histidine	0.81	0.311	0.94	0.760	11.9	$MetSO_2$
	Valine	0.72	0.480	1.20	0.333	19.2	$MetSO_2$

Fold change for each metabolite was calculated by dividing content of metabolite of K8 cells by that of K20 cells (n = 3 each).

Welch's t test was used for evaluation of statistical significance. *$p < 0.05$; **$p < 0.01$.

Coefficient of variation (C.V.) for each metabolite was calculated from several repeated measurements of standard mixtures in the same batch.

I.S., internal standards. MetSO2, methionine sulfone. n.d., not detected.

Table S2. Comparative Metabolome Analysis of MIN6-K8 β-Cell Lines Stimulated by Glucose Alone and Glucose plus GLP-1, related to Figure 3

		Glucose (16.7 mM) + GLP-1 (10 nM)		Glucose (16.7 mM) + GLP-1 (100 nM)	
		Fold change	p-value	Fold change	p-value
Glycolysis	Glucose 6-phosphate and Fructose 6-phospahte	1.01	1.000	1.04	0.953
	Fructose 1,6-bisphosphate	1.29	0.626	1.14	0.972
	Dihydroxyacetone phosphate	n.d.		n.d.	
	Glyceraldehyde 3-phosphate	n.d.		n.d.	
	Glycelol 3-phosphate	1.14	0.790	1.00	1.000
	1,3-bisphosphoglycerate	n.d.		n.d.	
	2- and 3-phosphoglycerate	1.18	0.867	1.18	0.800
	Phosphoenolpyruvate	1.00	1.000	1.04	0.998
TCA cycle	Acetyl CoA	1.76	0.528	1.25	0.687
	Citrate	0.99	1.000	1.06	0.985
	Isocitrate	n.d.		n.d.	
	α-ketoglutarate	n.d.		n.d.	
	Succinyl CoA	n.d.		n.d.	
	Succinate	0.86	0.867	0.85	0.394
	Fumarate	n.d.		n.d.	
	Malate	1.05	0.996	1.20	0.786
Pentose phosphate pathway	6-phosphogluconate	1.44	0.650	1.07	0.958
	Ribulose 5-phosphate	n.d.		n.d.	
	Ribose 5-phosphate	n.d.		n.d.	
	Sedoheptulose 7-phosphate	1.12	0.995	0.72	0.768
	Erythrose 4-phosphate	n.d.		n.d.	
Glycogenesis	Glucose 1-phosphate	1.93	0.234	1.67	0.275
	UDP-glucose	1.16	0.965	1.58	0.252
Nucleotides	AMP	0.93	0.936	0.87	0.643
	ADP	1.28	0.781	1.25	0.681
	ATP	1.01	1.000	1.03	1.000
	cAMP	1.79	*0.027	1.91	*0.032
	GMP	0.93	0.992	0.83	0.942
	GDP	1.40	0.509	1.22	0.706
	GTP	1.86	0.518	1.41	0.585
	NAD	1.15	0.842	1.05	0.994
	NADH	1.02	0.100	0.95	0.999
	FAD	1.13	0.936	1.20	0.799
	NADP	1.09	0.929	0.99	1.000
	NADPH	n.d.		n.d.	
	ADP-ribose	1.50	0.669	0.70	0.529
	cADP-ribose	0.90	0.998	1.40	0.784
	UMP	1.15	0.920	0.85	0.846
	UDP	1.13	0.949	1.00	1.000
	UTP	1.49	0.645	1.25	0.559
	CMP	0.84	0.620	0.50	0.129
	CDP	1.23	0.768	0.93	0.972
	CTP	1.55	0.534	1.23	0.771
	TMP	n.d.		n.d.	
	TDP	n.d.		n.d.	
	TTP	1.66	0.242	0.88	0.980
Amino acids	Glutamate	0.98	0.999	0.97	0.996
	Glutamine	1.10	0.955	0.79	0.512
	Serine	1.16	0.677	2.91	0.247
	Glycine	1.01	1.000	1.19	0.737
	Arginine	0.84	0.893	0.98	1.000
	Proline	1.17	0.848	1.35	0.570
	Aspartate	1.05	0.926	1.07	0.943
	Asparagine	1.00	1.000	0.85	0.751
	Alanine	1.04	0.998	1.04	0.997
	Cysteine	n.d.		n.d.	
	Leucine	1.29	0.373	2.01	0.345
	Isoleucine	1.04	0.996	1.59	0.384
	Phenylalanine	1.07	0.979	1.61	0.471
	Lysine	0.92	0.987	1.31	0.518
	GABA	1.08	0.989	1.18	0.909
	Threonine	1.25	0.413	1.22	0.697
	Tyrosine	1.17	0.900	2.40	0.280
	Tryptophane	0.88	0.768	1.71	0.520
	Histidine	1.20	0.675	3.72	0.299
	Valine	1.11	0.924	1.84	0.382

Fold change for each metabolite was calculated by dividing content of metabolite of glucose plus GLP-1-stimulated K8 cells by that of glucose-stimulated cells (n = 3 each).

Dunnett's method was used for evaluation of statistical significance. $*p < 0.05$. n.d., not detected.

Supplemental Experimental Procedures

Reagents

Aminooxyacetate (AOA), dimethyl-glutamate, dimethyl-malate, dimethyl-succinate, and Evans blue were purchased from Tokyo Chemical Industry (Tokyo, Japan), Bafilomycin A1 was purchased from Wako (Osaka, Japan), [U-^{13}C]-glucose and H-89 were purchased from Sigma-Aldrich (St. Louis, MO), 8-pCPT-2'-*O*-Me-cAMP-AM (8-pCPT) and 6-Bnz-cAMP-AM (6-Bnz) were purchased from Biolog-Life Science Institute (Bremen, Germany), anti-PC1/3 antibody was purchased from Merck Millipore (Billerica, MA), Anti-cytochrome C antibody was purchased from Molecular Probes (Eugene, OR), and anti-VGLUT1 antibody (Hayashi et al., 2003) was provided by Y. Moriyama.

ATP Content

MIN6-K cells were preincubated for 30 min in HEPES-balanced Krebs-Ringer bicarbonate buffer containing 0.1% BSA (H-KRB) with 2.8 mM glucose and then incubated for 30 min in H-KRB with 2.8 mM or 16.7 mM glucose. After the incubation, these cells were lysed with cell culture lysis reagent (Promega, Wisconsin). The amount of ATP was measured with an ATP bioluminescence assay kit (Roche Diagnostics, Basel, Switzerland).

Intracellular Ca^{2+} Concentration

Cells were loaded with 5 μM fura-2 AM (Dojindo, Kumamoto, Japan) for 20 min in H-KRB with 0.1 mM glucose. Intracellular Ca^{2+} concentration was measured by a dual-excitation wavelength method (340/380 nm) with a fluorometer (Fluoroskan Ascent CF;

Labsystems, Helsinki, Finland). Data were expressed as the ratio of the fluorescence emission at 340/380 nm after background subtraction.

Metabolome Analysis

MIN6-K8 and -K20 cells (2.3 × 10^6 cells) were preincubated for 60 min in H-KRB with 2.8 mM glucose and then incubated for 30 min in H-KRB with 2.8 mM glucose or 16.7 mM glucose. After the incubation, internal standard solution [40 μl; ribitol (1 mM)/methionine sulfone (1 mM)/PIPES (100 mM)] and extraction solution [800 μl; acetonitrile/H_2O/CH_3Cl (7:3:10, v/v/v)] were added to the cells. The mixture was then homogenized and centrifuged at 20,000 × g under 4°C for 2 min. The supernatant containing hydrophilic primary metabolites was collected and dried in a centrifugal concentrator at room temperature. The dried hydrophilic metabolites were reconstituted with MilliQ water and then subjected to capillary electrophoresis-mass spectrometry (CE-MS) using a P/ACE MDQ (Beckman Coulter, Fullerton, CA) and 4000QTRAP hybrid triple quadrupole linear ion-trap mass spectrometer with Turbo V ion source and CE-MS kit (Applied Biosystems, Foster City, CA). CE-MS analysis was performed as previously described (Harada et al., 2008). Metabolite concentrations (pmol/2.3 × 10^6 cells) were calculated by using the ratio between each metabolite in the sample and standard compounds, and then results were presented as fold change over control.

Insulin Secretion

MIN6-K cells or pancreatic islets were preincubated for 30 min in H-KRB with 2.8 mM glucose and then incubated for 30 min in H-KRB with 2.8 mM glucose, 16.7 mM glucose, or 16.7 mM glucose plus reagents, such as GLP-1, GIP, dimethyl-glutamate, dimethyl-

malate, and dimethyl-succinate. In some experiments, AOA, bafilomycin, or Evans blue were added to the preincubation and incubation mediums. 0.3% FuGENE6 transfection reagent (Roche Applied Science, Mannheim, Germany) was also included in the mediums to increase efficiency of uptake of Evans blue by cells. Insulin released in the incubation buffer and cellular insulin content in MIN6-K cells or pancreatic islets were measured by insulin assay kits from CIS Bio international (Gif sur Yvette, France). The amounts of insulin secretion were normalized by the cellular insulin content determined by acid-ethanol extraction.

Knockdown Experiments

The following sequences that produced significant gene silencing were targeted:

AST1, 5’-GCCTATCAGGGCTTTGCATCT-3’;

AST2, 5’-GCGTTACCGAAGCCTTCAAGA-3’;

Aralar1, 5’-GCAGGAGTAGCTGATCAAACC-3’;

GPD1, 5’-GCTAAATGGGCAGAAGCTACA-3’;

GPD2, 5’-GCTCACAGGGCAGGAATTTGA-3’;

VGLUT1, 5’-GCCATGGCATCTGGAGCAAAT-3’;

VGLUT2, 5’-GCAAATCTGCTAGGTGCAATG-3’.

Non-target shRNA expressing adenoviral vector (negative control) was purchased from Invitrogen Corp (Carlsbad, CA). BLOCK-iT U6 RNAi entry vector and pAd/BLOCK-iT adenoviral vector plasmid (Invitrogen) were used for generation of adenoviruses, according to the manufacturer’s instruction. MIN6-K8 cells were infected with adenovirus carrying shRNA.

For knockdown experiments of BCAT2, ALT2, GDH1, and V-ATPase subunit D,

siGENOME siRNA against these genes and siGENOME Non-Targeting siRNAs were purchased from Dharmacon (Lafayette, CO). MIN6-K8 cells were transfected with siRNAs using DharmaFECT2 transfection reagent (Dharmacon) according to the manufacturer's instructions.

Glutamate Content

For whole cell measurement, MIN6-K8 cells were stimulated for 30 min with various stimuli indicated in the figures. Glutamate contents in lysed cells were determined by Yamasa L-Glutamate Assay Kit II (Yamasa Corporation, Chiba, Japan). For measurement of mitochondrial and cytosolic glutamate, MIN6-K cells were collected after stimulations indicated in the figures and suspended in an isotonic buffer (0.32 M sucrose and 10 mM HEPES-KOH (pH 7.4)). The suspended cells were homogenized and centrifuged at 1000 × g at 4°C for 5 min. The post-nuclear supernatant was centrifuged at 27,000 × g at 4°C for 35 min. The pellet and supernatant were used for the measurement of mitochondrial and cytosolic glutamate contents, respectively. For measurement of glutamate content in insulin granules, insulin granules were obtained from MIN6-K cells as described previously (Bai et al., 2003). Insulin granule fraction was confirmed by immunoblot analysis using anti-PC1/3 antibody. Contents of glutamate were determined by Yamasa L-Glutamate Assay Kit II. Contents of glutamate isotopomers were also measured by ^{13}C-enrichment analysis with uniformly-labeled [U-^{13}C]-glucose as a substrate using CE-MS (Agilent 7100 CE and 6224 TOF LC-MS). Glutamate contents were normalized by protein contents determined by BCA protein assay kit (Thermo Scientific, Waltham, MA) in MIN6-K cells or by DNA contents determined by Quant-iT PicoGreen dsDNA Assay Kit (Invitrogen) in pancreatic islets.

Electrophysiology

Capacitance recordings of single β-cell exocytosis were made in primary mouse β-cells using the standard whole-cell configuration in which the pipette-filling solution dialyzes the cell interior, allowing cAMP and glutamate to be applied intracellularly. The extracellular medium consisted of 118 mM NaCl, 20 mM TEA-Cl, 5.6 mM KCl, 1.2 mM $MgCl_2$, 2.6 mM $CaCl_2$, 5 mM D-glucose, and 5 mM HEPES (pH 7.4 with NaOH). The standard electrode (intracellular) solution contained 90 mM Cs-gluconate, 35 mM CsCl, 1 mM $MgCl_2$, 5 mM HEPES, 0.05 mM EGTA, 3 mM Mg-ATP (pH 7.15 with CsOH). Cs-glutamate was included at concentrations of 1-10 mM gluconate and Cs-gluconate was correspondingly reduced, to maintain iso-osmolarity. Na-cAMP and the PKI (both from Sigma) were included in the intracellular (pipette-filling) medium at the indicated concentrations. The depolarizations were 500 ms long, applied at a frequency of 1 Hz and went from -70 mV to 0 mV.

Immunofluorescence Staining

MIN6-K8 cells were fixed and pretreated as previously described (Yasuda et al., 2010). C57BL/6 mice were perfused transcardially with fixative solution containing 2% formaldehyde in 0.1 M phosphate buffer (pH 7.4). The pancreases were isolated and immersed in the same fixative solution for 5 hours at 4°C. Frozen sections were prepared by a cryostat (Leica, Bensheim, Germany). Cells and tissues were incubated with guinea pig anti-insulin antibody and rabbit anti-VGLUT1 antibody, followed by Alexa Fluor 488-conjugated goat anti-guinea pig IgG antibody and Alexa Fluor 546-conjugated goat anti-rabbit IgG antibody, respectively.

Phosphorylation of CREB and Activation of Rap1

Measurements of phosphorylation of CREB and activation of Rap1 were performed as previously described (Shibasaki et al., 2007; Zhang et al., 2009).

Enzyme Activity

MIN6-K8 cells were incubated for 10 min in the absence or presence of 10 nM GLP-1 with 16.7 mM glucose. The cells were collected and suspended in the isotonic buffer containing protease inhibitors (Complete, EDTA Free; Roche Applied Science). The cells were homogenized and centrifuged at 1,000 × g at 4°C for 5 min. The post-nuclear supernatant (PNS) was centrifuged at 27,000 × g at 4°C for 35 min. The supernatant was used for the measurement of activities of AST1 and MDH1. AST1 activity was determined by Aspartate Transaminase Enzymatic Assay Kit (Bio Scientific, Austin, TX). Activity of MDH1 was measured as previously described (Glatthaar et al., 1974).

Perifusion Experiments

Perifusion experiments on insulin secretion of MIN6-K8 cells were performed as described previously (Sugawara et al., 2009).

Supplemental References

Glatthaar, B.E., Barbarash, G.R., Noyes, B.E., Banaszak, L.J., and Bradshaw, R.A. (1974). The preparation of the cytoplasmic and mitochondrial forms of malate dehydrogenase and aspartate aminotransferase from pig heart by a single procedure. Anal. Biochem. *57*, 432-451.

Harada, K., Ohyama, Y., Tabushi, T., Kobayashi, A., and Fukusaki, E. (2008). Quantitative analysis of anionic metabolites for Catharanthus roseus by capillary electrophoresis using sulfonated capillary coupled with electrospray ionization-tandem mass spectrometry. J. Biosci. Bioeng. *105*, 249-260.

Hayashi, M., Morimoto, R., Yamamoto, A., and Moriyama, Y. (2003). Expression and localization of vesicular glutamate transporters in pancreatic islets, upper gastrointestinal tract, and testis. J. Histochem. Cytochem. *51*, 1375-1390.

Zhang, C.L., Katoh, M., Shibasaki, T., Minami, K., Sunaga, Y., Takahashi, H., Yokoi, N., Iwasaki, M., Miki, T., and Seino, S. (2009). The cAMP sensor Epac2 is a direct target of antidiabetic sulfonylurea drugs. Science *325*, 607-610.
