# Supplementary material for: Glutamate Acts as a Key Signal Linking Glucose Metabolism to Incretin/cAMP Action to Amplify Insulin Secretion
Source: Cell Rep. 2014 Oct 16;9(2):661–73. doi: 10.1016/j.celrep.2014.09.030 (PMC4536302; doi:10.1016/j.celrep.2014.09.030)
Supplement: Document S2. Article plus Supplemental Information [file mmc2.pdf]

Cell Reports

Article

Glutamate Acts as a Key Signal Linking Glucose Metabolism to Incretin/cAMP Action to Amplify Insulin Secretion

Graphical Abstract

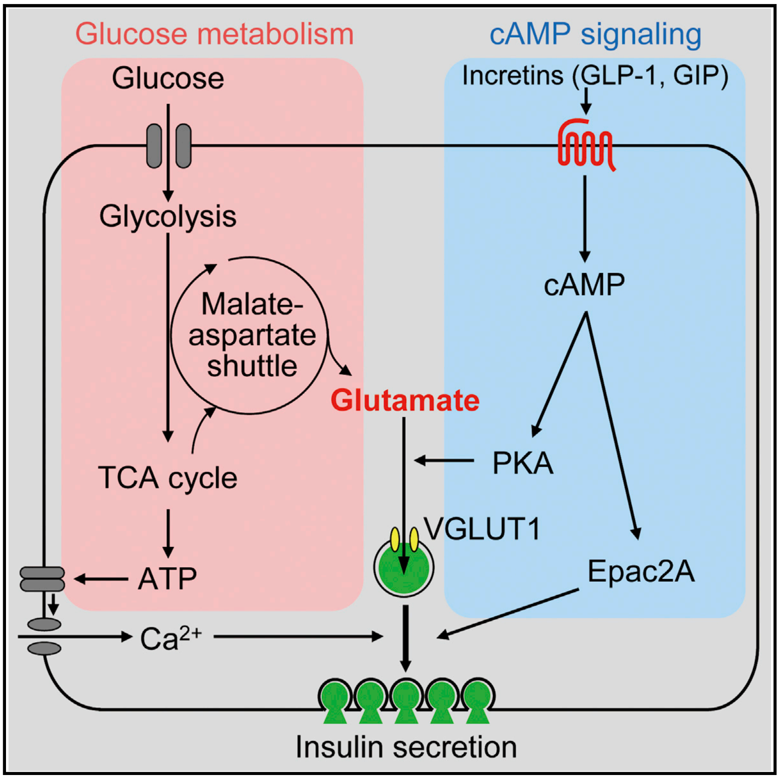

Highlights

Glutamate is derived from the malate-aspartate shuttle upon glucose stimulation

Shuttle-derived glutamate is crucial for incretin-induced insulin secretion

Cytosolic glutamate is transported into insulin granules via cAMP/PKA signaling

Glutamate production by glucose is defective in incretin-unresponsive β cells

Authors

Ghupurjan Gheni, Masahito Ogura, ..., Patrik Rorsman, Susumu Seino

Correspondence

seino@med.kobe-u.ac.jp

In Brief

Gheni et al. find that cytosolic glutamate derived from glucose through the malate-aspartate shuttle is the signal underlying incretin-induced insulin secretion. Glutamate uptake into insulin granules mediated by cAMP/PKA signaling amplifies insulin release. Thus, cytosolic glutamate acts as a signal linking glucose metabolism to incretin/cAMP action to amplify insulin secretion.

Gheni et al., 2014, Cell Reports 9, 661–673

October 23, 2014 ©2014 The Authors

http://dx.doi.org/10.1016/j.celrep.2014.09.030

Cell Reports
Article

OPEN ACCESS
CellPress

Glutamate Acts as a Key Signal Linking Glucose Metabolism to Incretin/cAMP Action to Amplify Insulin Secretion

Ghupurjan Gheni,[1,2,12] Masahito Ogura,[1,5,12] Masahiro Iwasaki,[1,12,13] Norihide Yokoi,[1] Kohtaro Minami,[1,3] Yasumune Nakayama,[6] Kazuo Harada,[7] Benoit Hastoy,[8] Xichen Wu,[9] Harumi Takahashi,[1] Kazushi Kimura,[10] Toshiya Matsubara,[1,11] Ritsuko Hoshikawa,[1] Naoya Hatano,[4] Kenji Sugawara,[1,2] Tadao Shibasaki,[1,3] Nobuya Inagaki,[5] Takeshi Bamba,[6] Akira Mizoguchi,[10] Eiichiro Fukusaki,[6] Patrik Rorsman,[8] and Susumu Seino[1,4,*]
[1]Division of Molecular and Metabolic Medicine
[2]Division of Diabetes and Endocrinology
[3]Division of Cellular and Molecular Medicine
[4]The Integrated Center for Mass Spectrometry
Kobe University Graduate School of Medicine, Chuo-ku, Kobe 650-0017, Japan
[5]Department of Diabetes, Endocrinology and Nutrition, Graduate School of Medicine, Kyoto University, Sakyo-ku, Kyoto 606–8507, Japan
[6]Department of Biotechnology, Graduate School of Engineering
[7]Applied Environmental Biology, Graduate School of Pharmaceutical Sciences
Osaka University, Yamadaoka, Suita 565-0871, Japan
[8]Oxford Centre for Diabetes, Endocrinology and Metabolism, University of Oxford, Churchill Hospital, Oxford OX3 7LJ, UK
[9]Alberta Diabetes Institute, University of Alberta, Faculty of Medicine & Dentistry, Edmonton, AB T6G 2E1, Canada
[10]Department of Neural Regeneration and Cell Communication, Mie University Graduate School of Medicine, Edobashi, Tsu 514-8507, Japan
[11]Life Science Research Center, Technology Research Laboratory, Shimadzu Corporation, Soraku-gun, Kyoto 619-0237, Japan
[12]Co-first author
[13]Present address: Division of Metabolism and Clinical Nutrition, Kansai Electric Power Hospital, Fukushima-ku, Osaka 553-0003, Japan
*Correspondence: seino@med.kobe-u.ac.jp
http://dx.doi.org/10.1016/j.celrep.2014.09.030
This is an open access article under the CC BY license (http://creativecommons.org/licenses/by/3.0/).

SUMMARY

Incretins, hormones released by the gut after meal ingestion, are essential for maintaining systemic glucose homeostasis by stimulating insulin secretion. The effect of incretins on insulin secretion occurs only at elevated glucose concentrations and is mediated by cAMP signaling, but the mechanism linking glucose metabolism and cAMP action in insulin secretion is unknown. We show here, using a metabolomics-based approach, that cytosolic glutamate derived from the malate-aspartate shuttle upon glucose stimulation underlies the stimulatory effect of incretins and that glutamate uptake into insulin granules mediated by cAMP/PKA signaling amplifies insulin release. Glutamate production is diminished in an incretin-unresponsive, insulin-secreting β cell line and pancreatic islets of animal models of human diabetes and obesity. Conversely, a membrane-permeable glutamate precursor restores amplification of insulin secretion in these models. Thus, cytosolic glutamate represents the elusive link between glucose metabolism and cAMP action in incretin-induced insulin secretion.

INTRODUCTION

Insulin secretion from pancreatic β cells is precisely regulated by various intracellular signals to maintain blood glucose levels within a normal range. Impaired insulin secretion contributes to the pathogenesis and pathophysiology of diabetes (Polonsky et al., 1988; Porte, 1991) and is a target for its treatment. According to the consensus model of glucose-induced insulin secretion (GIIS), GIIS depends on a series of carefully orchestrated β cell responses: mitochondrially generated ATP results in closure of ATP-sensitive K^+ (K_{ATP}) channels, which in turn triggers membrane depolarization, electrical activity, and opening of voltage-dependent Ca^{2+} channels (VDCCs), with the resultant elevation of $[Ca^{2+}]_i$ initiating Ca^{2+}-induced insulin granule exocytosis (Henquin, 2000). Thus, ATP produced by glucose metabolism is a critical signal in GIIS.

Pancreatic β cells are equipped with two highly active NADH shuttles linked to glycolysis: the malate-aspartate shuttle and the glycerol phosphate shuttle, both of which contribute to ATP production. Whereas inhibition of either one of the NADH shuttles does not affect GIIS, inhibition of both shuttles abolishes GIIS (Eto et al., 1999). In addition, other intracellular signals in pancreatic β cells, including cAMP and phospholipid-derived molecules such as inositol 1,4,5-triphosphate (IP3) and diacylglycerol (DAG), which are evoked by various nutrients and hormonal and neuronal inputs, exert important modulatory functions of insulin secretion in the maintenance of systemic glucose homeostasis.

CrossMark

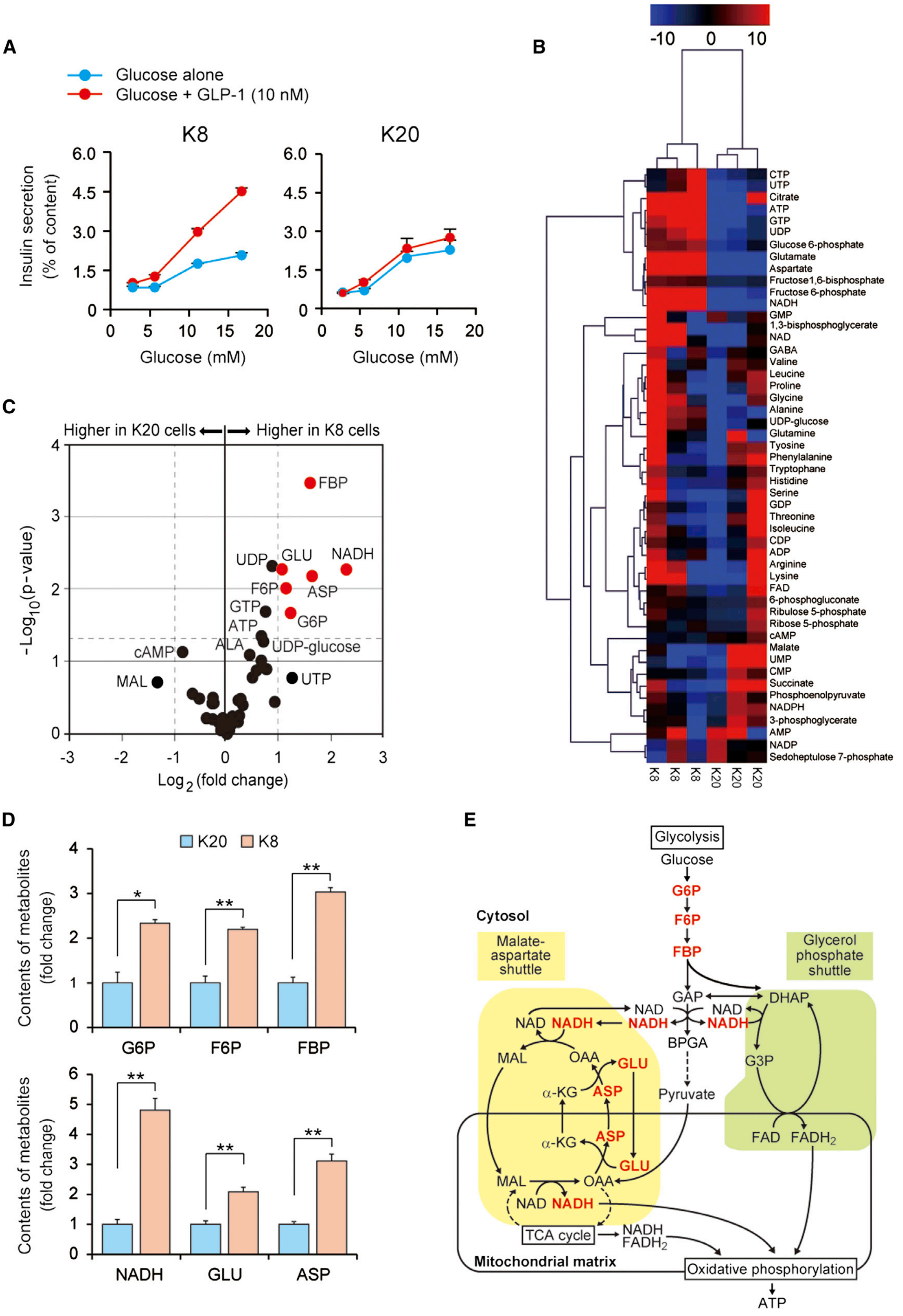

(legend on next page)

Incretins such as glucagon-like peptide 1 (GLP-1) and glucose-dependent insulinotropic polypeptide (GIP) are secreted by the enteroendocrine L cells and K cells, respectively, in response to meal ingestion (Cataland et al., 1974; Kreymann et al., 1987) and are critical for preventing postprandial hyperglycemia by amplifying insulin secretion through cAMP signaling (Drucker, 2006; Holst, 2007). It is well known that incretin/cAMP signaling stimulates insulin secretion in a glucose-dependent manner (Siegel and Creutzfeldt, 1985; Prentki and Matschinsky, 1987; Weir et al., 1989). Importantly, type 2 diabetes is associated with impaired incretin-induced insulin secretion (Nauck et al., 1993; Seino et al., 2010). The identification of the amplifying effect of incretins in insulin secretion has paved the way for recently developed incretin-based diabetes therapies that carry less risk for hypoglycemia (Ahrén, 2009; Drucker and Nauck, 2006).

Recent studies have shown that incretin/cAMP signaling in insulin secretion involves both protein kinase A (PKA)- and Epac2A-dependent pathways (Seino and Shibasaki, 2005). PKA phosphorylates various proteins associated with the insulin secretory process, such as Snapin (Song et al., 2011), MyRIP, Rabphilin (Brozzi et al., 2012), and Rip11 (Sugawara et al., 2009). On the other hand, Epac2A, which contains a guanine nucleotide exchange factor domain, activates the small G-proteins Rap1 and Rap2 upon cAMP binding (Bos, 2006). Epac2A/Rap1 signaling plays a key role in incretin-induced insulin secretion, likely by promoting recruitment of insulin granules and/or fusion events of the granules to the plasma membrane (Shibasaki et al., 2007; Seino et al., 2011) or granule fusion itself (Eliasson et al., 2003).

Glucose metabolism in pancreatic β cells is essential for both triggering insulin secretion by glucose and amplifying insulin secretion by incretin/cAMP signaling, but the mechanism of the link between glucose metabolism and incretin/cAMP action in insulin secretion has not been elucidated. Here, we employed a differential metabolomics-based approach to address this issue using incretin-responsive and -unresponsive β cell lines. We find that cytosolic glutamate derived from the malate-aspartate shuttle upon glucose stimulation is transported into insulin granules by cAMP/PKA signaling, which leads to amplification of insulin granule exocytosis. Our data highlight the role of cytosolic glutamate as a key signal linking glucose metabolism to incretin/cAMP action to amplify insulin secretion.

RESULTS

Profiles of Glucose Metabolism Differ between Incretin-Responsive and -Unresponsive Mouse Pancreatic β Cell Lines

We utilized two recently established β cell lines, designated MIN6-K8 and MIN6-K20 cells (Iwasaki et al., 2010), as incretin-responsive and -unresponsive β cell models, respectively, to investigate the mechanism of incretin-induced insulin secretion. Like primary pancreatic β cells, MIN6-K8 cells secrete insulin in response to both glucose and the incretins GLP-1 and GIP, whereas MIN6-K20 cells respond to glucose, but not to the incretins (Figures 1A, S1A, and S1B). We ascertained the integrity of downstream cAMP signaling targets of cAMP (PKA and Epac2A, as assessed by phosphorylation of cAMP response element-binding protein [CREB] or Rap1 activity, respectively) in both MIN6-K8 and MIN6-K20 cells (Figures S1C and S1D). Likewise, no differences in the capacity for cAMP production in response to GLP-1 or GIP in these cells were detected (Iwasaki et al., 2010). These findings indicate that the difference in incretin responsiveness between MIN6-K8 and MIN6-K20 cells is not due to disruption of the incretin/cAMP signaling pathways. Since incretin-induced insulin secretion is glucose dependent, we considered the possibility that the impaired incretin responsiveness of MIN6-K20 cells results from compromised "metabolism-cAMP coupling." We addressed this possibility by conducting a metabolome analysis of MIN6-K8 and MIN6-K20 cells stimulated by glucose (16.7 mM) (Table S1). Hierarchical cluster multivariate analysis revealed that MIN6-K8 and MIN6-K20 cells were separated into two distinct clusters, indicating differences in the metabolic response to glucose stimulation (Figure 1B). Univariate analysis (fold change and t test) showed that the contents of glucose 6-phosphate (G6P), fructose 6-phosphate (F6P), fructose 1,6-bisphosphate (FBP), NADH, glutamate (GLU), and aspartate (ASP) were significantly higher in MIN6-K8 cells than in MIN6-K20 cells (Figures 1C and 1D; Table S1). These results suggest higher activity of the malate-aspartate shuttle in incretin-responsive MIN6-K8 cells than in incretin-unresponsive MIN6-K20 cells (Figure 1E).

Essential Role of the Malate-Aspartate Shuttle in Incretin-Induced Insulin Secretion

We then examined the role of the malate-aspartate shuttle in GIIS and incretin-induced insulin secretion (as assessed by

Figure 1. Distinct Profiles of Glucose Metabolism in Incretin-Responsive (MIN6-K8) and -Unresponsive (MIN6-K20) Cells

(A) Insulin secretory responses to glucose alone and glucose plus GLP-1 in MIN6-K8 (left) and MIN6-K20 (right) cells (n = 5–8 for each point).

(B) Metabolomic profiles expressed as a heatmap in MIN6-K8 and MIN6-K20 cells under the glucose (16.7 mM)-stimulated condition (n = 3 for each).

(C) Univariate analysis of metabolome data on MIN6-K8 and MIN6-K20 cells under the glucose (16.7 mM)-stimulated condition (n = 3 for each). Welch's t test p values and fold changes are shown as a volcano plot. Each dot indicates a metabolite. Metabolites showing a >2-fold [$|\mathrm{Log}_2$ (fold change)$| > 1$)] and statistically significant [$p < 0.05$: -Log_{10} (p value) > 1.3] difference between the two cell lines are indicated in red. ALA, alanine; ASP, aspartate; F6P, fructose 6-phophate; FBP, fructose 1,6-bisphosphate; G6P, glucose 6-phosphate; GLU, glutamate; MAL, malate.

(D) Contents of metabolites showing the difference between MIN6-K8 and MIN6-K20 cells under the glucose (16.7 mM)-stimulated condition (n = 3 for each). See also the legend to (C).

(E) Schematic view of how the two NADH shuttles (malate-aspartate shuttle and glycerol phosphate shuttle) are linked to glycolysis. Metabolites showing a difference between MIN6-K8 and MIN6-K20 cells are indicated in red. α-KG, α-ketoglutarate; ASP, aspartate; BPGA, 1,3-bisphosphoglycerate; DHAP, dihydroxyacetone phosphate; F6P, fructose 6-phophate; FBP, fructose 1,6-bisphosphate; G3P, glycerol 3-phosphate; G6P, glucose 6-phosphate; GAP, glyceraldehyde 3-phosphate; GLU, glutamate; MAL, malate; OAA, oxaloacetate. See also the legend to (C).

The data are expressed as means ± SEM. Results are representative of three independent experiments. Welch's t test was used for evaluation of statistical significance (C and D). *$p < 0.05$; **$p < 0.01$. See also Table S1 and Figure S1.

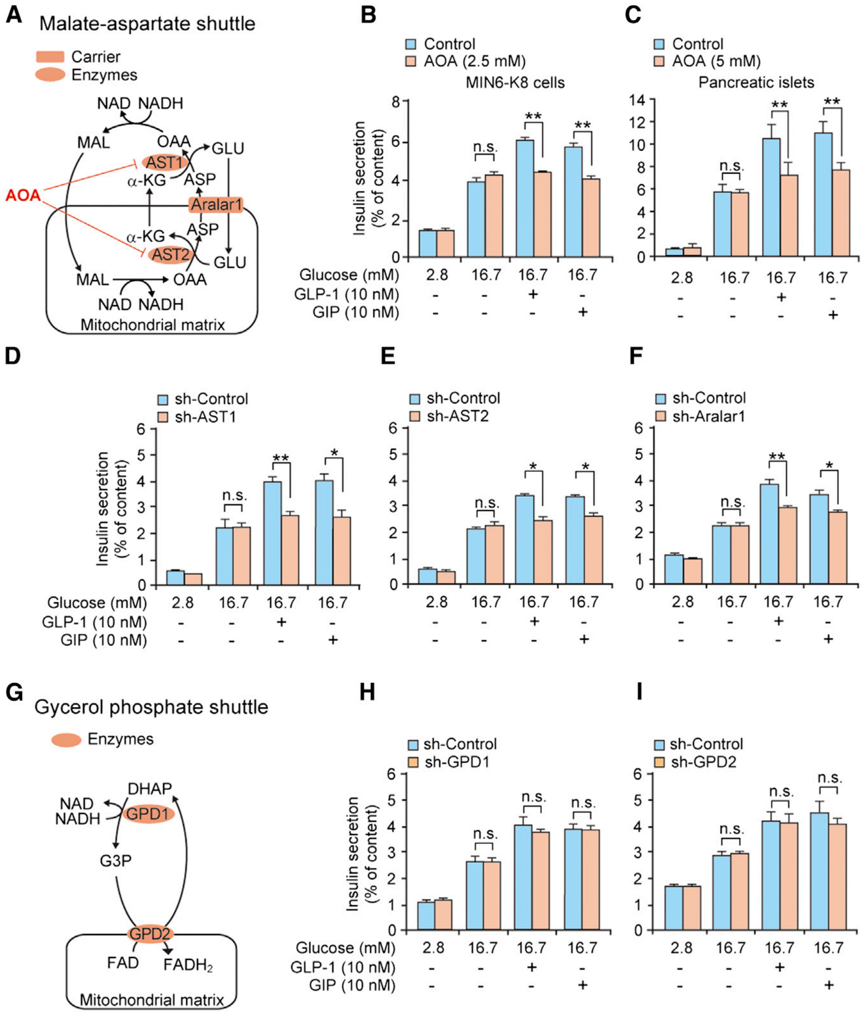

Figure 2. Essential Role of the Malate-Aspartate Shuttle in Incretin-Induced Insulin Secretion

(A) Malate-aspartate shuttle. Aralar1, aspartate/glutamate carrier; AST1 and AST2, aspartate aminotransferase 1 and 2, respectively. See also the legend to Figure 1E.

(B and C) Effect of AOA, an inhibitor of the malate-aspartate shuttle, on insulin secretion from MIN6-K8 cells (B) and mouse pancreatic islets (C). The concentrations of AOA used were 2.5 mM in MIN6-K8 cells and 5 mM in mouse pancreatic islets.

(D–F) Effects of KD of AST1 (D), AST2 (E), and Aralar1 (F) on insulin secretion from MIN6-K8 cells.

(G) Glycerol phosphate shuttle. GPD1 and GPD2, glycerol 3-phosphate dehydrogenase 1 and 2, respectively. See also the legend to Figure 1E.

(H and I) Effects of KD of GPD1 (H) and GPD2 (I) on insulin secretion from MIN6-K8 cells.

The data are expressed as means ± SEM (n = 4–8). Results are representative of three independent experiments. Welch's t test was used for evaluation of statistical significance. $^*p < 0.05$; $^{**}p < 0.01$; n.s., not significant. See also Figure S2.

amplification of insulin secretion by GLP-1 and GIP) by using aminooxyacetate (AOA), an inhibitor of the shuttle (Eto et al., 1999; MacDonald, 1982; Figure 2A). We found that AOA did not affect GIIS but virtually abolished the response to GLP-1 or GIP in MIN6-K8 cells (Figure 2B). Very similar observations were made in primary mouse pancreatic islets treated with AOA (Figure 2C). These results suggest that whereas activity of the malate-aspartate shuttle is essential for incretin-induced insulin secretion, this is not the case for GIIS. To further confirm the role of the malate-aspartate shuttle, we next examined the effects of knockdown (KD) of the aspartate aminotransferases AST1 and AST2 or the aspartate/glutamate carrier Aralar1 on insulin secretion in MIN6-K8 cells. As illustrated schematically in Figure 2A, these enzymes are all required for malate-aspartate shuttle activity. Reduced expression of AST1 (−68%; Figure S2A), AST2 (−83%; Figure S2B), or Aralar1 (−93%; Figure S2C) did not affect GIIS, but decreased incretin-induced insulin secretion (Figures 2D–2F). By contrast, KD of glycerol 3-phosphate dehydrogenases GPD1 (−52%; Figure S2E) or GPD2 (−80%; Figure S2F), both of which are required for activity of the glycerol phosphate shuttle (another NADH shuttle that is linked to glycolysis; Figure 2G), affected neither GIIS nor incretin-induced secretion (Figures 2H and 2I). The possibility that AOA has effects on other transaminases was ruled out by KD experiments with branched-chain aminotransferase 2 (BCAT2) and alanine aminotransferase 2 (ALT2) (Figures S2H–S2K). We ascertained that KD of these enzymes/carrier did not affect cellular insulin content (Figures S2D, S2G, and S2L). Thus, while GIIS is maintained following abolition of either the malate-aspartate or glycerol phosphate shuttle, incretin-induced insulin secretion depends exclusively on the malate-aspartate shuttle.

Glucose-Dependent Production of Cytosolic Glutamate and cAMP/PKA-Dependent Glutamate Transport into Insulin Granules

We next attempted to clarify the relationship between the malate-aspartate shuttle and incretin stimulation. We first considered direct incretin-induced activation of the shuttle. However, metabolome analysis revealed (with the exception of cAMP; Table S2) no differences in the contents of the metabolites associated with glycolysis and the malate-aspartate shuttle in MIN6-K8 cells stimulated by glucose (16.7 mM) alone or glucose plus GLP-1 (10 nM or 100 nM). In addition, the activities of AST1 and malate dehydrogenase MDH1, both of which are required for malate-aspartate shuttle activity (Figure S3A), were not increased by GLP-1 (Figures S3B and S3C). These results indicate that incretin-induced insulin secretion is not caused by direct activation of the malate-aspartate shuttle. We therefore explored the alternative possibility that a metabolite associated with the malate-aspartate shuttle mediates the effect on insulin

secretion. We focused on glutamate since it was increased in response to glucose in incretin-responsive MIN6-K8 cells and was previously proposed to be a signal in insulin secretion (Maechler and Wollheim, 1999). Cytosolic glutamate is converted from α-ketoglutarate through the malate-aspartate shuttle upon glucose stimulation (Figure 2A). We therefore hypothesized that cytosolic glutamate might mediate incretin-induced insulin secretion. Total cellular glutamate content was increased in a glucose-concentration-dependent manner, but was not affected by GLP-1 (Figure 3A). α-Ketoglutarate content also was increased by glucose stimulation (Figure S3D). We next used mass spectrometry to determine ^{13}C enrichment of glutamate in MIN6-K8 cells exposed to [U-^{13}C]-glucose. The cytosolic contents of M and M+1 glutamate isotopomers (no substitution with ^{13}C derived from [U-^{13}C]-glucose), both of which are naturally existing in cells, were unchanged by glucose stimulation, whereas M+2, M+3, M+4, and M+5 glutamate isotopomers (two to five ^{13}C substitutions for ^{12}C) were increased significantly (Figures 3B and 3C). GLP-1 did not alter the distribution of glutamate isotopomers produced by glucose.

We then investigated the involvement of the malate-aspartate shuttle in glutamate production using preparations of whole cells and cytosolic and mitochondrial fractions of MIN6-K8 cells with or without AOA treatment. We found that cytosolic glutamate contributed to the majority of cellular glutamate under glucose stimulation and that treatment with AOA markedly suppressed the production of both cellular and cytosolic glutamate (Figures 3D–3F and S3E). In addition, neither GIIS nor incretin-induced insulin secretion was affected by KD of glutamate dehydrogenase 1 (GDH1; −72%; Figures S3F–S3H), the enzyme that catalyzes the production of mitochondrial glutamate from α-ketoglutarate. Together, these results suggest that glucose increases cytosolic glutamate through the malate-aspartate shuttle.

Importantly, glutamate content in insulin granules was not increased by glucose (16.7 mM) alone, but was increased significantly by the addition of GLP-1 (Figures 3G and S3I). The increase in granular glutamate content by GLP-1 was blocked by H-89, a PKA inhibitor, whereas the glutamate content was not increased by 8-pCPT-2′-*O*-Me-cAMP-AM, an Epac-selective cAMP analog (Figure 3G), indicating that glutamate content in insulin granules is increased by a cAMP/PKA-dependent mechanism. By analyzing ^{13}C-enriched glutamate using [U-^{13}C]-glucose as a substrate, we confirmed that GLP-1 increased the amounts of M+2 to M+5 glutamate isotopomers (two to five ^{13}C substitutions for ^{12}C) in insulin granules in a concentration-dependent manner (Figure 3H), a finding that is consistent with the effect of GLP-1 on insulin secretion (Figure S3J). These results suggest that cytosolic glutamate derived from α-ketoglutarate through the malate-aspartate shuttle represents a signal that mediates incretin-induced insulin secretion.

Glutamate as a Signal in Incretin-Induced Insulin Secretion

To clarify whether glutamate acts as a signal in incretin-induced insulin secretion, we next investigated the role of glutamate in cAMP-induced insulin granule exocytosis. We examined the effect of increasing concentrations of cytosolic glutamate on depolarization-evoked exocytosis in pancreatic β cells using the standard whole-cell technique in conjunction with measurements of membrane capacitance (ΔC_m) (Rorsman and Renström, 2003) in the absence and presence of cAMP. Whereas glutamate (3 and 10 mM) stimulated exocytosis in the absence of cAMP, consistent with previous reports (Høy et al., 2002), the effect was minute compared with the much stronger amplification seen in the presence of cAMP (Figure 4A). The effect of glutamate on exocytosis in the presence of 100 μM cAMP was not mimicked by malate (10 mM; data not shown). Exocytosis evoked by glutamate in the presence of cAMP was inhibited by application of PKI (10 μM), a PKA-inhibitory peptide (Figure 4B). Together with the finding that the increase in glutamate content in insulin granules by GLP-1 was blocked by H-89 (Figure 3G), these results indicate that glutamate acts as a signal in cAMP-induced exocytosis in a PKA-dependent manner.

To further confirm that glutamate acts as an amplifying signal in insulin secretion, we examined the effect of dimethyl-glutamate, a membrane-permeable glutamate precursor (Maechler and Wollheim, 1999). We found that dimethyl-glutamate is converted to glutamate in insulin granules as well as in the cytosol (Figures S4A and S4B), as assessed by analysis of ^{13}C-enriched glutamate in MIN6-K8 cells. Dimethyl-glutamate amplified both the first and second phases of glucose-induced insulin granule exocytosis (analyzed by total internal reflection fluorescence microscopy [TIRFM]) (Shibasaki et al., 2007; Figures 4C and S4C). In incretin-unresponsive MIN6-K20 cells, cytosolic glutamate content was not increased at all by glucose (Figure 4D), but dimethyl-glutamate markedly amplified insulin secretion (Figure 4E). As dimethyl-malate and dimethyl-succinate have no effects on insulin secretion (Figures S4D and S4E), it is unlikely that dimethyl-glutamate is used as a fuel to stimulate insulin secretion. These results indicate that dimethyl-glutamate mimics the effect of incretin/cAMP on insulin secretion. Collectively, these findings corroborate the view that glutamate acts as an amplifying signal in incretin-induced insulin secretion.

Requirement of Glutamate Transport into Insulin Granules for Amplification of Insulin Secretion by Incretin/cAMP Signaling

Glutamate is transported into secretory vesicles in neurons (Naito and Ueda, 1983) and enteroendocrine cells (Uehara et al., 2006) through vesicular glutamate transporters (VGLUTs) (Bellocchio et al., 2000; Gras et al., 2002; Omote et al., 2011; Takamori et al., 2001). VGLUT1, VGLUT2, and VGLUT3 are expressed in the pancreatic β cell lines βTC6, RINm5F, and INS-1E, and their insulin granules have the capacity to accumulate glutamate (Bai et al., 2003; Gammelsaeter et al., 2011). Analysis by quantitative real-time RT-PCR showed that VGLUT1 is the predominant VGLUT in MIN6-K8 cells (Figure 5A). Immunocytochemical and immunoblot analyses revealed that VGLUT1 colocalizes with insulin granules (Figures 5B and 5C). We then examined the role of glutamate transport into insulin granules in insulin secretion using Evans blue, an inhibitor of glutamate transport into secretory vesicles (Maechler and Wollheim, 1999; Roseth et al., 1995), and KD of VGLUT1 (−94%; Figure S5A). The two procedures yielded identical responses: GIIS was not affected, but the incretin-induced stimulation was abolished (Figures 5D and 5E). By

OPEN ACCESS
CellPress

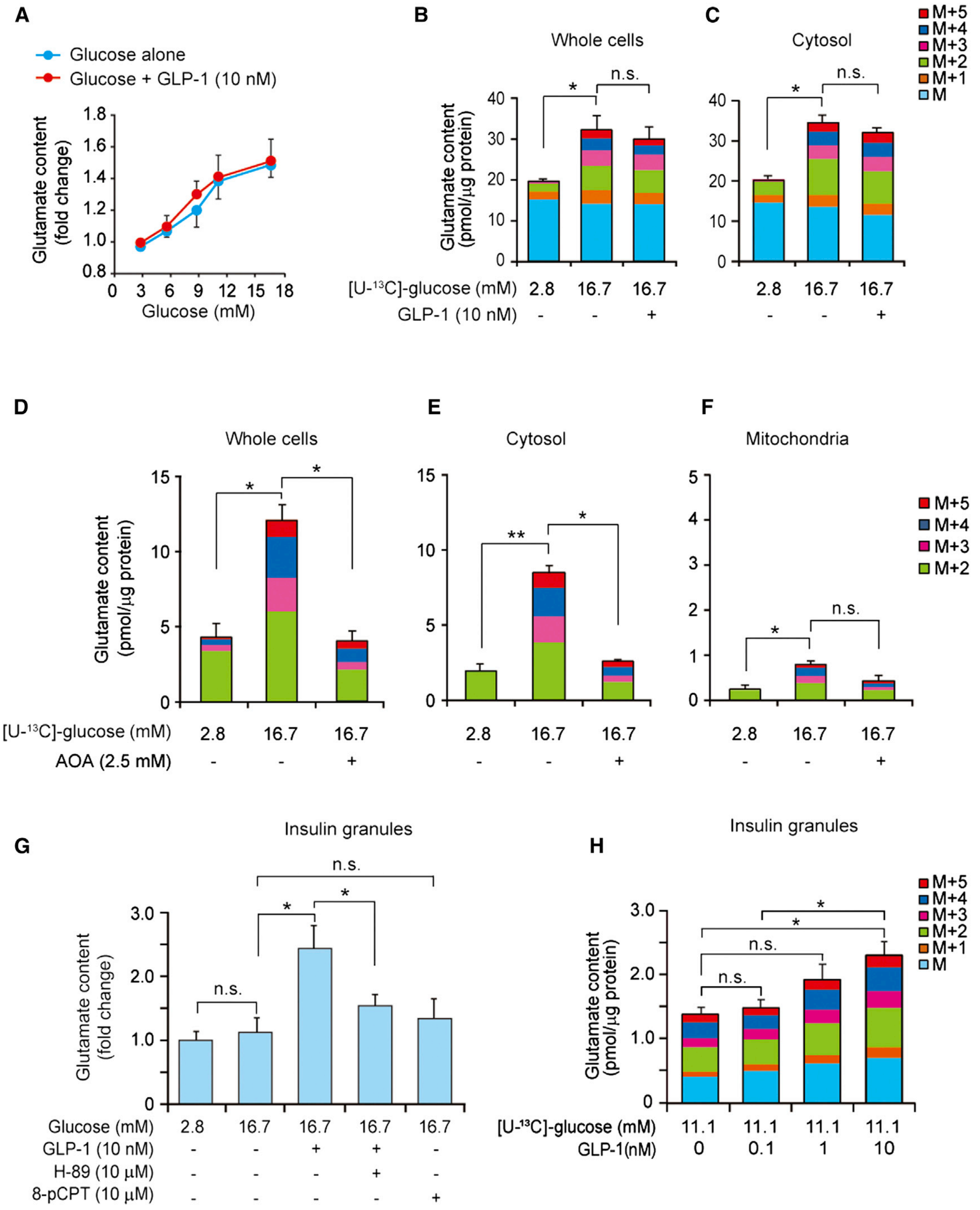

Figure 3. Glucose-Dependent Production of Cytosolic Glutamate and Increased Glutamate Contents in Insulin Granules by cAMP/PKA Signaling

(A) Effect of glucose on total cellular glutamate contents in the absence or presence of GLP-1 (10 nM) in MIN6-K8 cells (n = 3 for each point).

(B and C) Changes in contents of glutamate isotopomers (M to M+5) by glucose stimulation in the absence or presence of GLP-1 (10 nM) in whole cells (B) and cytosol (C) in MIN6-K8 cells (n = 4–5 for each).

(D–F) Effects of AOA on contents of glutamate isotopomers (M+2 to M+5) in whole cells (D), cytosol (E), and mitochondria (F) in MIN6-K8 cells (n = 3 for each).

(G) Effects of glucose, GLP-1 (10 nM), H-89 (10 μM, a PKA inhibitor), and 8-pCPT (10 μM, 8-pCPT-2′-*O*-Me-cAMP-AM, an Epac-selective cAMP analog) on glutamate contents in insulin granules in MIN6-K8 cells (n = 4–12).

(H) Dose-dependent effects of GLP-1 on glutamate contents in insulin granules in MIN6-K8 cells under the glucose (11.1 mM)-stimulated condition (n = 4 for each). The data are expressed as means ± SEM. Results are representative of three independent experiments. The Tukey-Kramer method was used for evaluation of statistical significance. *p < 0.05; **p < 0.01; n.s., not significant. See also Table S2 and Figure S3.

OPEN
ACCESS
CellPress

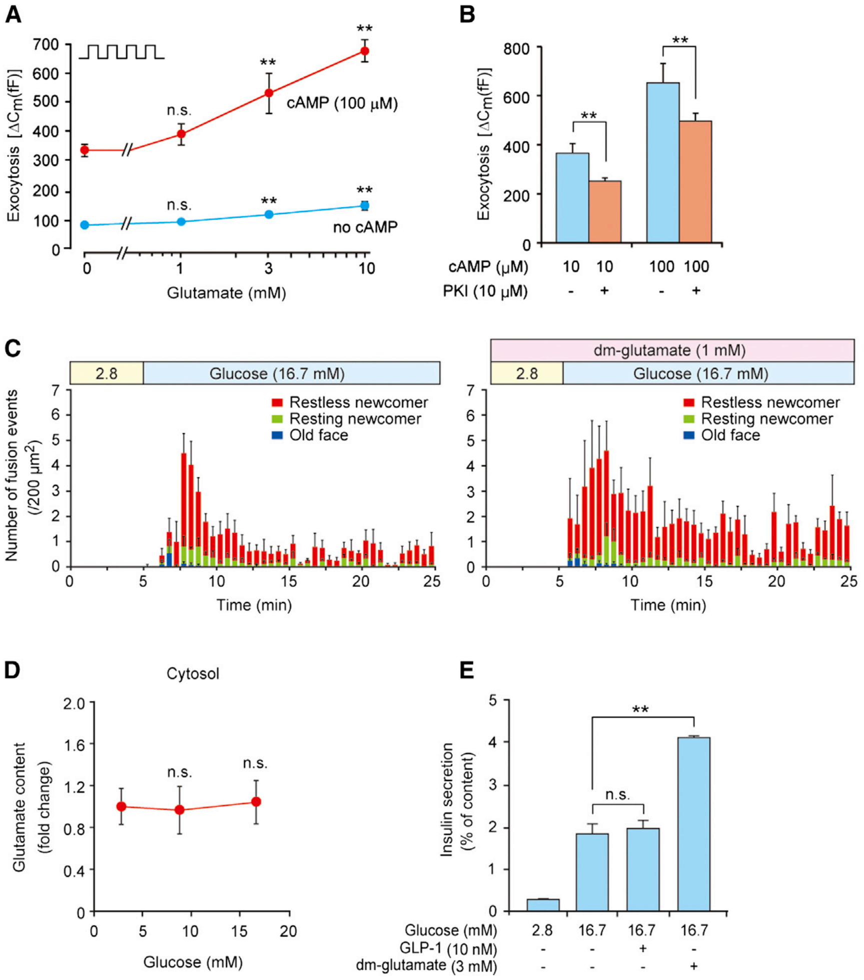

Figure 4. Glutamate as a Signal in Incretin-Induced Insulin Granule Exocytosis

(A) Effect of intracellular glutamate on exocytosis. Exocytosis (ΔC_m) was measured in a single mouse β cell at concentrations of 0, 1, 3, and 10 mM intracellular glutamate (added via the recording electrode) in the presence or absence of 100 μM cAMP (n = 19–31 for each point). Exocytosis was elicited by trains of four 500 ms depolarizations from −70 mV to zero mV applied at 1 Hz (indicated schematically in the upper-left corner). All data points in the presence of cAMP are significantly different from corresponding values in the absence of cAMP ($p < 0.05$ or better). Glutamate (3 and 10 mM) stimulates exocytosis in both the absence and presence of cAMP as compared with the relative control (no glutamate), but responses in the absence of cAMP are dwarfed compared with the much larger effects in the presence of cAMP. **$p < 0.01$ versus no glutamate in the absence or presence of cAMP.

(B) Effect of PKI (10 μM), a PKA-inhibitory peptide, on exocytosis. Exocytosis (ΔC_m) was measured in a single mouse β cell in the presence of 3 mM glutamate in cells exposed to 10 and 100 μM cAMP as indicated (n = 20–27).

(C) Effect of dimethyl-glutamate (dm-glutamate), a membrane-permeable glutamate precursor, on insulin granule exocytosis. The exocytosis was measured as fusion events by TIRFM. Histograms show the number of fusion events caused by glucose alone (left) and glucose plus dimethyl-glutamate (right) in primary cultured mouse pancreatic β cells (n = 4 for each); "2.8" indicates 2.8 mM glucose.

(D) Effect of glucose on cytosolic glutamate production in incretin-unresponsive MIN6-K20 cells (n = 7–8 for each point).

(E) Effect of dimethyl-glutamate (dm-glutamate) on insulin secretion from incretin-unresponsive MIN6-K20 cells (n = 4–8 for each).

The data are expressed as means ± SEM. Results are representative of three independent experiments. Student's t test (A and B) and Dunnett's method (D and E) were used for evaluation of statistical significance. **$p < 0.01$; n.s., not significant. See also Figure S4.

contrast, KD of VGLUT2 (−71%; Figure S5B) affected neither GIIS nor incretin-induced insulin secretion (Figure 5F), and KD of either VGLUT did not affect cellular insulin content (Figures S5C and S5D). Dynamic measurements of insulin secretion demonstrated that the amplification by GLP-1 of both the first and second phases of insulin secretion in MIN6-K8 cells was strongly inhibited by KD of VGLUT1 (Figure S5E). We also examined insulin secretion from the pancreatic islets of VGLUT1 knockout (*Slc17a7*$^{-/-}$) mice. GIIS did not differ between wild-type (*Slc17a7*$^{+/+}$) and *Slc17a7*$^{-/-}$ islets, but the stimulatory effect of GLP-1 was not seen in *Slc17a7*$^{-/-}$ islets (Figure 5G). Importantly, dimethyl-glutamate restored amplification of insulin secretion in *Slc17a7*$^{-/-}$ mice (Figure 5G).

We next examined the potential role of V-ATPase, which has been shown to participate in vesicular glutamate transport (Omote et al., 2011), in insulin secretion in MIN6-K8 cells. Neither KD of V-ATPase subunit D (−69%; Figure S5F) nor bafilomycin, an inhibitor of V-ATPase, affected GIIS, but both reduced the amplification evoked by GLP-1 (Figures 5H and 5I) without affecting cellular insulin content (Figure S5G). Intriguingly, dimethyl-glutamate restored insulin secretion in the bafilomycin-treated MIN6-K8 cells (Figure 5I). These results indicate that glutamate transport into insulin granules through VGLUT1 is required for incretin-induced insulin secretion.

Pathophysiological Role of Glutamate in Insulin Secretion

To clarify the relationship between glutamate production and insulin secretion in disease states, we compared GIIS and incretin-induced insulin secretion in nondiabetic Wistar, diabetic Goto-Kakizaki (GK) (Goto et al., 1975), and obese Zucker fatty (ZF) rats (Zucker and Zucker, 1961). The GK rat is a model of non-obese type 2 diabetes with defective insulin secretion associated with impaired glucose metabolism in pancreatic β cells (Ostenson et al., 1993). Although GIIS from the pancreatic islets of GK rats was markedly decreased compared with that of Wistar rats (Figures 6A, 6B, and S6A), the incretins retained their amplifying capacity. Dimethyl-glutamate also amplified insulin secretion in GK rats. In contrast, in ZF rats, a model of obesity with a mutation in the leptin receptor gene, basal insulin

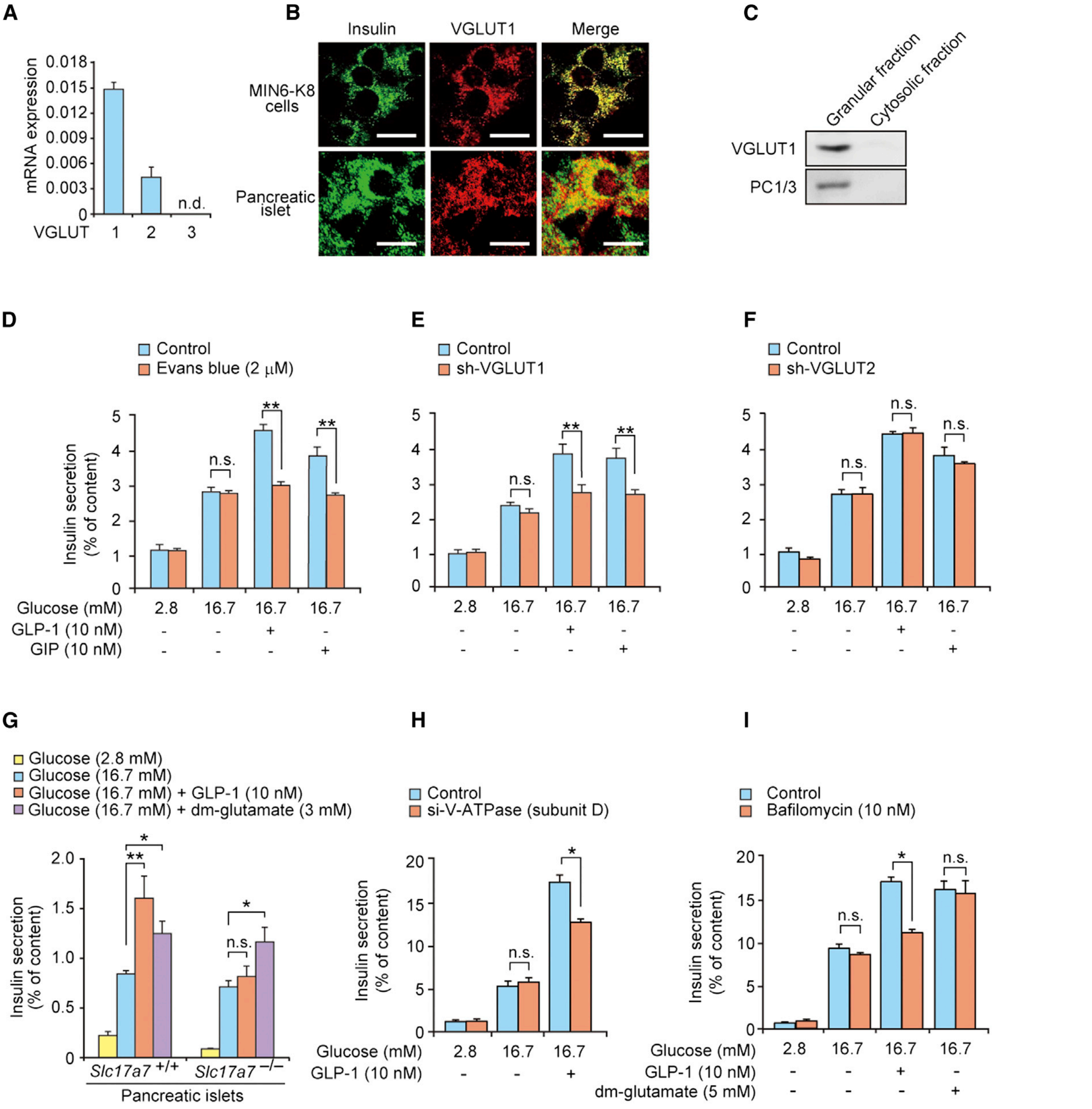

Figure 5. Requirement of Glutamate Transport into Insulin Granules for Amplification of Insulin Secretion by Incretin/cAMP Signaling

(A) mRNA expression levels of VGLUTs in MIN6-K8 cells (n = 3–4 for each). n.d., not detected.

(B) Immunocytochemical analysis of VGLUT1 in MIN6-K8 cells and pancreatic islets. Scale bars, 10 μm.

(C) Immunoblot analysis of VGLUT1 in insulin granules in MIN6-K8 cells. The insulin granule fraction was confirmed by immunoblot analysis using anti-PC1/3 antibody.

(D) Effect of Evans blue, an inhibitor of vesicular glutamate transport, on insulin secretion from MIN6-K8 cells (n = 5–8 for each).

(E and F) Effects of KD of VGLUT1 (E) and VGLUT2 (F) on insulin secretion from MIN6-K8 cells (n = 4–8 for each).

(G) Insulin secretion from pancreatic islets of wild-type (*Slc17a7*$^{+/+}$) and VGLUT1 knockout (*Slc17a7*$^{-/-}$) mice (n = 4–8 for each). dm-glutamate, dimethyl-glutamate.

(H) Effect of KD of V-ATPase subunit D on insulin secretion from MIN6-K8 cells (n = 4 for each).

(I) Effect of bafilomycin, an inhibitor of V-ATPase, on insulin secretion from MIN6-K8 cells (n = 4 for each).

The data are expressed as means ± SEM. Results are representative of three independent experiments. Welch's t test (D–F, H, and I) and Dunnett's method (G) were used for evaluation of statistical significance. *p < 0.05; **p < 0.01; n.s., not significant. See also Figure S5.

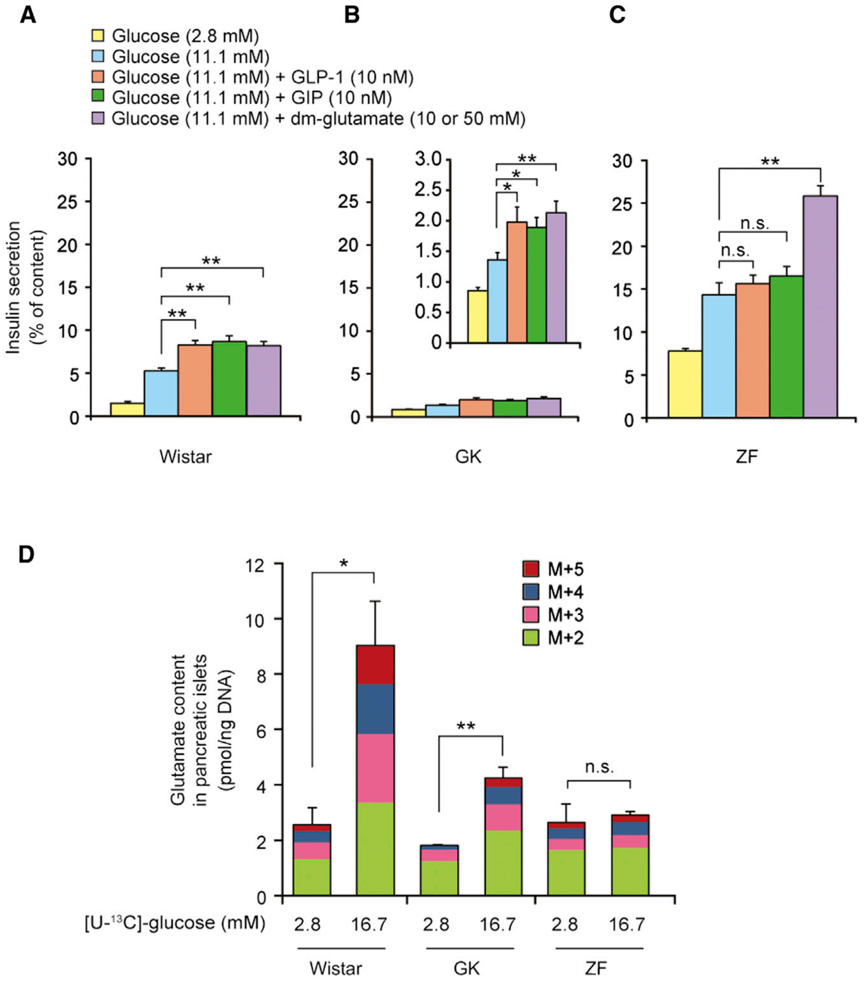

Figure 6. Pathophysiological Role of Glutamate in Insulin Secretion

(A–C) Insulin secretion from pancreatic islets of Wistar (A, n = 8 for each), GK (B, n = 6 for each), and ZF (C, n = 8 for each) rats. The inset in the middle panel (B) is included to magnify the scale of the *y* axis in GK rats. The concentrations of dm-glutamate used were 10 mM, 10 mM, and 50 mM for Wistar, GK, and ZF rats, respectively.

(D) Production of glutamate isotopomers (M+2 to M+5) by glucose stimulation in pancreatic islets of Wistar, GK, and ZF rats (n = 3 for each).

The data are expressed as means ± SEM. Results are representative of three independent experiments. Dunnett's method (A–C) and Welch's t test (D) were used for evaluation of statistical significance. $^{*}p < 0.05$; $^{**}p < 0.01$; n.s., not significant. See also Figure S6.

secretion was already high compared with the control, but significant GIIS occurred (Figures 6C and S6B). Unlike Wistar and GK rats, adult ZF rats (≥12 weeks of age) exhibited no amplification of insulin secretion in response to incretin stimulation, but dimethyl-glutamate remained effective. We then examined whether glucose-induced islet glutamate production is affected in these rat models, as assessed by analysis of ^{13}C-enriched glutamate. Glucose stimulated glutamate production in GK islets, but the levels were lower than those in Wistar islets (Figure 6D). By contrast, no glucose-stimulated glutamate production was seen in ZF islets.

DISCUSSION

Using metabolome analysis by mass spectrometry, we found that glucose increased cytosolic glutamate through the malate-aspartate shuttle and that GLP-1 promoted glutamate transport into insulin granules via cAMP/PKA signaling. We also found that glutamate in insulin granules stimulated insulin secretion, as assessed by capacitance measurements, TIRFM analysis, and VGLUT1 knockout and KD experiments. Glutamate thus acts as a key cell signal linking glucose metabolism to incretin/cAMP action to amplify insulin secretion. Figure 7 summarizes these findings schematically.

It is well established that incretins and agents that elevate intracellular cAMP amplify insulin secretion but are unable to initiate insulin secretion on their own. The amplifying effect is not mediated by enhanced glucose metabolism, i.e., increased ATP production (Brisson et al., 1972; Peyot et al., 2009), and the nature of the molecular link between glucose metabolism and incretin/cAMP signaling in amplification of insulin secretion remains elusive. To clarify this, we performed a metabolome analysis using capillary electrophoresis-mass spectrometry (CE-MS), which can detect and quantify metabolites, including the intermediate metabolites in glycolysis and the TCA cycle, amino acids, and nucleic acids. Based on our findings that GLP-1 has little or no effect on the amounts of metabolites in glucose metabolism (glycolysis, TCA cycle, pentose phosphate pathway, glycogenesis, and NADH shuttles) or the activities of enzymes in the malate-aspartate shuttle, we can discard the possibility that incretin/cAMP signaling exerts a direct effect on glucose metabolism.

Using differential metabolomics, we compared metabolites associated with glucose metabolism between incretin-responsive and -unresponsive β cell lines. Based on the finding that inhibition of the malate-aspartate shuttle blocked incretin-induced insulin secretion, we focused on the metabolites derived from the shuttle. Among them, glutamate attracted our attention. Mitochondrial glutamate (produced by GDH) was previously proposed to act as a signal in GIIS (Maechler and Wollheim, 1999; Høy et al., 2002; Casimir et al., 2009), although this has been controversial and some observations seem inconsistent with the notion (Bertrand et al., 2002; MacDonald and Fahien, 2000). In the present study, we found that 72% KD of GDH1 (at the mRNA level) did not affect GIIS in MIN6-K8 cells. However,

OPEN ACCESS
CellPress

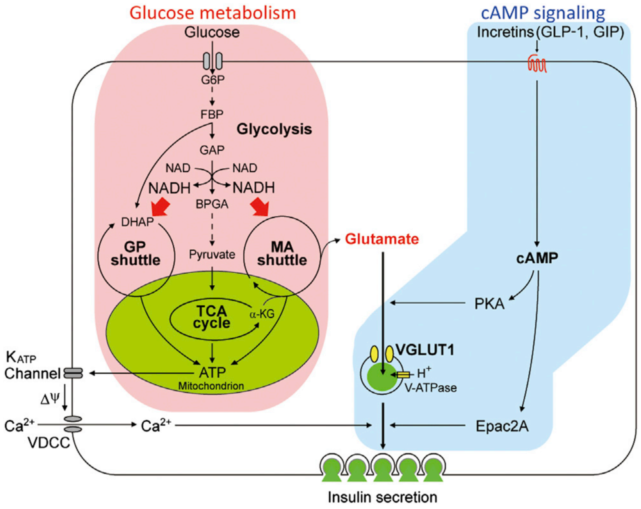

Figure 7. Glutamate Acts as a Key Signal in Amplification of Insulin Secretion by Incretin/cAMP Signaling

Glutamate links glucose metabolism and incretin/cAMP signaling to amplify insulin secretion. See text for the details. GP shuttle, glycerol phosphate shuttle; MA shuttle, malate-aspartate shuttle; K_{ATP} channel, ATP-sensitive K^+ channel; VDCC, voltage-dependent Ca^{2+} channel.

we cannot rule out the possibility that mitochondrial glutamate is involved in GIIS, since knockout of GDH1 in mice decreased GIIS by ~50% compared with control (Carobbio et al., 2009; Vetterli et al., 2012). On the other hand, the role of cellular glutamate in amplification of insulin secretion by incretin/cAMP signaling has not been investigated. We demonstrate here that cytosolic glutamate derived from the malate-aspartate shuttle, which represents a major fraction of cellular glutamate produced by glucose, is crucial for incretin-induced insulin secretion. Mitochondrial glutamate may also contribute to a fraction of cytosolic glutamate, as it is continuously exported to cytosol through GC1 (Casimir et al., 2009).

Our findings indicate that glutamate transport into the insulin granules occurs only in cells exposed to the combination of glucose and GLP-1, and not in cells exposed to glucose alone. Indeed, inhibition of glutamate transport into the granules diminished incretin-induced insulin secretion, but did not affect GIIS. Our data demonstrate that glutamate transport into insulin granules by cAMP signaling plays a decisive role in incretin-induced insulin secretion. Although glucose induces cAMP production (Charles et al., 1973; Grill and Cerasi, 1973; Dyachok et al., 2008), it produces a much smaller amount of cAMP than does GLP-1 (Delmeire et al., 2003; Iwasaki et al., 2010; Susini et al., 1998). Thus, glucose-induced cAMP production is insufficient (at least under the experimental conditions used in the present study) to sustain sufficient glutamate transport into the insulin granules, but under other experimental paradigms, this could occur.

In neurons, glutamate transport into the synaptic vesicles through VGLUTs is regulated by the electrical potential ($\Delta\varphi$) and pH gradient (ΔpH) across the vesicular membrane (Omote et al., 2011). A proton pump V-ATPase on insulin granules contributes to generation of $\Delta\varphi$ and ΔpH across the insulin granule membrane (Aspinwall et al., 1997), in which Cl^- flux is needed (Xie et al., 1983; Xie et al., 1989). The Cl^- flux through the chloride transport protein ClC-3 in the insulin granule membrane has been shown to be required for GIIS (Li et al., 2009). In addition, it was reported that glutamate flux in the insulin granules was involved in the generation of $\Delta\varphi$ and ΔpH, and that perturbing the flux decreased insulin granule exocytosis (Gammelsaeter et al., 2011). We found that inhibition of either VGLUT1 or V-ATPase did not affect GIIS, but reduced amplification of insulin secretion by incretins. Furthermore, dimethyl-glutamate was able to mimic the effects of incretins under the condition of VGLUT1 deficiency or V-ATPase inhibition. These results indicate that glutamate transport into insulin granules, which is mediated by activation of cAMP/PKA signaling, is required for amplification of insulin granule exocytosis. Although the precise mechanism by which glutamate in insulin granules stimulates insulin granule exocytosis remains to be elucidated, the finding by TIRFM analysis that most of the insulin granule exocytosis induced by dimethyl-glutamate is caused by granules that are newly recruited and immediately fused to the plasma membrane (Shibasaki et al., 2007) suggests that glutamate in insulin granules facilitates recruitment toward and/or fusion of the insulin granules with the plasma membrane.

We also provide data implicating defective glutamate signaling in the pathophysiology of insulin secretion in the GK and ZF rat models of human diabetes and obesity, respectively. In GK rats, amplification of insulin secretion by incretins was much reduced, with a marked suppression of glutamate production by glucose. In ZF rats, there was no amplification at all by incretins, with no glutamate production. Importantly, dimethyl-glutamate was able to amplify insulin secretion, mimicking the effect of incretins, in all of these models. Thus, impaired production of glutamate in pancreatic β cells could lead to a defect in incretin-induced insulin secretion. Indeed, some reports have suggested that incretin-based therapies have limited insulinotropic effects in clinical settings (Holst et al., 2011; Kubota et al., 2012).

In summary, our data demonstrate that glutamate acts as a key signal linking glucose metabolism and incretin/cAMP action to amplify insulin secretion. Therefore, elucidating glutamate signaling may not only clarify the pathophysiology of diabetes mellitus but may also pave the way for novel therapeutic strategies.

EXPERIMENTAL PROCEDURES

Animal Experiments

Male Wistar (Slc:Wistar), GK (GK/Slc), and ZF (Slc:Zucker, *fa/fa*) rats (10 weeks old) were purchased from Japan SLC. VGLUT1 knockout (*Slc17a7*$^{-/-}$) mice were provided by R.H. Edwards. Animal experiments were approved by the Committee on Animal Experimentation of Kobe University and carried out in accordance with the Guidelines for Animal Experimentation at Kobe University. Electrophysiological experiments were performed on β cells from NMRI mice and the experiments were conducted in accordance with the Animals (Scientific Procedures) Act 1986 and ethical guidelines of the University of Alberta and University of Oxford.

Metabolome Analysis

Hydrophilic metabolites were extracted from MIN6-K cells and then subjected to CE-MS. See Supplemental Experimental Procedures for details.

Insulin Secretion

Insulin-secretion experiments on MIN6-K cells and pancreatic islets were performed as previously described (Yasuda et al., 2010). See Supplemental Experimental Procedures for details.

KD Experiments

MIN6-K8 cells were infected with small interfering RNA (siRNA) or adenovirus carrying short hairpin RNA (shRNA). See Supplemental Experimental Procedures for details.

mRNA Expression

Isolation of total RNA and quantitative real-time RT-PCR were performed as previously described (Matsubara et al., 2012), with 18S ribosomal RNA used as an internal control.

Glutamate Contents

Glutamate contents in lysed cells were determined with the use of the L-Glutamate Assay Kit II (Yamasa). The contents of glutamate isotopomers were also measured by ^{13}C-enrichment analysis, with uniformly labeled [U-^{13}C]-glucose as a substrate, using CE-MS. See Supplemental Experimental Procedures for details.

Electrophysiology

Capacitance recordings of single β cell exocytosis were obtained essentially as described previously (Kanno et al., 2004). See Supplemental Experimental Procedures for details.

Immunofluorescence Staining

Immunofluorescence staining was performed as previously described (Yasuda et al., 2010). See Supplemental Experimental Procedures for details.

TIRFM Analysis

TIRFM analysis was performed as previously described (Shibasaki et al., 2007).

Statistical Analysis

The data are expressed as means ± SEM. Statistical comparisons were made using Welch's t test, Student's t test, Dunnett's method, and the Tukey-Kramer method as indicated in the figure legends. Differences for which the p value was <0.05 were regarded as statistically significant. Hierarchical cluster analysis (average linkage) was performed on Pareto-scaled metabolomics data (distance measures based on the Pearson correlation) using Multiple Experiment Viewer (MeV) (Saeed et al., 2003).

SUPPLEMENTAL INFORMATION

Supplemental Information includes Supplemental Experimental Procedures, six figures, and two tables and can be found with this article online at http://dx.doi.org/10.1016/j.celrep.2014.09.030.

AUTHOR CONTRIBUTIONS

S.S. conceived the project. G.G., M.O., M.I., N.Y., K.M., T.S., N.I., T.B., A.M., E.F., P.R., and S.S. contributed to the study design and data analyses. G.G., M.O., M.I., N.Y., K.M., Y.N., K.H., B.H., X.W., H.T., K.K., T.M., R.H., N.H., K.S., and A.M. performed the experiments and collected the data. N.Y., K.M., T.S., E.F., P.R., and S.S. supervised the work. All authors participated in discussion of the results. G.G., M.O., M.I., N.Y., and S.S. wrote the manuscript with feedback from all other authors, including significant contributions from K.M., T.S., H.T., and P.R.

ACKNOWLEDGMENTS

We thank D. Accili, F.M. Ashcroft, and G.I. Bell for critical readings of the manuscript and insightful suggestions. We also thank R.H. Edwards and Y. Moriyama for providing VGLUT1 knockout mice and anti-VGLUT1 antibody, respectively, and S. Uehara and T.N. Haase for help with animal and knockdown experiments. We are grateful to M. Iizuka, S. Hidaka, H. Kitanoya, M. Hashim, C. Seki, and T. Yamaguchi for their technical assistance, and G.K. Honkawa for assistance in preparing the manuscript. This study was supported by a CREST grant from the Japan Science and Technology Agency; Grants-in-Aid for Scientific Research from the Ministry of Education, Culture, Sport, Science and Technology, Japan; the Wellcome Trust; and the Canada Institutes of Health Research. This study was also supported in part by research grants from MSD K.K. and Novo Nordisk Pharma.

Received: February 13, 2014
Revised: August 19, 2014
Accepted: September 15, 2014
Published: October 16, 2014

REFERENCES

Ahrén, B. (2009). Islet G protein-coupled receptors as potential targets for treatment of type 2 diabetes. Nat. Rev. Drug Discov. *8*, 369–385.

Aspinwall, C.A., Brooks, S.A., Kennedy, R.T., and Lakey, J.R. (1997). Effects of intravesicular H^+ and extracellular H^+ and Zn^{2+} on insulin secretion in pancreatic beta cells. J. Biol. Chem. *272*, 31308–31314.

Bai, L., Zhang, X., and Ghishan, F.K. (2003). Characterization of vesicular glutamate transporter in pancreatic α - and β -cells and its regulation by glucose. Am. J. Physiol. Gastrointest. Liver Physiol. *284*, G808–G814.

Bellocchio, E.E., Reimer, R.J., Fremeau, R.T., Jr., and Edwards, R.H. (2000). Uptake of glutamate into synaptic vesicles by an inorganic phosphate transporter. Science *289*, 957–960.

Bertrand, G., Ishiyama, N., Nenquin, M., Ravier, M.A., and Henquin, J.C. (2002). The elevation of glutamate content and the amplification of insulin secretion in glucose-stimulated pancreatic islets are not causally related. J. Biol. Chem. *277*, 32883–32891.

Bos, J.L. (2006). Epac proteins: multi-purpose cAMP targets. Trends Biochem. Sci. *31*, 680–686.

Brisson, G.R., Malaisse-Lagae, F., and Malaisse, W.J. (1972). The stimulus-secretion coupling of glucose-induced insulin release. VII. A proposed site of action for adenosine-3′,5′-cyclic monophosphate. J. Clin. Invest. *51*, 232–241.

Brozzi, F., Lajus, S., Diraison, F., Rajatileka, S., Hayward, K., Regazzi, R., Molnár, E., and Váradi, A. (2012). MyRIP interaction with MyoVa on secretory granules is controlled by the cAMP-PKA pathway. Mol. Biol. Cell *23*, 4444–4455.

Carobbio, S., Frigerio, F., Rubi, B., Vetterli, L., Bloksgaard, M., Gjinovci, A., Pournourmohammadi, S., Herrera, P.L., Reith, W., Mandrup, S., and Maechler, P. (2009). Deletion of glutamate dehydrogenase in beta-cells abolishes part of the insulin secretory response not required for glucose homeostasis. J. Biol. Chem. *284*, 921–929.

Casimir, M., Lasorsa, F.M., Rubi, B., Caille, D., Palmieri, F., Meda, P., and Maechler, P. (2009). Mitochondrial glutamate carrier GC1 as a newly identified player in the control of glucose-stimulated insulin secretion. J. Biol. Chem. *284*, 25004–25014.

Cataland, S., Crockett, S.E., Brown, J.C., and Mazzaferri, E.L. (1974). Gastric inhibitory polypeptide (GIP) stimulation by oral glucose in man. J. Clin. Endocrinol. Metab. *39*, 223–228.

Charles, M.A., Fanska, R., Schmid, F.G., Forsham, P.H., and Grodsky, G.M. (1973). Adenosine 3′,5′-monophosphate in pancreatic islets: glucose-induced insulin release. Science *179*, 569–571.

Delmeire, D., Flamez, D., Hinke, S.A., Cali, J.J., Pipeleers, D., and Schuit, F. (2003). Type VIII adenylyl cyclase in rat beta cells: coincidence signal detector/generator for glucose and GLP-1. Diabetologia *46*, 1383–1393.

Drucker, D.J. (2006). The biology of incretin hormones. Cell Metab. *3*, 153–165.

Drucker, D.J., and Nauck, M.A. (2006). The incretin system: glucagon-like peptide-1 receptor agonists and dipeptidyl peptidase-4 inhibitors in type 2 diabetes. Lancet *368*, 1696–1705.

OPEN
ACCESS
CellPress

Dyachok, O., Idevall-Hagren, O., Sågetorp, J., Tian, G., Wuttke, A., Arrieumerlou, C., Akusjärvi, G., Gylfe, E., and Tengholm, A. (2008). Glucose-induced cyclic AMP oscillations regulate pulsatile insulin secretion. Cell Metab. *8*, 26–37.

Eliasson, L., Ma, X., Renström, E., Barg, S., Berggren, P.O., Galvanovskis, J., Gromada, J., Jing, X., Lundquist, I., Salehi, A., et al. (2003). SUR1 regulates PKA-independent cAMP-induced granule priming in mouse pancreatic B-cells. J. Gen. Physiol. *121*, 181–197.

Eto, K., Tsubamoto, Y., Terauchi, Y., Sugiyama, T., Kishimoto, T., Takahashi, N., Yamauchi, N., Kubota, N., Murayama, S., Aizawa, T., et al. (1999). Role of NADH shuttle system in glucose-induced activation of mitochondrial metabolism and insulin secretion. Science *283*, 981–985.

Gammelsaeter, R., Coppola, T., Marcaggi, P., Storm-Mathisen, J., Chaudhry, F.A., Attwell, D., Regazzi, R., and Gundersen, V. (2011). A role for glutamate transporters in the regulation of insulin secretion. PLoS ONE *6*, e22960.

Goto, Y., Kakizaki, M., and Masaki, N. (1975). Spontaneous diabetes produced by selective breeding of normal Wistar rats. Proc. Jpn. Acad. *51*, 80–85.

Gras, C., Herzog, E., Bellenchi, G.C., Bernard, V., Ravassard, P., Pohl, M., Gasnier, B., Giros, B., and El Mestikawy, S. (2002). A third vesicular glutamate transporter expressed by cholinergic and serotoninergic neurons. J. Neurosci. *22*, 5442–5451.

Grill, V., and Cerasi, E. (1973). Activation by glucose of adenyl cyclase in pancreatic islets of the rat. FEBS Lett. *33*, 311–314.

Henquin, J.C. (2000). Triggering and amplifying pathways of regulation of insulin secretion by glucose. Diabetes *49*, 1751–1760.

Holst, J.J. (2007). The physiology of glucagon-like peptide 1. Physiol. Rev. *87*, 1409–1439.

Holst, J.J., Knop, F.K., Vilsbøll, T., Krarup, T., and Madsbad, S. (2011). Loss of incretin effect is a specific, important, and early characteristic of type 2 diabetes. Diabetes Care *34* (*Suppl 2*), S251–S257.

Høy, M., Maechler, P., Efanov, A.M., Wollheim, C.B., Berggren, P.O., and Gromada, J. (2002). Increase in cellular glutamate levels stimulates exocytosis in pancreatic beta-cells. FEBS Lett. *531*, 199–203.

Iwasaki, M., Minami, K., Shibasaki, T., Miki, T., Miyazaki, J., and Seino, S. (2010). Establishment of new clonal pancreatic β-cell lines (MIN6-K) useful for study of incretin/cyclic adenosine monophosphate signaling. J. Diabetes Investig. *1*, 137–142.

Kanno, T., Ma, X., Barg, S., Eliasson, L., Galvanovskis, J., Göpel, S., Larsson, M., Renström, E., and Rorsman, P. (2004). Large dense-core vesicle exocytosis in pancreatic beta-cells monitored by capacitance measurements. Methods *33*, 302–311.

Kreymann, B., Williams, G., Ghatei, M.A., and Bloom, S.R. (1987). Glucagon-like peptide-1 7-36: a physiological incretin in man. Lancet *2*, 1300–1304.

Kubota, A., Maeda, H., Kanamori, A., Matoba, K., Jin, Y., Minagawa, F., Obana, M., Iemitsu, K., Ito, S., Amamiya, H., et al. (2012). Efficacy and safety of sitagliptin monotherapy and combination therapy in Japanese type 2 diabetes patients. J. Diabetes Investig. *3*, 503–509.

Li, D.Q., Jing, X., Salehi, A., Collins, S.C., Hoppa, M.B., Rosengren, A.H., Zhang, E., Lundquist, I., Olofsson, C.S., Mörgelin, M., et al. (2009). Suppression of sulfonylurea- and glucose-induced insulin secretion in vitro and in vivo in mice lacking the chloride transport protein ClC-3. Cell Metab. *10*, 309–315.

MacDonald, M.J. (1982). Evidence for the malate aspartate shuttle in pancreatic islets. Arch. Biochem. Biophys. *213*, 643–649.

MacDonald, M.J., and Fahien, L.A. (2000). Glutamate is not a messenger in insulin secretion. J. Biol. Chem. *275*, 34025–34027.

Maechler, P., and Wollheim, C.B. (1999). Mitochondrial glutamate acts as a messenger in glucose-induced insulin exocytosis. Nature *402*, 685–689.

Matsubara, T., Mita, A., Minami, K., Hosooka, T., Kitazawa, S., Takahashi, K., Tamori, Y., Yokoi, N., Watanabe, M., Matsuo, E., et al. (2012). PGRN is a key adipokine mediating high fat diet-induced insulin resistance and obesity through IL-6 in adipose tissue. Cell Metab. *15*, 38–50.

Naito, S., and Ueda, T. (1983). Adenosine triphosphate-dependent uptake of glutamate into protein I-associated synaptic vesicles. J. Biol. Chem. *258*, 696–699.

Nauck, M.A., Heimesaat, M.M., Orskov, C., Holst, J.J., Ebert, R., and Creutzfeldt, W. (1993). Preserved incretin activity of glucagon-like peptide 1 [7-36 amide] but not of synthetic human gastric inhibitory polypeptide in patients with type-2 diabetes mellitus. J. Clin. Invest. *91*, 301–307.

Omote, H., Miyaji, T., Juge, N., and Moriyama, Y. (2011). Vesicular neurotransmitter transporter: bioenergetics and regulation of glutamate transport. Biochemistry *50*, 5558–5565.

Ostenson, C.G., Khan, A., Abdel-Halim, S.M., Guenifi, A., Suzuki, K., Goto, Y., and Efendic, S. (1993). Abnormal insulin secretion and glucose metabolism in pancreatic islets from the spontaneously diabetic GK rat. Diabetologia *36*, 3–8.

Peyot, M.L., Gray, J.P., Lamontagne, J., Smith, P.J., Holz, G.G., Madiraju, S.R., Prentki, M., and Heart, E. (2009). Glucagon-like peptide-1 induced signaling and insulin secretion do not drive fuel and energy metabolism in primary rodent pancreatic β-cells. PLoS ONE *4*, e6221.

Polonsky, K.S., Given, B.D., Hirsch, L.J., Tillil, H., Shapiro, E.T., Beebe, C., Frank, B.H., Galloway, J.A., and Van Cauter, E. (1988). Abnormal patterns of insulin secretion in non-insulin-dependent diabetes mellitus. N. Engl. J. Med. *318*, 1231–1239.

Porte, D., Jr. (1991). Banting lecture 1990. Beta-cells in type II diabetes mellitus. Diabetes *40*, 166–180.

Prentki, M., and Matschinsky, F.M. (1987). Ca^{2+}, cAMP, and phospholipid-derived messengers in coupling mechanisms of insulin secretion. Physiol. Rev. *67*, 1185–1248.

Rorsman, P., and Renström, E. (2003). Insulin granule dynamics in pancreatic beta cells. Diabetologia *46*, 1029–1045.

Roseth, S., Fykse, E.M., and Fonnum, F. (1995). Uptake of L-glutamate into rat brain synaptic vesicles: effect of inhibitors that bind specifically to the glutamate transporter. J. Neurochem. *65*, 96–103.

Saeed, A.I., Sharov, V., White, J., Li, J., Liang, W., Bhagabati, N., Braisted, J., Klapa, M., Currier, T., Thiagarajan, M., et al. (2003). TM4: a free, open-source system for microarray data management and analysis. Biotechniques *34*, 374–378.

Seino, S., and Shibasaki, T. (2005). PKA-dependent and PKA-independent pathways for cAMP-regulated exocytosis. Physiol. Rev. *85*, 1303–1342.

Seino, Y., Fukushima, M., and Yabe, D. (2010). GIP and GLP-1, the two incretin hormones: similarities and differences. J. Diabetes Investig. *1*, 8–23.

Seino, S., Shibasaki, T., and Minami, K. (2011). Dynamics of insulin secretion and the clinical implications for obesity and diabetes. J. Clin. Invest. *121*, 2118–2125.

Shibasaki, T., Takahashi, H., Miki, T., Sunaga, Y., Matsumura, K., Yamanaka, M., Zhang, C., Tamamoto, A., Satoh, T., Miyazaki, J., and Seino, S. (2007). Essential role of Epac2/Rap1 signaling in regulation of insulin granule dynamics by cAMP. Proc. Natl. Acad. Sci. USA *104*, 19333–19338.

Siegel, E.G., and Creutzfeldt, W. (1985). Stimulation of insulin release in isolated rat islets by GIP in physiological concentrations and its relation to islet cyclic AMP content. Diabetologia *28*, 857–861.

Song, W.J., Seshadri, M., Ashraf, U., Mdluli, T., Mondal, P., Keil, M., Azevedo, M., Kirschner, L.S., Stratakis, C.A., and Hussain, M.A. (2011). Snapin mediates incretin action and augments glucose-dependent insulin secretion. Cell Metab. *13*, 308–319.

Sugawara, K., Shibasaki, T., Mizoguchi, A., Saito, T., and Seino, S. (2009). Rab11 and its effector Rip11 participate in regulation of insulin granule exocytosis. Genes Cells *14*, 445–456.

Susini, S., Roche, E., Prentki, M., and Schlegel, W. (1998). Glucose and glucoincretin peptides synergize to induce c-fos, c-jun, junB, zif-268, and nur-77 gene expression in pancreatic β(INS-1) cells. FASEB J. *12*, 1173–1182.

Takamori, S., Rhee, J.S., Rosenmund, C., and Jahn, R. (2001). Identification of differentiation-associated brain-specific phosphate transporter as a second vesicular glutamate transporter (VGLUT2). J. Neurosci. *21*, RC182.

Uehara, S., Jung, S.K., Morimoto, R., Arioka, S., Miyaji, T., Juge, N., Hiasa, M., Shimizu, K., Ishimura, A., Otsuka, M., et al. (2006). Vesicular storage and secretion of L-glutamate from glucagon-like peptide 1-secreting clonal intestinal L cells. J. Neurochem. *96*, 550–560.

Vetterli, L., Carobbio, S., Pournourmohammadi, S., Martin-Del-Rio, R., Skytt, D.M., Waagepetersen, H.S., Tamarit-Rodriguez, J., and Maechler, P. (2012). Delineation of glutamate pathways and secretory responses in pancreatic islets with β-cell-specific abrogation of the glutamate dehydrogenase. Mol. Biol. Cell *23*, 3851–3862.

Weir, G.C., Mojsov, S., Hendrick, G.K., and Habener, J.F. (1989). Glucagonlike peptide I (7-37) actions on endocrine pancreas. Diabetes *38*, 338–342.

Xie, X.S., Stone, D.K., and Racker, E. (1983). Determinants of clathrin-coated vesicle acidification. J. Biol. Chem. *258*, 14834–14838.

Xie, X.S., Crider, B.P., and Stone, D.K. (1989). Isolation and reconstitution of the chloride transporter of clathrin-coated vesicles. J. Biol. Chem. *264*, 18870–18873.

Yasuda, T., Shibasaki, T., Minami, K., Takahashi, H., Mizoguchi, A., Uriu, Y., Numata, T., Mori, Y., Miyazaki, J., Miki, T., and Seino, S. (2010). Rim2α determines docking and priming states in insulin granule exocytosis. Cell Metab. *12*, 117–129.

Zucker, L.M., and Zucker, T.F. (1961). Fatty, a new mutation in the rat. J. Hered. *52*, 275–278.

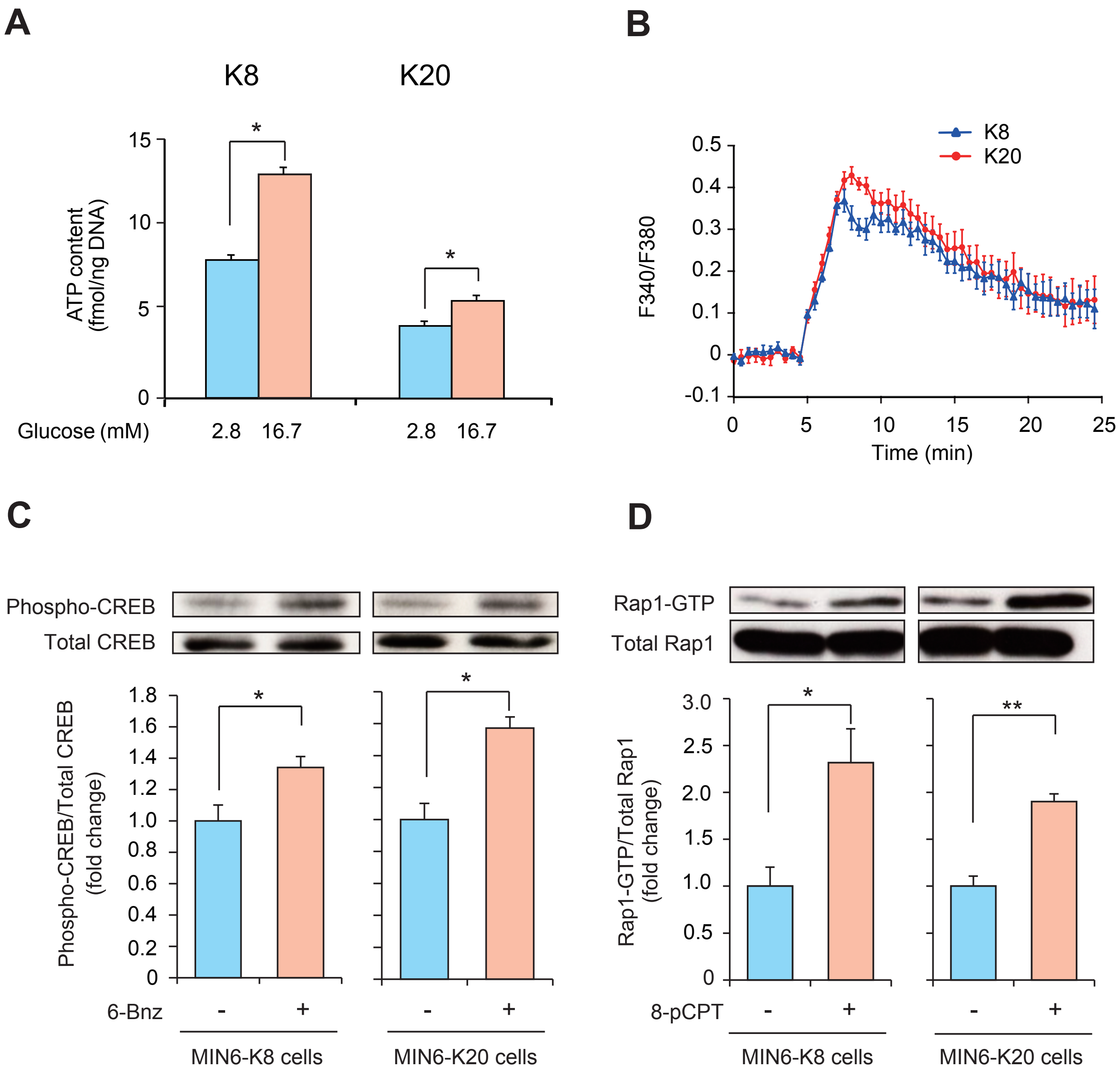

Figure S1. Comparison of ATP Content, Intracellular Ca^{2+} Concentration, and Activities of Downstream Targets of cAMP between MIN6-K8 and-K20 Cells, related to Figure 1

(A) ATP contents in MIN6-cells treated with glucose at low (2.8 mM) or high (16.7 mM) concentrations (n = 8 for each).

(B) Changes in intracellular Ca^{2+} concentration in MIN6-K cells treated with glucose at high (16.7 mM) concentration (n = 6 for each).

(C) Phosphorylation of CREB in MIN6-K cells treated with or without 30 μM 6-Bnz-cAMP-AM (6-Bnz), a PKA-selective cAMP analog, under the basal condition (2.8 mM glucose) (n = 10 for each).

(D) Activation of Rap1 in MIN6-K cells treated with or without 5 μM 8-pCPT-2'-*O*-Me-cAMP-AM (8-pCPT), an Epac-selective cAMP analog, under the basal condition (2.8 mM glucose) (n = 6 for each).

The data are expressed as means ± SEM. Results are representative of 3 independent experiments. Welch's t test was used for evaluation of statistical significance. *$p < 0.05$, **$p < 0.01$. A representative blot for each experiment is shown (C and D). Quantification of autoradiography is shown with corresponding bars positioned under the bands. The intensity of the phosphorylated-CREB and the Rap1-GTP signal was normalized by that of total CREB and Rap1, respectively. Phosphorylated-CREB and Rap1-GTP in the cells treated with analogs are presented as relative to those without analogs, which are considered as 1.

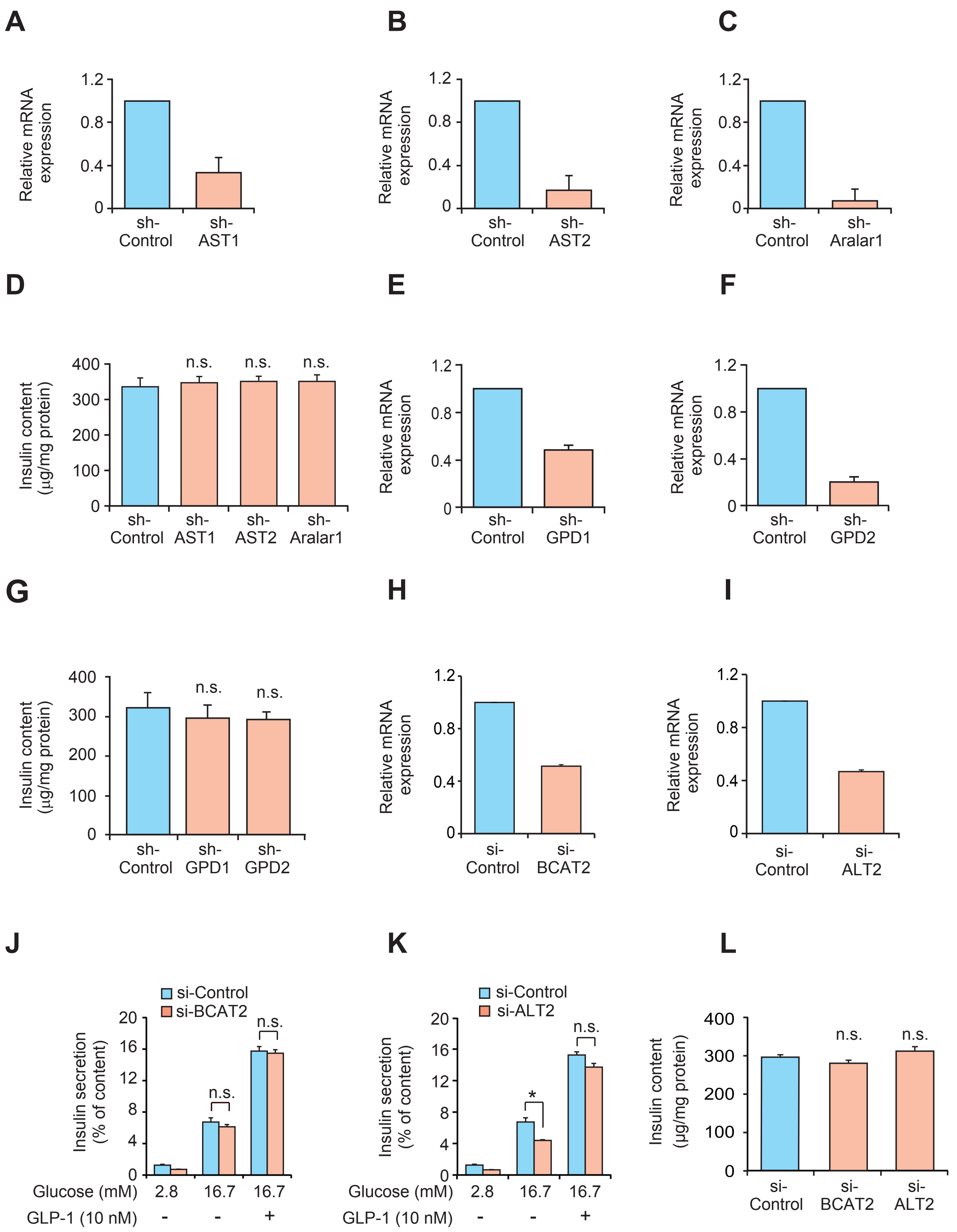
A
Relative mRNA expression
1.2
0.8
0.4
0
sh-Control
sh-AST1
B
Relative mRNA expression
sh-Control
sh-AST2
C
Relative mRNA expression
sh-Control
sh-Aralar1
D
Insulin content (µg/mg protein)
400
300
200
100
n.s.
sh-Control
sh-AST1
sh-AST2
sh-Aralar1
E
Relative mRNA expression
sh-Control
sh-GPD1
F
Relative mRNA expression
sh-Control
sh-GPD2
G
Insulin content (µg/mg protein)
n.s.
sh-Control
sh-GPD1
sh-GPD2
H
Relative mRNA expression
si-Control
si-BCAT2
I
Relative mRNA expression
si-Control
si-ALT2
J
si-Control
si-BCAT2
Insulin secretion (% of content)
20
16
12
8
4
n.s.
Glucose (mM) 2.8 16.7 16.7
GLP-1 (10 nM) - - +
K
si-Control
si-ALT2
Insulin secretion (% of content)
*
n.s.
Glucose (mM) 2.8 16.7 16.7
GLP-1 (10 nM) - - +
L
Insulin content (µg/mg protein)
n.s.
si-Control
si-BCAT2
si-ALT2

Figure S2. Effect of Knockdown (KD) of AST1, AST2, Aralar1, GPD1, GPD2, BCAT2, or ALT2 on mRNA Expression, Insulin Secretion, and Insulin Content in MIN6-K8 Cells, related to Figure 2

(A-C) Relative mRNA expression levels of AST1 (A), AST2 (B), and Aralar1 (C) (n = 3 for each).

(D) Effect of KD of AST1, AST2, or Aralar1 on insulin content (n = 4-8 for each).

(E and F) Relative mRNA expression levels of GPD1 (E) and GPD2 (F) (n = 3 for each).

(G) Effect of KD of GPD1 or GPD2 on insulin content (n = 4-8 for each).

(H and I) Relative mRNA expression levels of BCAT2 (H) and ALT2 (I) (n = 3 for each).

(J and K) Effect of KD of BCAT2 (J) or ALT2 (K) on insulin secretion (n = 4-8 for each).

(L) Effect of KD of BCAT2 or ALT2 on insulin content (n = 4-8 for each).

The data are expressed as means ± SEM. Results are representative of 3 independent experiments. mRNA expression levels in the respective KD cells are presented as relative to those in sh- or si-Control cells, which are considered as 1. Dunnett's method was used for evaluation of statistical significance versus sh- or si-Control (D, G, and L). Welch’s t test was used for evaluation of statistical significance (J and K). *$p < 0.05$; n.s., not significant.

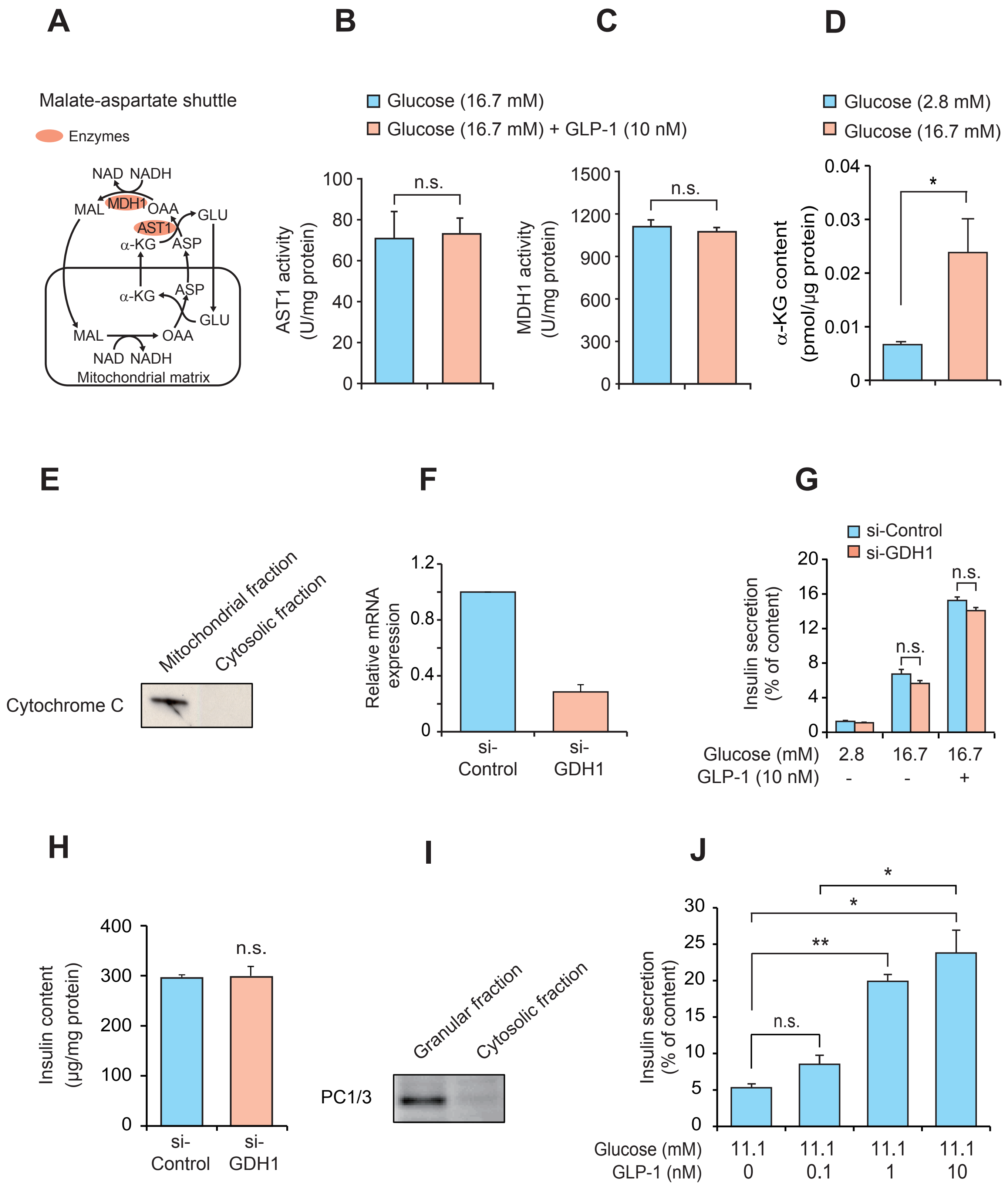

A
Malate-aspartate shuttle
Enzymes
NAD NADH
MAL MDH1 OAA
AST1
GLU
α-KG ASP
α-KG ASP
GLU
MAL OAA
NAD NADH
Mitochondrial matrix
B
Glucose (16.7 mM)
Glucose (16.7 mM) + GLP-1 (10 nM)
AST1 activity (U/mg protein)
n.s.
C
MDH1 activity (U/mg protein)
n.s.
D
Glucose (2.8 mM)
Glucose (16.7 mM)
α-KG content (pmol/μg protein)
*
E
Mitochondrial fraction
Cytosolic fraction
Cytochrome C
F
Relative mRNA expression
si-Control
si-GDH1
G
si-Control
si-GDH1
Insulin secretion (% of content)
n.s.
n.s.
Glucose (mM) 2.8 16.7 16.7
GLP-1 (10 nM) - - +
H
Insulin content (μg/mg protein)
n.s.
si-Control
si-GDH1
I
Granular fraction
Cytosolic fraction
PC1/3
J
Insulin secretion (% of content)
*
*
**
n.s.
Glucose (mM) 11.1 11.1 11.1 11.1
GLP-1 (nM) 0 0.1 1 10

Figure S3. Effect of GLP-1 on Activity of AST1 or MDH1, Effect of glucose on α-ketoglutarate content, Effect of GDH1 KD on Insulin Secretion, and Dose-dependent Effect of GLP-1 on Insulin Secretion in MIN6-K8 Cells, related to Figure 3

(A) Malate-aspartate shuttle. AST1, aspartate aminotransferase 1; MDH1, malate dehydrogenase 1. See also the legend to Figure 1E.

(B and C) Effects of GLP-1 on activities of AST1 (B) and MDH1 (C) (n = 4 for each).

(D) Effect of glucose on total cellular α-ketoglutarate contents in MIN6-K8 cells (n = 3 for each).

(E) Mitochondrial fraction was confirmed by immunoblot analysis using the antibody against cytochrome C, a marker for mitochondria.

(F) Relative mRNA expression levels of GDH1 (n = 3 for each).

(G) Effect of KD of GDH1 on insulin secretion (n = 4-8 for each).

(H) Effect of KD of GDH1 on insulin content (n = 4-8 for each).

(I) Granular fraction was confirmed by immunoblot analysis using the antibody against PC1/3, a marker of insulin granules.

(J) Dose-dependent effects of GLP-1 on insulin secretion under the glucose (11.1 mM)-stimulated condition (n = 4-8 for each).

The data are expressed as means ± SEM. Results are representative of 3 independent experiments. mRNA expression levels in the respective KD cells are presented as relative to those in si-Control cells, which are considered as 1. Welch's t test (B-D, G, and H) or Tukey-Kramer method (J) were used for evaluation of statistical significance. *$p < 0.05$; **$p < 0.01$; n.s., not significant.

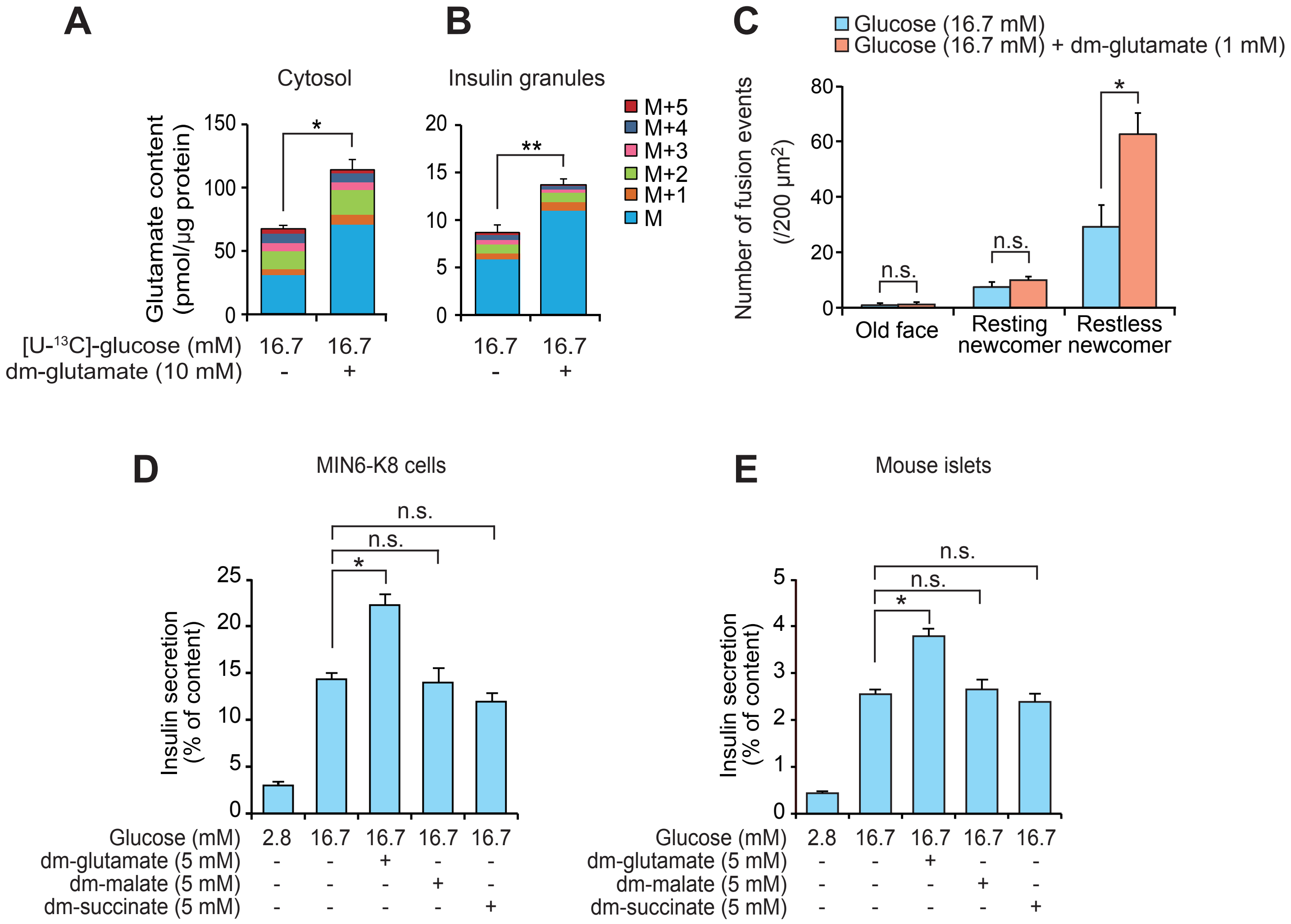

Figure S4. Effects of Dimethyl-Glutamate on Glutamate Contents in the Cytosol and Insulin Granules and on Dynamics of Insulin Granule Exocytosis, and Effect of Dimethyl-Glutamate, -Malate, or -Succinate on Insulin Secretion, related to Figure 4

(A and B) Effect of dimethyl-glutamate on glutamate contents in the cytosol (A) and insulin granules (B) in MIN6-K8 cells (n = 3-6 for each). Changes in contents of glutamate isotopomers (M to M+5) in the cytosol (A) and insulin granules (B) under the glucose-stimulated condition in the absence or presence of dimethyl-glutamate (dm-glutamate) were assessed by analysis of ^{13}C-enriched glutamate (n = 3-6 for each). Treatment of MIN6-K8 cells with dimethyl-glutamate increased the contents of M glutamate (no substitution with ^{13}C) but not M+2, M+3, M+4, or M+5 glutamate isotopomers (derived from [U-^{13}C]-glucose) in insulin granules as well as the cytosol, showing that dimethyl-glutamate is converted to glutamate within the granules as well as the cytosol.

(C) Effect of dimethyl-glutamate on dynamics of insulin granule exocytosis in primary cultured mouse β-cells. Distribution of fusion events of insulin granules (n = 4 for each). Insulin granule exocytosis occurs in three different modes, based on the dynamics of the insulin granules: Old face, granules that are predocked to the plasma membrane and fused to the membrane by stimulation; Resting newcomer, granules that are newly recruited, docked and fused to the plasma membrane by stimulation; and Restless newcomer, granules that are newly recruited and immediately fused to the plasma membrane by stimulation (Shibasaki et al., 2007).

(D and E) Effect of dimethyl-glutamate (5 mM), -malate (5 mM), or -succinate (5 mM) on insulin secretion under the glucose (16.7 mM)-stimulated condition in MIN6-K8 cells (D) and mouse pancreatic islets (E) (n = 4-8 for each).

The data are expressed as means ± SEM. Results are representative of 3 independent experiments. Welch's t test (A-C) or Dunnett's method (D and E) were used for evaluation of statistical significance. $^*p < 0.05$; $^{**}p < 0.01$; n.s., not significant.

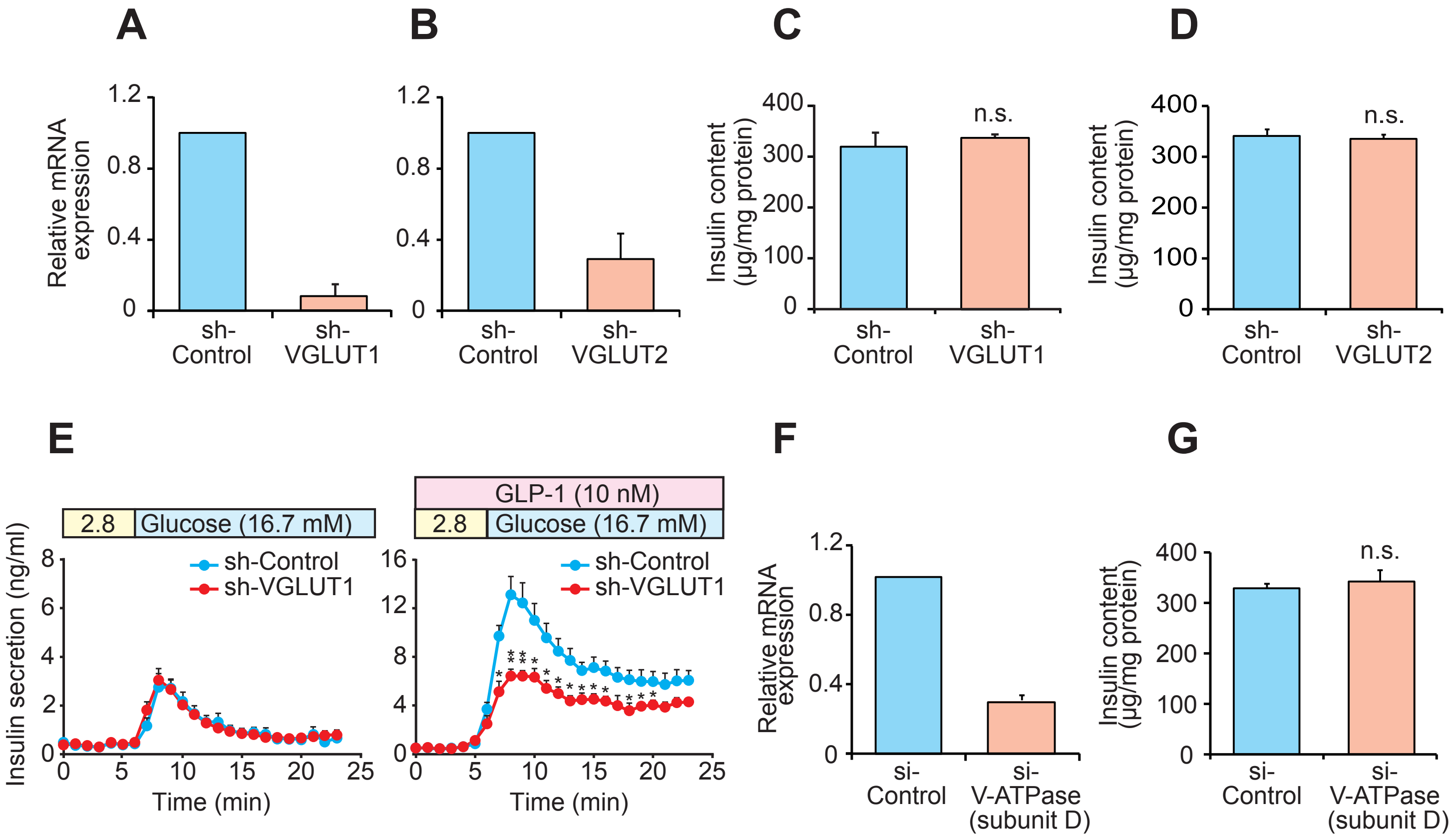

Figure S5. Effect of KD of VGLUT1, VGLUT2, or V-ATPase subunit D on mRNA Expression and Insulin Content and the Effect of KD of VGLUT1 on Dynamics of Insulin Secretion in MIN6-K8 Cells, related to Figure 5

(A and B) Relative mRNA expression levels of VGLUT1 (A) and VGLUT 2 (B) (n = 3 for each). mRNA expression levels in the respective KD cells are presented as relative to those in sh-Control cells, which are considered as 1.

(C and D) Effect of KD of VGLUT1 (C) or VGLUT 2 (D) on insulin contents (n = 3 for each).

(E) Effect of KD of VGLUT1 on dynamics of insulin secretion from perifused MIN6-K8 cells (n = 3-5 for each point). Glucose concentration was switched from 2.8 mM to 16.7 mM at 5 min in the absence (left) or presence (right) of GLP-1. "2.8" indicates 2.8 mM glucose.

(F) Relative mRNA expression level of V-ATPase subunit D (n = 3 for each). mRNA expression level in the KD cells is presented as relative to those in si-Control cells, which are considered as 1.

(G) Effects of KD of V-ATPase subunit D on insulin content (n = 3 for each).

The data are expressed as means ± SEM. Results are representative of 3 independent experiments. Welch's t test was used for evaluation of statistical significance (C-E and G). $*p < 0.05$; $**p < 0.01$; n.s., not significant.

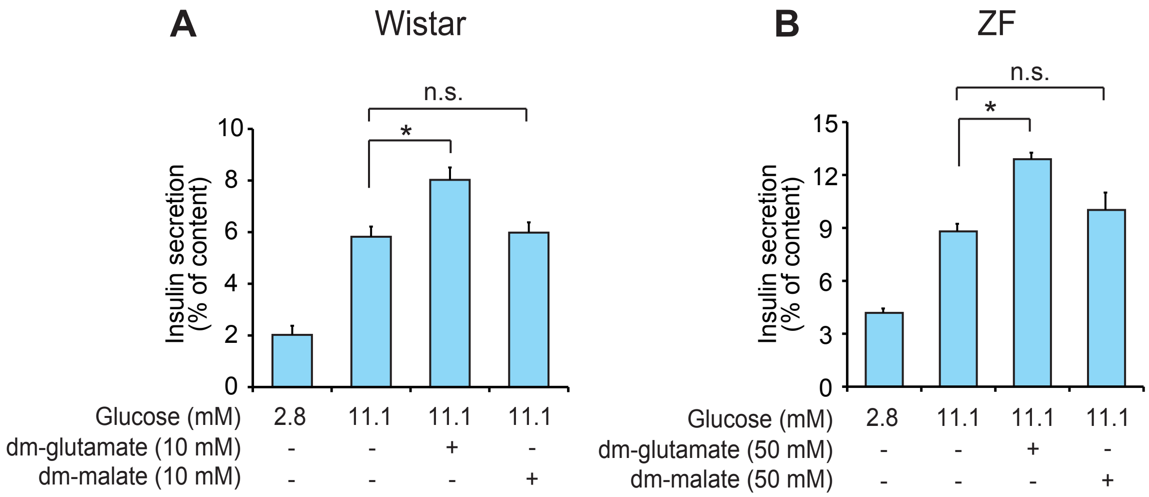

Figure S6. Effect of Dimethyl-Glutamate (10 or 50 mM) or -Malate (10 or 50 mM) on Insulin Secretion from Pancreatic Islets of Wistar (A) and ZF (Zucker Fatty) (B) Rats (n = 4-8 for each), related to Figure 6

The data are expressed as means ± SEM. Results are representative of 3 independent experiments. Dunnett's method was used for evaluation of statistical significance. $^*p < 0.05$; n.s., not significant.

Table S1. Comparative Metabolome Analysis of MIN6-K8 and -K20 β-Cell Lines, related to Figure 1

		Glucose (2.8 mM)		Glucose (16.7 mM)			
		Fold change	p-value	Fold change	p-value	C.V. (%)	I.S.
Glycolysis	Glucose 6-phosphate	1.04	0.819	2.34	*0.021	10.1	PIPES
	Fructose 6-phosphate	2.28	0.228	2.20	**0.010	5.4	PIPES
	Fructose 1,6-bisphosphate	0.73	0.180	3.03	**0.0003	15	PIPES
	Dihydroxyacetone phosphate	n.d.		n.d.		41.7	PIPES
	Glyceraldehyde 3-phosphate	n.d.		n.d.		9.5	PIPES
	1,3-bisphosphoglycerate	0.49	0.422	1.90	0.367	8.8	PIPES
	2- and 3-phosphoglycerate	0.83	0.730	0.63	0.282	9.3	PIPES
	Phosphoenolpyruvate	0.69	0.313	0.82	0.616	12.8	PIPES
TCA cycle	Acetyl CoA	n.d.		n.d.		7.3	PIPES
	Citrate	1.04	0.826	1.49	0.135	12.9	PIPES
	Isocitrate	n.d.		n.d.		12.6	PIPES
	α-ketoglutarate	n.d.		n.d.		10.6	PIPES
	Succinate	1.11	0.829	0.76	0.606	13.3	PIPES
	Fumarate	n.d.		n.d.		30.5	PIPES
	Malate	0.83	0.526	0.46	0.175	11.9	PIPES
Pentose phosphate pathway	6-phosphogluconate	1.00	0.996	1.10	0.696	14.1	PIPES
	Ribulose 5-phosphate	1.35	0.668	1.04	0.882	27.1	PIPES
	Ribose 5-phosphate	0.58	0.089	0.92	0.879	11.3	PIPES
	Sedoheptulose 7-phosphate	0.80	0.208	0.70	0.328	11.1	PIPES
	Erythrose 4-phosphate	n.d.		n.d.		11.5	PIPES
Glycogenesis	Glucose 1-phosphate	1.23	0.740	0.92	0.815	7.2	PIPES
	UDP-glucose	2.95	0.258	1.67	0.053	4.4	PIPES
Nucleotides	AMP	0.85	0.130	0.95	0.608	9.1	PIPES
	ADP	0.81	0.335	1.05	0.728	5.4	PIPES
	ATP	1.10	0.684	1.62	*0.045	7.9	PIPES
	cAMP	0.61	0.053	0.56	0.074	11	PIPES
	GMP	1.15	0.498	0.97	0.856	8.5	PIPES
	GDP	0.65	0.199	0.95	0.753	6.5	PIPES
	GTP	0.95	0.887	1.68	*0.020	10.6	PIPES
	NAD	1.09	0.624	1.36	0.082	14.1	PIPES
	NADH	3.54	0.051	4.81	**0.005	14.7	PIPES
	FAD	0.71	0.062	1.02	0.900	15.3	PIPES
	NADP	1.23	0.485	0.83	0.375	21.7	PIPES
	NADPH	0.61	0.060	0.83	0.318	8.9	PIPES
	ADP-ribose	n.d.		n.d.		12.2	PIPES
	cADP-ribose	n.d.		n.d.		11.5	PIPES
	UMP	1.01	0.934	0.85	0.381	7.8	PIPES
	UDP	0.89	0.655	1.83	**0.005	7.2	PIPES
	UTP	0.87	0.675	2.39	0.168	16.3	PIPES
	CMP	0.76	0.112	0.94	0.663	8.7	PIPES
	CDP	0.80	0.105	1.05	0.752	3.6	PIPES
	CTP	0.79	0.270	1.70	0.129	16.8	PIPES
	TMP	n.d.		n.d.		17.7	PIPES
	TDP	n.d.		n.d.		6.5	PIPES
	TTP	n.d.		n.d.		6.2	PIPES
Amino acids	Glutamate	1.84	*0.027	2.09	**0.005	11.1	$MetSO_2$
	Glutamine	0.93	0.712	1.22	0.377	10.9	$MetSO_2$
	Serine	0.78	0.444	0.97	0.921	21.3	$MetSO_2$
	Glycine	1.11	0.607	1.41	0.167	18.9	$MetSO_2$
	Arginine	1.71	0.130	1.19	0.557	15.5	$MetSO_2$
	Proline	0.84	0.439	1.17	0.347	15.3	$MetSO_2$
	Aspartate	1.68	0.174	3.11	**0.007	16.5	$MetSO_2$
	Asparagine	n.d.		n.d.		20.5	$MetSO_2$
	Alanine	1.24	0.516	1.58	0.098	21.9	$MetSO_2$
	Cysteine	n.d.		n.d.		n.d	
	Leucine	1.27	0.734	1.24	0.404	14.3	$MetSO_2$
	Isoleucine	0.72	0.394	1.03	0.895	20.1	$MetSO_2$
	Phenylalanine	1.06	0.904	1.00	0.990	11.5	$MetSO_2$
	Lysine	0.87	0.375	1.17	0.675	12.9	$MetSO_2$
	GABA	0.70	0.214	1.08	0.569	21.2	$MetSO_2$
	Threonine	0.94	0.793	0.89	0.673	23.4	$MetSO_2$
	Tyrosine	0.77	0.437	1.05	0.792	15.4	$MetSO_2$
	Tryptophane	0.89	0.794	1.06	0.773	11.7	$MetSO_2$
	Histidine	0.81	0.311	0.94	0.760	11.9	$MetSO_2$
	Valine	0.72	0.480	1.20	0.333	19.2	$MetSO_2$

Fold change for each metabolite was calculated by dividing content of metabolite of K8 cells by that of K20 cells (n = 3 each).

Welch's t test was used for evaluation of statistical significance. *p < 0.05; **p < 0.01.

Coefficient of variation (C.V.) for each metabolite was calculated from several repeated measurements of standard mixtures in the same batch.

I.S., internal standards. MetSO2, methionine sulfone. n.d., not detected.

Table S2. Comparative Metabolome Analysis of MIN6-K8 β-Cell Lines Stimulated by Glucose Alone and Glucose plus GLP-1, related to Figure 3

		Glucose (16.7 mM) + GLP-1 (10 nM)		Glucose (16.7 mM) + GLP-1 (100 nM)	
		Fold change	p-value	Fold change	p-value
Glycolysis	Glucose 6-phosphate and Fructose 6-phospahte	1.01	1.000	1.04	0.953
	Fructose 1,6-bisphosphate	1.29	0.626	1.14	0.972
	Dihydroxyacetone phosphate	n.d.		n.d.	
	Glyceraldehyde 3-phosphate	n.d.		n.d.	
	Glycelol 3-phosphate	1.14	0.790	1.00	1.000
	1,3-bisphosphoglycerate	n.d.		n.d.	
	2- and 3-phosphoglycerate	1.18	0.867	1.18	0.800
	Phosphoenolpyruvate	1.00	1.000	1.04	0.998
TCA cycle	Acetyl CoA	1.76	0.528	1.25	0.687
	Citrate	0.99	1.000	1.06	0.985
	Isocitrate	n.d.		n.d.	
	α-ketoglutarate	n.d.		n.d.	
	Succinyl CoA	n.d.		n.d.	
	Succinate	0.86	0.867	0.85	0.394
	Fumarate	n.d.		n.d.	
	Malate	1.05	0.996	1.20	0.786
Pentose phosphate pathway	6-phosphogluconate	1.44	0.650	1.07	0.958
	Ribulose 5-phosphate	n.d.		n.d.	
	Ribose 5-phosphate	n.d.		n.d.	
	Sedoheptulose 7-phosphate	1.12	0.995	0.72	0.768
	Erythrose 4-phosphate	n.d.		n.d.	
Glycogenesis	Glucose 1-phosphate	1.93	0.234	1.67	0.275
	UDP-glucose	1.16	0.965	1.58	0.252
Nucleotides	AMP	0.93	0.936	0.87	0.643
	ADP	1.28	0.781	1.25	0.681
	ATP	1.01	1.000	1.03	1.000
	cAMP	1.79	*0.027	1.91	*0.032
	GMP	0.93	0.992	0.83	0.942
	GDP	1.40	0.509	1.22	0.706
	GTP	1.86	0.518	1.41	0.585
	NAD	1.15	0.842	1.05	0.994
	NADH	1.02	0.100	0.95	0.999
	FAD	1.13	0.936	1.20	0.799
	NADP	1.09	0.929	0.99	1.000
	NADPH	n.d.		n.d.	
	ADP-ribose	1.50	0.669	0.70	0.529
	cADP-ribose	0.90	0.998	1.40	0.784
	UMP	1.15	0.920	0.85	0.846
	UDP	1.13	0.949	1.00	1.000
	UTP	1.49	0.645	1.25	0.559
	CMP	0.84	0.620	0.50	0.129
	CDP	1.23	0.768	0.93	0.972
	CTP	1.55	0.534	1.23	0.771
	TMP	n.d.		n.d.	
	TDP	n.d.		n.d.	
	TTP	1.66	0.242	0.88	0.980
Amino acids	Glutamate	0.98	0.999	0.97	0.996
	Glutamine	1.10	0.955	0.79	0.512
	Serine	1.16	0.677	2.91	0.247
	Glycine	1.01	1.000	1.19	0.737
	Arginine	0.84	0.893	0.98	1.000
	Proline	1.17	0.848	1.35	0.570
	Aspartate	1.05	0.926	1.07	0.943
	Asparagine	1.00	1.000	0.85	0.751
	Alanine	1.04	0.998	1.04	0.997
	Cysteine	n.d.		n.d.	
	Leucine	1.29	0.373	2.01	0.345
	Isoleucine	1.04	0.996	1.59	0.384
	Phenylalanine	1.07	0.979	1.61	0.471
	Lysine	0.92	0.987	1.31	0.518
	GABA	1.08	0.989	1.18	0.909
	Threonine	1.25	0.413	1.22	0.697
	Tyrosine	1.17	0.900	2.40	0.280
	Tryptophane	0.88	0.768	1.71	0.520
	Histidine	1.20	0.675	3.72	0.299
	Valine	1.11	0.924	1.84	0.382

Fold change for each metabolite was calculated by dividing content of metabolite of glucose plus GLP-1-stimulated K8 cells by that of glucose-stimulated cells (n = 3 each).

Dunnett's method was used for evaluation of statistical significance. *$p < 0.05$. n.d., not detected.

Supplemental Experimental Procedures

Reagents

Aminooxyacetate (AOA), dimethyl-glutamate, dimethyl-malate, dimethyl-succinate, and Evans blue were purchased from Tokyo Chemical Industry (Tokyo, Japan), Bafilomycin A1 was purchased from Wako (Osaka, Japan), [U-^{13}C]-glucose and H-89 were purchased from Sigma-Aldrich (St. Louis, MO), 8-pCPT-2'-*O*-Me-cAMP-AM (8-pCPT) and 6-Bnz-cAMP-AM (6-Bnz) were purchased from Biolog-Life Science Institute (Bremen, Germany), anti-PC1/3 antibody was purchased from Merck Millipore (Billerica, MA), Anti-cytochrome C antibody was purchased from Molecular Probes (Eugene, OR), and anti-VGLUT1 antibody (Hayashi et al., 2003) was provided by Y. Moriyama.

ATP Content

MIN6-K cells were preincubated for 30 min in HEPES-balanced Krebs-Ringer bicarbonate buffer containing 0.1% BSA (H-KRB) with 2.8 mM glucose and then incubated for 30 min in H-KRB with 2.8 mM or 16.7 mM glucose. After the incubation, these cells were lysed with cell culture lysis reagent (Promega, Wisconsin). The amount of ATP was measured with an ATP bioluminescence assay kit (Roche Diagnostics, Basel, Switzerland).

Intracellular Ca^{2+} Concentration

Cells were loaded with 5 μM fura-2 AM (Dojindo, Kumamoto, Japan) for 20 min in H-KRB with 0.1 mM glucose. Intracellular Ca^{2+} concentration was measured by a dual-excitation wavelength method (340/380 nm) with a fluorometer (Fluoroskan Ascent CF;

Labsystems, Helsinki, Finland). Data were expressed as the ratio of the fluorescence emission at 340/380 nm after background subtraction.

Metabolome Analysis

MIN6-K8 and -K20 cells (2.3 × 10^6 cells) were preincubated for 60 min in H-KRB with 2.8 mM glucose and then incubated for 30 min in H-KRB with 2.8 mM glucose or 16.7 mM glucose. After the incubation, internal standard solution [40 μl; ribitol (1 mM)/methionine sulfone (1 mM)/PIPES (100 mM)] and extraction solution [800 μl; acetonitrile/H_2O/CH_3Cl (7:3:10, v/v/v)] were added to the cells. The mixture was then homogenized and centrifuged at 20,000 × g under 4°C for 2 min. The supernatant containing hydrophilic primary metabolites was collected and dried in a centrifugal concentrator at room temperature. The dried hydrophilic metabolites were reconstituted with MilliQ water and then subjected to capillary electrophoresis-mass spectrometry (CE-MS) using a P/ACE MDQ (Beckman Coulter, Fullerton, CA) and 4000QTRAP hybrid triple quadrupole linear ion-trap mass spectrometer with Turbo V ion source and CE-MS kit (Applied Biosystems, Foster City, CA). CE-MS analysis was performed as previously described (Harada et al., 2008). Metabolite concentrations (pmol/2.3 × 10^6 cells) were calculated by using the ratio between each metabolite in the sample and standard compounds, and then results were presented as fold change over control.

Insulin Secretion

MIN6-K cells or pancreatic islets were preincubated for 30 min in H-KRB with 2.8 mM glucose and then incubated for 30 min in H-KRB with 2.8 mM glucose, 16.7 mM glucose, or 16.7 mM glucose plus reagents, such as GLP-1, GIP, dimethyl-glutamate, dimethyl-

malate, and dimethyl-succinate. In some experiments, AOA, bafilomycin, or Evans blue were added to the preincubation and incubation mediums. 0.3% FuGENE6 transfection reagent (Roche Applied Science, Mannheim, Germany) was also included in the mediums to increase efficiency of uptake of Evans blue by cells. Insulin released in the incubation buffer and cellular insulin content in MIN6-K cells or pancreatic islets were measured by insulin assay kits from CIS Bio international (Gif sur Yvette, France). The amounts of insulin secretion were normalized by the cellular insulin content determined by acid-ethanol extraction.

Knockdown Experiments

The following sequences that produced significant gene silencing were targeted:

AST1, 5'-GCCTATCAGGGCTTTGCATCT-3';

AST2, 5'-GCGTTACCGAAGCCTTCAAGA-3';

Aralar1, 5'-GCAGGAGTAGCTGATCAAACC-3';

GPD1, 5'-GCTAAATGGGCAGAAGCTACA-3';

GPD2, 5'-GCTCACAGGGCAGGAATTTGA-3';

VGLUT1, 5'-GCCATGGCATCTGGAGCAAAT-3';

VGLUT2, 5'-GCAAATCTGCTAGGTGCAATG-3'.

Non-target shRNA expressing adenoviral vector (negative control) was purchased from Invitrogen Corp (Carlsbad, CA). BLOCK-iT U6 RNAi entry vector and pAd/BLOCK-iT adenoviral vector plasmid (Invitrogen) were used for generation of adenoviruses, according to the manufacturer's instruction. MIN6-K8 cells were infected with adenovirus carrying shRNA.

For knockdown experiments of BCAT2, ALT2, GDH1, and V-ATPase subunit D,

siGENOME siRNA against these genes and siGENOME Non-Targeting siRNAs were purchased from Dharmacon (Lafayette, CO). MIN6-K8 cells were transfected with siRNAs using DharmaFECT2 transfection reagent (Dharmacon) according to the manufacturer's instructions.

Glutamate Content

For whole cell measurement, MIN6-K8 cells were stimulated for 30 min with various stimuli indicated in the figures. Glutamate contents in lysed cells were determined by Yamasa L-Glutamate Assay Kit II (Yamasa Corporation, Chiba, Japan). For measurement of mitochondrial and cytosolic glutamate, MIN6-K cells were collected after stimulations indicated in the figures and suspended in an isotonic buffer (0.32 M sucrose and 10 mM HEPES-KOH (pH 7.4)). The suspended cells were homogenized and centrifuged at 1000 × g at 4°C for 5 min. The post-nuclear supernatant was centrifuged at 27,000 × g at 4°C for 35 min. The pellet and supernatant were used for the measurement of mitochondrial and cytosolic glutamate contents, respectively. For measurement of glutamate content in insulin granules, insulin granules were obtained from MIN6-K cells as described previously (Bai et al., 2003). Insulin granule fraction was confirmed by immunoblot analysis using anti-PC1/3 antibody. Contents of glutamate were determined by Yamasa L-Glutamate Assay Kit II. Contents of glutamate isotopomers were also measured by ^{13}C-enrichment analysis with uniformly-labeled [U-^{13}C]-glucose as a substrate using CE-MS (Agilent 7100 CE and 6224 TOF LC-MS). Glutamate contents were normalized by protein contents determined by BCA protein assay kit (Thermo Scientific, Waltham, MA) in MIN6-K cells or by DNA contents determined by Quant-iT PicoGreen dsDNA Assay Kit (Invitrogen) in pancreatic islets.

Electrophysiology

Capacitance recordings of single β-cell exocytosis were made in primary mouse β-cells using the standard whole-cell configuration in which the pipette-filling solution dialyzes the cell interior, allowing cAMP and glutamate to be applied intracellularly. The extracellular medium consisted of 118 mM NaCl, 20 mM TEA-Cl, 5.6 mM KCl, 1.2 mM $MgCl_2$, 2.6 mM $CaCl_2$, 5 mM D-glucose, and 5 mM HEPES (pH 7.4 with NaOH). The standard electrode (intracellular) solution contained 90 mM Cs-gluconate, 35 mM CsCl, 1 mM $MgCl_2$, 5 mM HEPES, 0.05 mM EGTA, 3 mM Mg-ATP (pH 7.15 with CsOH). Cs-glutamate was included at concentrations of 1-10 mM gluconate and Cs-gluconate was correspondingly reduced, to maintain iso-osmolarity. Na-cAMP and the PKI (both from Sigma) were included in the intracellular (pipette-filling) medium at the indicated concentrations. The depolarizations were 500 ms long, applied at a frequency of 1 Hz and went from -70 mV to 0 mV.

Immunofluorescence Staining

MIN6-K8 cells were fixed and pretreated as previously described (Yasuda et al., 2010). C57BL/6 mice were perfused transcardially with fixative solution containing 2% formaldehyde in 0.1 M phosphate buffer (pH 7.4). The pancreases were isolated and immersed in the same fixative solution for 5 hours at 4°C. Frozen sections were prepared by a cryostat (Leica, Bensheim, Germany). Cells and tissues were incubated with guinea pig anti-insulin antibody and rabbit anti-VGLUT1 antibody, followed by Alexa Fluor 488-conjugated goat anti-guinea pig IgG antibody and Alexa Fluor 546-conjugated goat anti-rabbit IgG antibody, respectively.

Phosphorylation of CREB and Activation of Rap1

Measurements of phosphorylation of CREB and activation of Rap1 were performed as previously described (Shibasaki et al., 2007; Zhang et al., 2009).

Enzyme Activity

MIN6-K8 cells were incubated for 10 min in the absence or presence of 10 nM GLP-1 with 16.7 mM glucose. The cells were collected and suspended in the isotonic buffer containing protease inhibitors (Complete, EDTA Free; Roche Applied Science). The cells were homogenized and centrifuged at 1,000 × g at 4°C for 5 min. The post-nuclear supernatant (PNS) was centrifuged at 27,000 × g at 4°C for 35 min. The supernatant was used for the measurement of activities of AST1 and MDH1. AST1 activity was determined by Aspartate Transaminase Enzymatic Assay Kit (Bio Scientific, Austin, TX). Activity of MDH1 was measured as previously described (Glatthaar et al., 1974).

Perifusion Experiments

Perifusion experiments on insulin secretion of MIN6-K8 cells were performed as described previously (Sugawara et al., 2009).

Supplemental References

Glatthaar, B.E., Barbarash, G.R., Noyes, B.E., Banaszak, L.J., and Bradshaw, R.A. (1974). The preparation of the cytoplasmic and mitochondrial forms of malate dehydrogenase and aspartate aminotransferase from pig heart by a single procedure. Anal. Biochem. *57*, 432-451.

Harada, K., Ohyama, Y., Tabushi, T., Kobayashi, A., and Fukusaki, E. (2008). Quantitative analysis of anionic metabolites for Catharanthus roseus by capillary electrophoresis using sulfonated capillary coupled with electrospray ionization-tandem mass spectrometry. J. Biosci. Bioeng. *105*, 249-260.

Hayashi, M., Morimoto, R., Yamamoto, A., and Moriyama, Y. (2003). Expression and localization of vesicular glutamate transporters in pancreatic islets, upper gastrointestinal tract, and testis. J. Histochem. Cytochem. *51*, 1375-1390.

Zhang, C.L., Katoh, M., Shibasaki, T., Minami, K., Sunaga, Y., Takahashi, H., Yokoi, N., Iwasaki, M., Miki, T., and Seino, S. (2009). The cAMP sensor Epac2 is a direct target of antidiabetic sulfonylurea drugs. Science *325*, 607-610.
